# Main Group Molecular Switches with Swivel Bifurcated to Trifurcated Hydrogen Bond Mode of Action

**DOI:** 10.1021/jacs.2c12713

**Published:** 2023-06-02

**Authors:** Gavin Hum, Si Jia Isabel Phang, How Chee Ong, Felix León, Shina Quek, Yi Xin Joycelyn Khoo, Chenfei Li, Yongxin Li, Jack K. Clegg, Jesús Díaz, Mihaiela C. Stuparu, Felipe García

**Affiliations:** † School of Chemistry, Chemical Engineering & Biotechnology, Nanyang Technological University, 21 Nanyang Link, 637371 Singapore, Singapore; ‡ School of Chemistry and Molecular Biosciences, The University of Queensland, Cooper Road, St Lucia 4072, Queensland, Australia; § Departamento de Química Orgánica e Inorgánica, Facultad de Veterinaria Extremadura, Avda de la Universidad s/n, Cáceres 10003, Spain; ∥ Departamento de Química Orgánica e Inorgánica, Facultad de Química, Universidad de Oviedo, Julián Claveria 8, Oviedo 33006, Asturias, Spain; ⊥ School of Chemistry, Monash University, Clayton 3800, Victoria, Australia

## Abstract

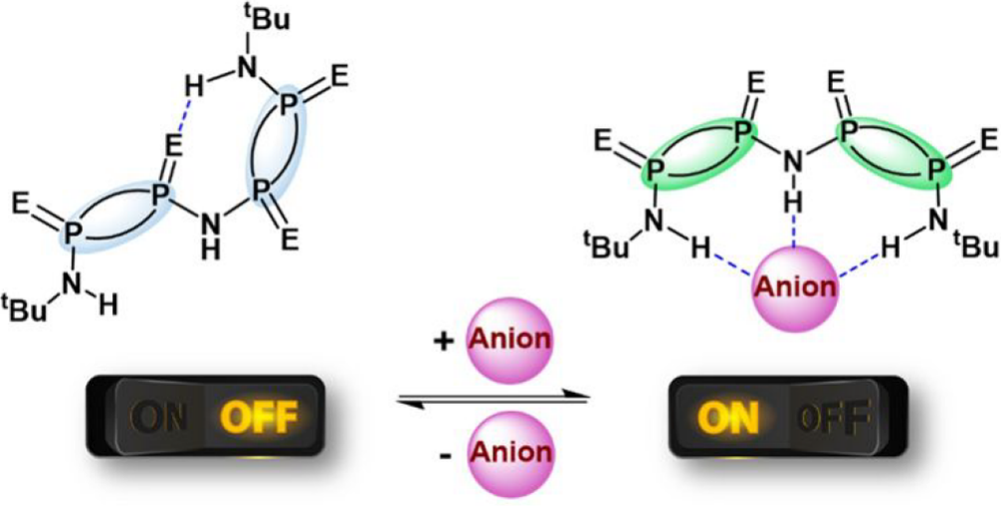

Artificial molecular machines have captured the full attention of the scientific community since Jean-Pierre Sauvage, Fraser Stoddart, and Ben Feringa were awarded the 2016 Nobel Prize in Chemistry. The past and current developments in molecular machinery (rotaxanes, rotors, and switches) primarily rely on organic-based compounds as molecular building blocks for their assembly and future development. In contrast, the main group chemical space has not been traditionally part of the molecular machine domain. The oxidation states and valency ranges within the p-block provide a tremendous wealth of structures with various chemical properties. Such chemical diversity—when implemented in molecular machines—could become a transformative force in the field. Within this context, we have rationally designed a series of NH-bridged acyclic dimeric cyclodiphosphazane species, [(μ-NH){PE(μ-N^t^Bu)_2_PE(NH^t^Bu)}_2_] (E = O and S), bis-P^V^_2_N_2_, displaying bimodal bifurcated R^2^_1_(8) and trifurcated R^3^_1_(8,8) hydrogen bonding motifs. The reported species reversibly switch their topological arrangement in the presence and absence of anions. Our results underscore these species as versatile building blocks for molecular machines and switches, as well as supramolecular chemistry and crystal engineering based on cyclophosphazane frameworks.

## Introduction

The last three decades have seen the rise of rationally designed synthetic molecular architectures with stimuli-controlled molecular-level motion. These advances have allowed humanity to build artificial structures that can control and exploit molecular-level motion, giving rise to a wide range of molecular machines and switches.^1−7^

There has been a wide range of reported examples of molecules, including catenanes and rotaxanes, where molecular motion is triggered by various stimuli (light,^8−12^ electrochemistry,^13−18^ pH,^19−22^ heat,^23,24^ solvent polarity,^25,26^ cation,^27−29^ anion binding,^30−35^ etc.). Despite the extraordinary progress already made, researchers have merely scratched the surface, and further developments are required to reach the level of competence/sophistication displayed by biological systems.^36−40^

Future molecular machines will require a multidisciplinary approach with inputs and expertise from other fields, which necessitates the development of molecular machinery based on main group backbones (i.e., based on non-carbon–carbon bonds). Over the last few decades, the field of main group chemistry has not only contributed to chemistry at large by providing important chemical concepts,^41^ but also has uncovered a wealth of catalytic systems^42−44^ and energy materials.^45^ However, its implementation in the area of molecular machines is still lagging behind their organic counterparts.

**Figure 1:**
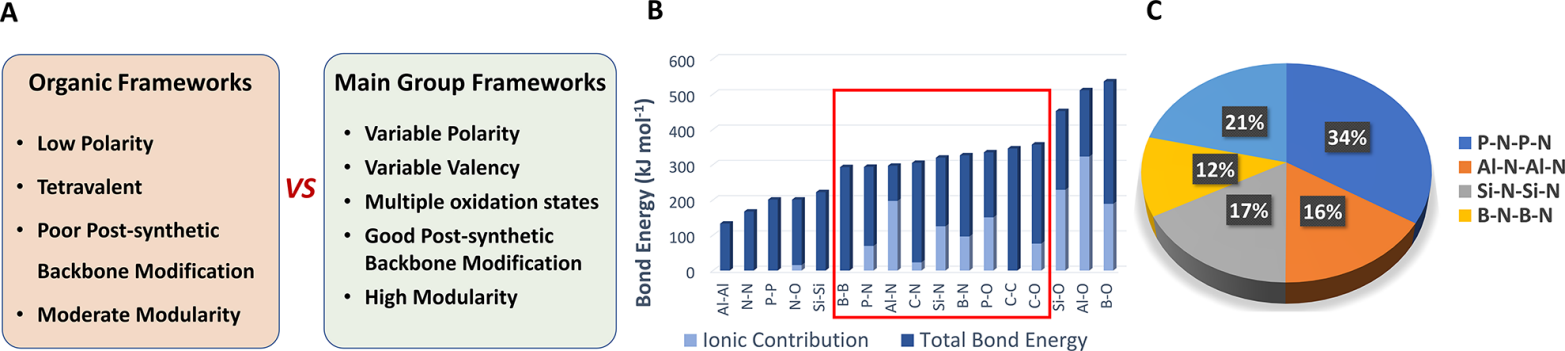
Comparison between organic and main group frameworks. (A) Typical properties of organic and main group backbones. (B) Total bond energy and ionic contribution for selected homoatomic and heteroatomic bonds comprising p-block elements. (C) Percentage of crystallographically characterized main group compounds comprising an A-B-A-B backbone (data obtained from CCDC database accessed on March 17, 2022).

In contrast to carbon-based systems, where the tetravalent state dominates the chemistry, other main group elements display multiple valencies and oxidation states with very specific reactivity and chemical properties (Figure 1A). Therefore, main group elements provide a wealth of unexplored backbones in the molecular machinery space, which we envision will impact how future molecular machines are designed.

However, before selecting a suitable main group system, the kinetic and thermodynamic stability of the non-carbon bonds must be considered (Figure 1B). The ideal main group frameworks are those with high bond energies (relative to carbon–carbon bonds) and display low bond polarities (Figure 1B).^46^ Within this context, among the potential main group families (i.e., P–N, Al–N, Si–N, B–N, P–O, etc.), P–N bonds fulfill both requirements, displaying comparable bond energies to carbon-based species (i.e., C–C, C–O, and C–N), as well as a low polarity. In addition, P–N-based backbones are the largest family of main group compounds displaying these requirements, making them the ideal frameworks for proof-of-concept studies (Figure 1C).

Among the molecular motifs comprising P–N bonds, P_2_N_2_ cyclodiphosphazane building blocks are capable of forming a broad range of cyclic and acyclic frameworks. These main group systems^47^ have shown to be excellent ligands for metal coordination^48−55^ and versatile modular building blocks for the construction of larger molecules for biological applications and supramolecular chemistry.^56−65^

A powerful motif used in the design of molecular machines,^66−70^ organocatalyst,^71,72^ anion sensors,^73^ anion transporters *inter alia*(74) is the hydrogen bond (HB). In particular, excellent HB donors such as ureas, thioureas, and squaramides^75−78^ are commonly used building blocks in molecular machines due to their bifurcated NH geometry and the tunable acidity of their amino protons via substituent modification.^1,2,79^

More recently, it has been demonstrated that cyclodiphosphazanes,^47^ P_2_N_2_ frameworks, are versatile HB donors that rival ureas, thioureas, and even squaramides, thanks to their increased bite angles.^63−65^ In addition, these species have been shown to effectively bind to small molecules [e.g., acetone, dimethyl sulfoxide (DMSO), and dimethylformamide] and be versatile building blocks for the engineering of high-order ternary and quaternary multicomponent cocrystals, which further broadens the scope of their applications in supramolecular applications and crystal engineering.^59,80^

Common strategies for improving the HB donor ability of urea and thioureas for their use as building blocks in both supramolecular chemistry and molecular machines are to increase (i) the complexity (i.e., adding extra HB functionalities, electron-withdrawing groups, etc.) of the substituents (**Approach 1**)^81−83^ or (ii) the number of repeating units of urea/thiourea crafted within the molecular backbone (**Approach 2**).^84−90^ Both strategies aim to increase HB ability, translating into better binding affinities and performance versus their classical counterparts.

**Scheme 1:**
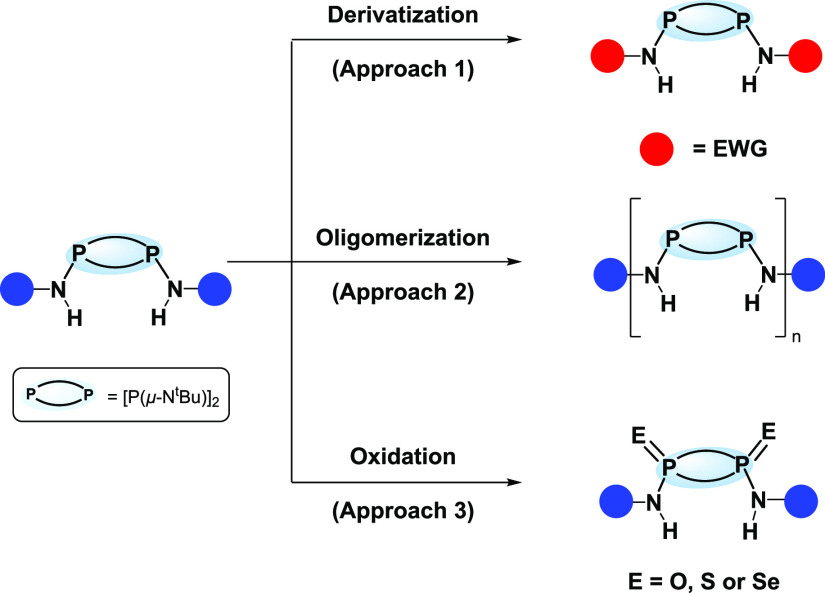
Strategies for Improving the HB Donor Ability of P_2_N_2_ Species

Similar approaches can—in theory—be applied to P_2_N_2_ species (see Scheme 1). The influence of varying substituents has been studied for monomeric species (i.e., **Approach 1**).^63−65^ However, in contrast to carbon-based frameworks, it was found that effective HB abilities are only observable after the P^III^_2_N_2_ backbone of choice is oxidized to P^V^_2_N_2_ (i.e., from P^III^ to P^V^). Notably, the oxidation process P^III^_2_N_2_ to P^V^_2_N_2_ enables further fine-tuning of their HB ability (**Approach 3**), since various chalcogen elements (i.e., O, S, or Se) can be readily installed onto the backbone during a simple post-synthetic oxidation step.^91−96^ Notably, this “gain of function” feature—that is, the one-step installation of chalcogen elements—is not readily available for widely used carbon-based building blocks, which limits the fine-tuning of their properties and reduces their scope through post-synthetic backbone alteration.

In terms of increasing the number of repeating units to form oligomeric P_2_N_2_ species (i.e., **Approach 2**), this approach has been traditionally impaired due to the lack of selectivity between cyclic and acyclic oligomeric P^III^_2_N_2_ species containing NH moieties.^97−99^ However, novel topologically tunable N-bridged acyclic oligo-P^III^_2_N_2_ (i.e., dimeric and trimeric) species have been recently reported.^100^ The latter comprise different substituents (e.g., H, ^i^Pr, Ph, and ^t^Bu) at the two backbone bridging positions, which determine their final topological conformation. This report represents the first rational selection of different topological conformations using non-covalent interactions in the phosphazane P^III^_2_N_2_ family. In addition, theoretical studies predict acyclic dimeric- and trimeric-P^V^_2_N_2_ species as topologically tunable frameworks with superior halide receptors with increased binding ability toward chlorides compared to their monomeric counterparts (i.e., squaramide and thiourea R^2^_1_(8) type building blocks).^100^ It is worth noting that anion binding has been successfully used as a chemical stimulus in a wide range of molecular switches.^30,31,33,101−103^

The previously demonstrated rational selection of different topological conformations in dimeric and trimeric acyclic P^III^_2_N_2_ phosphazane species, combined with the predicted superior halide binding ability of P^V^_2_N_2_, suggests their suitability as potential main group building blocks toward chemically responsive frameworks based on a fully inorganic backbone.

Herein, we report the synthesis of novel acyclic NH-bridged dimeric-P^V^_2_N_2_ species and demonstrate them as effective molecular switches activated by anionic species. This new family of NH-bridged P^V^_2_N_2_ molecular switches feature both topological responsiveness to external anion stimuli and adaptable cavity size. We envision that these unique properties will enable oligomeric cyclodiphosphazane frameworks to play a crucial role in designing molecular machines, host–guest systems, and supramolecular chemistry in the future.

## Results

### Synthesis and Characterization of Acyclic Dimeric-P^V^_2_N_2_ Molecular Switches

The basic building block in our studies, compound **1**, can be obtained via the single-step reaction of Cl[P(μ-N^t^Bu)]_2_NH^t^Bu with LiNH_2_ in THF at room temperature (Scheme S1).^100,104^ This recently reported synthetic methodology allows for a simple and straightforward route to NH-bridged acyclic dimeric-P^III^_2_N_2_.^100^

**Figure 2:**
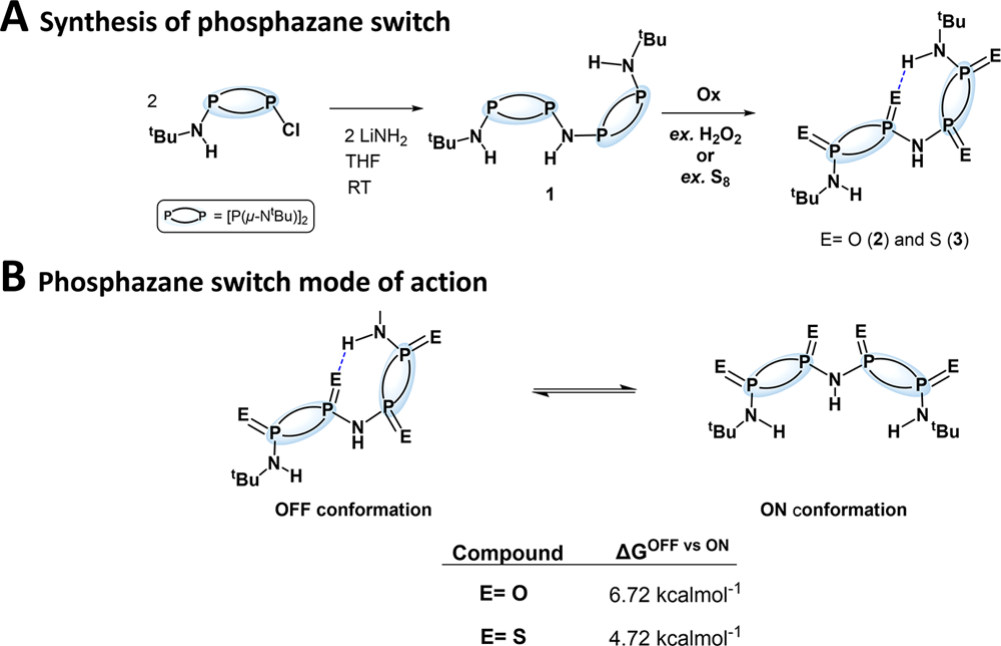
Synthesis of dimeric-P^V^_2_N_2_ species and their switch mode of action. (A) Synthetic route to phosphazane molecular switches. (B) Phosphazane switch **OFF** to **ON** (i.e., **S** to **C**) topological conformational change mode of action observed in solution for **2** and **3**—the energy differences displayed were calculated by DFT using B3pw91/6-311 g(d,p) basis set.

Compound **1** was then oxidized to form the NH-bridged acyclic dimeric-P^V^_2_N_2_ counterpart to enable HB ability (Figure 2A). Treatment of **1** with six equivalents of H_2_O_2_, added dropwise at 0 °C, afforded its oxygen oxidized counterpart **2** (Scheme S2). The ^1^H NMR spectrum of **2** in CDCl_3_ exhibits three different NH signals at 3.63, 5.36, and 7.39 ppm and two different tert-butyl signals at 1.34 and 1.47 ppm for terminal positions, revealing the asymmetric nature of compound **2** (Figure S4). This suggests that compound **2** adopts a twisted exo,endo/exo,exo “**S**” conformation (i.e., **2^OFF^**), in which both P^V^_2_N_2_ fragments are non-equivalent. This feature was attributed to the presence of intramolecular hydrogen bonding within the dimeric backbone. Furthermore, the ^31^P–{^1^H} NMR of **2** in CDCl_3_ also shows two heavily broadened signals at −1.11 and −6.66 ppm, which further suggests the existence of intramolecular P=O···H–N HB interactions in **2^OFF^** (Figure S5).

Similarly, the overnight reaction of **1** with 4.2 equivalents of elemental sulfur in THF at room temperature furnished **3** (Scheme S3). In contrast to compound **2**, the ^1^H NMR in CDCl_3_ for **3** reveals two NH signals at 4.12 and 5.16 ppm with a ratio of 2:1, while the two terminal tert-butyl groups are also equivalent, giving rise to only one signal at 1.44 ppm (Figure S9), suggesting an exo,exo/exo,exo “**C**” conformation (i.e., **3^ON^**). However, compound **3** is expected to favor the **3^OFF^** conformation ([**3^ON^**]/[**3^OFF^**] < 0.01%) according to density functional theory (DFT) calculations (vide infra, Figure 2, Figure 4, and Supporting Information). Therefore, the observed ^1^H NMR spectrum suggests a fluxional behavior of compound **3**, with a rapid interconversion between **3^ON^** and **3^OFF^**. In addition, the ^31^P–{^1^H} NMR spectrum shows significantly sharper signals at 34.28 and 41.93 ppm relative to compound **2** (Figure S11), supportive of much weaker HB interactions with the P=S moiety.

To confirm this hypothesis, variable temperature (VT) ^1^H NMR of **3** was performed, and two broad singlets at 3.34 and 5.15 ppm in a 1:2 ratio were observed at 213 K (cf. 4.12 and 5.16 ppm in a 2:1 ratio at RT, see Figure S14), which is indicative of a “**S**” topological arrangement (i.e., **3^OFF^**). The presence of only two resonances in the NMR spectrum—instead of the three that would have been expected—combined with the observed inversion of the signal ratio, is attributed to small differences in the chemical shift, which results in the coincidental overlapping of two of the three distinct NH environments in **3^OFF^**. Determination of the energy barrier of rotation based on the VT ^1^H NMR data (*T*_C_ = 253 K, Δν = 722 Hz) suggests a relatively low rotation barrier between the **3^ON^** and **3^OFF^** conformations (∼11.0 kcal·mol^–1^). This experimental value is in good agreement with the DFT-calculated rotation barrier of 12.57 kcal·mol^–1^ (Figure 2B, see Figures S15 and S69 for details), where the **3^ON^** conformer is calculated to be 4.72 kcal·mol^–1^ above the **3^OFF^** counterpart (Figures 2B and S69).

To confirm if the same **ON/OFF** conformational changes can be observed for compound **2**, high-temperature NMR studies were performed. Upon heating to 333 K, the signal displayed by compound **2** broadened substantially, as the equilibrium between **2^ON^** and **2^OFF^** approaches the NMR timescale (Figure S12). However, the coalescence temperature could not be achieved in the solvent system used (b.p. CDCl_3_ = 334.5 K), indicating that compound **2** has a higher **ON/OFF** rotational barrier than **3**. To further confirm this, the same experiment was conducted in tetrachloroethane-d_2_, allowing for higher temperatures to be achieved. Indeed, fluxional behavior was observed upon heating to 413 K, with terminal NH resonances converging into a single signal at 4.08 ppm (cf. 3.62 and 5.42 ppm at RT, see Figure S13). Calculations based on coalescence temperature achieved in ^1^H high-temperature NMR studies (*T*_C_ = 353 K, Δν = 720 Hz) saw higher rotational barrier energy between **2^ON^** and **2^OFF^** conformations (∼15.6 kcal·mol^–1^) as compared to **3,** which further confirms our hypothesis. The different **ON/OFF** behavior between compounds **2** and **3** observed is attributed to the different HB strengths present in these species, where only the stronger P=O···H–N is sufficient at room temperature to prevent fluxionality.

To assess the effect of HB donor/acceptor solvents on **2^OFF^**, where the **OFF** conformation is “locked” in place by intramolecular HB interactions, the compound was dissolved in methanol-d_4_, and its ^1^H NMR spectrum was recorded. The spectrum shows the absence of NH signals, which is attributed to peak broadening due to HB interactions of the amino protons with the methanol-d_4_. Notably, there is only one resonance at 1.38 ppm corresponding to terminal tert-butyl groups (cf. δ 1.34 and 1.47 ppm in CDCl_3_), which suggests that competing HB interactions disrupt the intramolecular P=O···HN^t^Bu HB in **2**, allowing for fluxional behavior in methanol-d_4_ (Figures S4–S8).

Despite the successful synthesis of **2** and **3**, the reaction of **1** with 4.2 equivalents of elemental selenium in THF did not result in the formation of expected selenium-oxidized acyclic dimeric-P^V^_2_N_2_, [(μ-NH){PSe(μ-N^t^Bu)_2_PSe(NH^t^Bu)}_2_] (**4**) (Scheme S4). Instead, the in situ ^31^P–{^1^H} NMR spectrum reveals a mixture of products. Further insights on the reaction were gained from diffraction quality crystals obtained from a concentrated toluene solution, where [H_2_N[P(Se)(μ-N^t^Bu)]_2_NH^t^Bu] (**4a**) was identified as one of the products (Figure S67). Notably, this compound is the first crystallographically characterized asymmetrically substituted monomeric-P^V^_2_N_2_ containing an −NH_2_ moiety.

An important property for implementing main group frameworks in molecular machinery is their overall stability under ambient conditions. Compounds **2** and **3** both display high air and hydrolytic stability, and can be bench-stored and handled under ambient atmospheric conditions.^105−108^ Hydrolytic studies of samples containing **2** and **3** each in 1:9 H_2_O/THF monitored via ^31^P–{^1^H} NMR showed no signs of degradation for up to 4 weeks, showcasing their robustness as extended phosphazane scaffolds for a multitude of supramolecular chemistry applications (Figures S16 and S17).^105,106^

### P^V^_2_N_2_ Switch OFF Mode: R^2^_1_(8) Bifurcated HB to Neutral Guest Molecules

**Figure 3:**
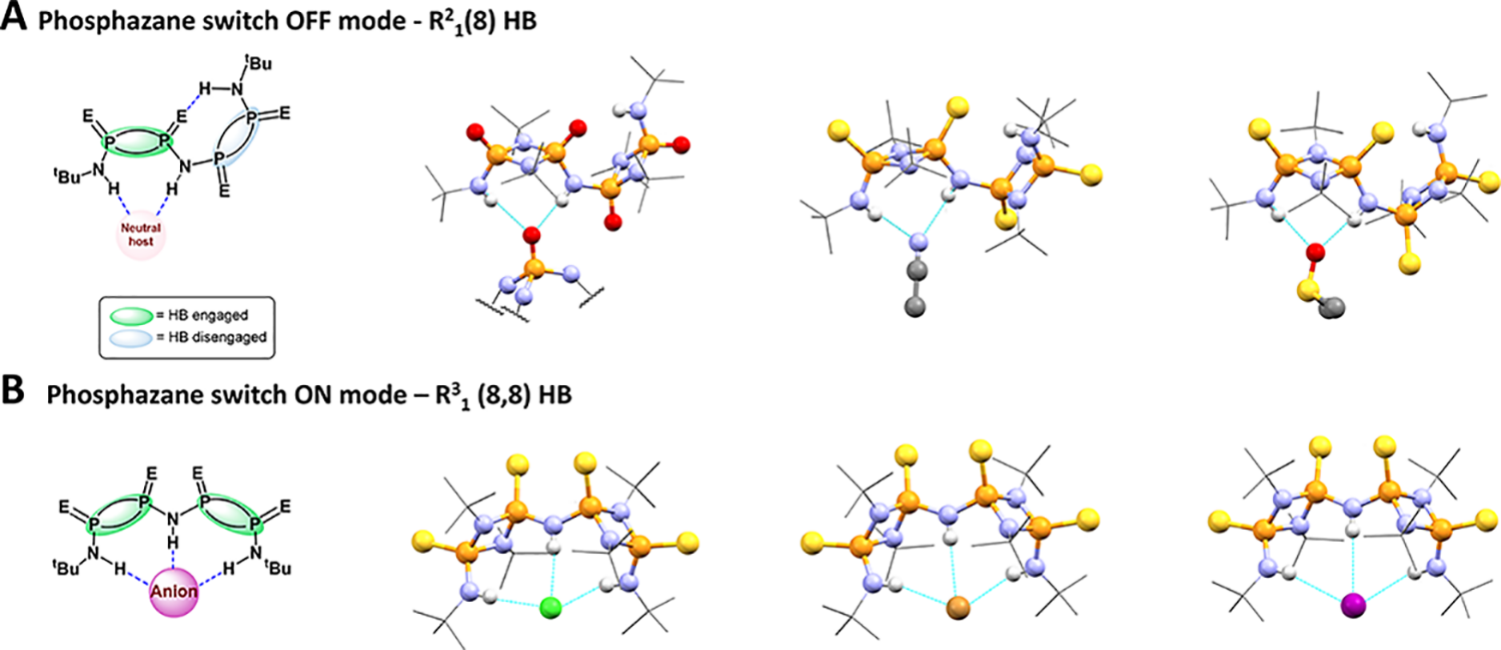
Molecular switch binding modes to neutral and anionic guests. (A) Dimeric-P^V^_2_N_2_ switch **OFF** mode (**3^OFF^**) – R^2^_1_(8). Solid-state structures of “**2** ⊂ **2**” (displaying a fragment of the supramolecular dimer in the solid state, left), **3** ⊂ Acetonitrile (middle), and **3** ⊂ DMSO (right), illustrating weak interactions with neutral molecules. Hydrogen atoms (except selected NH protons) and disorder were omitted for clarity. (B) Dimeric-P^V^_2_N_2_ switch **ON** mode (**3^ON^**) – R^3^_1_(8,8). Solid-state structures of **3** ⊂ Cl^–^ (left), **3** ⊂ Br^–^ (middle), and **3** ⊂ I^–^ (right). Hydrogen atoms (except selected NH protons), and disorder were omitted for clarity. For selected bond distances and expanded figures, see Supporting Information.

To further confirm the structures of compounds **2** and **3** and their **ON/OFF** topologies, single crystal X-ray diffraction studies were performed. These studies reveal both compounds adopting an **OFF** twisted topology in the solid state, comprising a R^2^_1_(8) bifurcated hydrogen interactions. (Figures 3A, S59–S62).^59,65^

The crystal of **2** obtained from a THF solution displays strong intermolecular interactions, leading to the formation dimeric aggregate via R^2^_1_(8) bifurcated HB interactions between the amino protons and the P=O group of two molecules of **2** (Figure S59). In chloroform (i.e., a solvent comprising of the HB donor), the same bimolecular aggregate is observed, as well as exogenous P=O···H–CCl_3_ HB interactions. In both structures, the observed HB bond distances are 2.87 to 3.19 Å, which is consistent with monomeric counterparts.^65^ In contrast to the formation of HB dimers, the solid-state structure of **3** reveals the formation of R^2^_1_(8) bifurcated HB solvates (with MeCN and DMSO) (Figures S61 and S62). These types of HB interactions with neutral organic molecules, and their bond distances, are consistent with those observed in monomeric P^V^_2_N_2_ counterparts.^59^

The formation of dimers in **2**—instead of monomeric solvates as observed for compound **3**—is attributed to the preferential formation of strong bifurcated R^2^_1_(8) HB with a P=O moiety over a molecule of THF. Overall, both compounds display an **OFF** conformation, where the second P^V^_2_N_2_ unit does not engage in intermolecular HB (Figure 3A), which demonstrates a preference for intramolecular R^2^_1_(8) HB in the presence of neutral molecules over an ON R^3^_1_(8,8) HB motif where all the NH groups are engaging in bonding.

**Figure 4:**
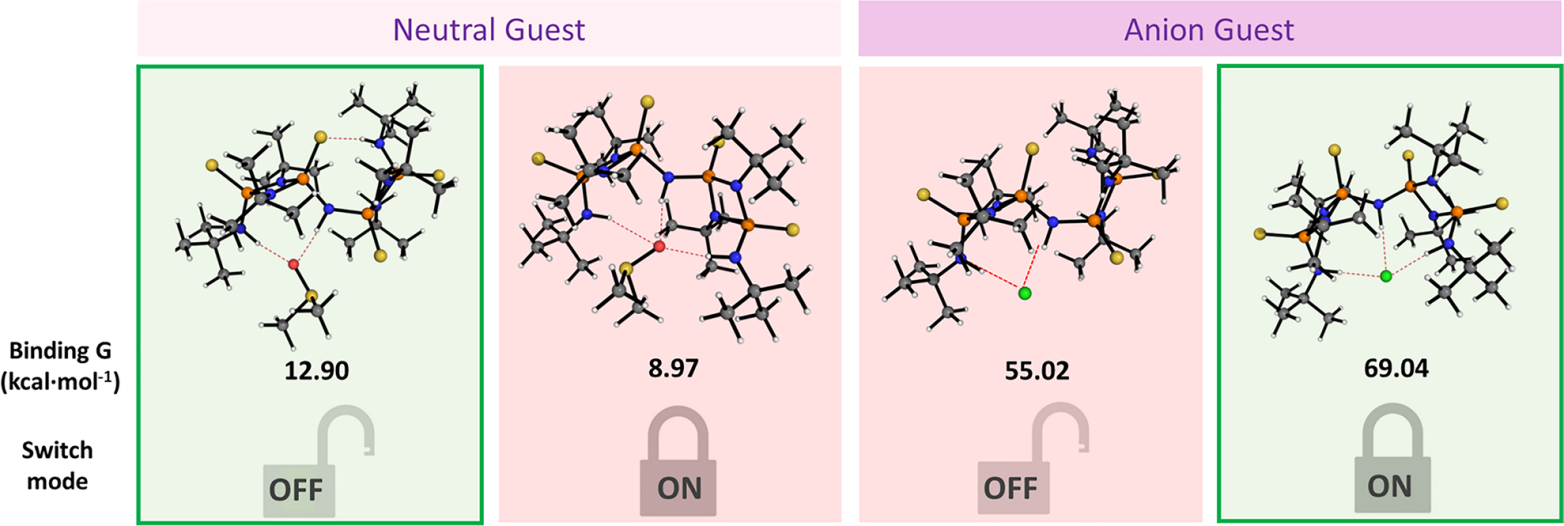
Calculated binding energies and preferred switch model for the **3^OFF^** and **3^ON^** solvates with DMSO and Cl anions at B3pw91/6-311 g(d,p) level of theory.

To gain further insights into the different **ON/OFF** behavior, DFT studies at (B3pw91/6-311 g(d,p) level of theory) were performed. The binding energy for the coordination of a molecule of DMSO to **3^OFF^** was calculated to be 12.90 kcal·mol^–1^. In contrast, the binding energy for the **3^ON^** topological conformation was calculated to be 8.97 kcal·mol^–1^ relative to the individual molecules (Figure 4). The differences in stabilization between the **ON/OFF** HB motifs (ca. 4 kcal/mol) are attributed to the different strengths of the non-covalent interactions present (i.e., R^2^_1_(8) vs P=S···H–N HB interactions; see Figures S69–S71 for non-covalent interactions). In the **OFF** conformation, one R^2^_1_(8) HB and one P=S···H–N are present. In contrast, its **ON** counterpart displays a trifurcated HB interaction with two symmetric adjacent bifurcated R^2^_1_(8) HB interactions “sharing” a HB donor to a common acceptor (i.e., in the same manner, adjacent angles are mathematically defined), which we define as a R^3^_1_(8,8) interaction. The stronger intramolecular HB interaction in compound **2** ⊂ DMSO has also been computed, and the energy difference between the **ON/OFF** conformation is calculated to be ca. 7.6 kcal/mol (Table S6). In addition, we performed non-covalent interaction analyses for compounds **2** and **3** on their **OFF** conformation (Figure S71). Our analyses show that **2^OFF^** displays stronger attractive intramolecular HB interactions than **3^OFF^**, which further supports our hypothesis and is consistent with the experimental observations.

### P^V^_2_N_2_ Switch ON Mode: R^3^_1_(8,8) Trifurcated HB to Anions Hosts

As observed, interactions of **2** and **3** with small neutral organic molecules favor **OFF** conformations, displaying bifurcated R^2^_1_(8) HB modes. However, the second P^V^_2_N_2_ unit does not engage in intermolecular HB, which is attributed to the presence of the intramolecular P=E···NH HB present in compounds **2** and **3**.

Past reports on monomeric cyclodiphosphazane receptors have shown that strong HB guests, such as halide anions, favor the exo,exo conformation,^80,100^ which enables these species to act as R^2^_1_(8) HB donors.^60,64,65^ This preference for the exo,exo (over the exo,endo) in the presence of halide HB acceptors has also been recently highlighted during the formation of high-order multicomponent cocrystals based on monomeric P^V^_2_N_2_ building blocks.^80^

The presence of an additional P^V^_2_N_2_ provides **2** and **3** with an additional degree of freedom (i.e., **OFF** vs **ON** conformations) and could potentially provide a superior performance toward anion binding and sensing if selectively switched **ON**. Moreover, rationally controlling rotatory motion around a single bond has been commonly used in molecular machines and switches using a wide range of organic (i.e., triptycyl, quinoline, napthyl moieties, etc.) and organometallic molecular architectures triggered by various chemical stimuli (e.g., protonation, anions, and cations, inter alia).^1,2,79^ We postulate that in contrast to what was observed for neutral molecules (i.e., MeCN and DMSO), where only the **OFF** conformation is observed, the second unit would switch **ON** in the presence of anionic hosts via the formations of higher-order trifurcated R^3^_1_(8,8) HB interactions, hence fulfilling the conformational changes required to be classified as a molecular switch.^40^

**Figure 5:**
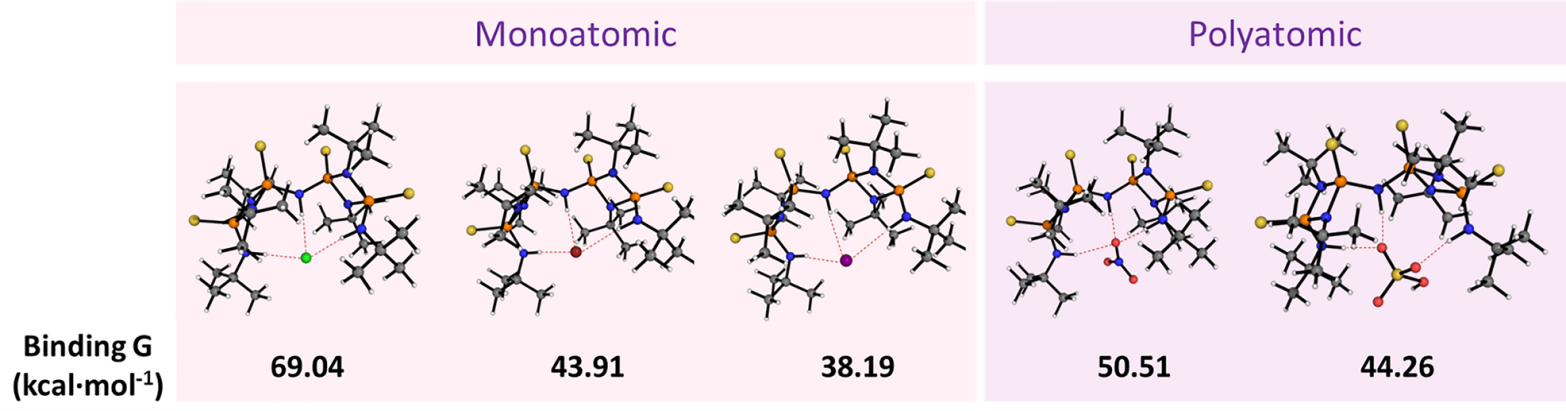
Calculated structures and binding energies for **3^ON^** ⊂ X^–^ host–guest adducts.

This is further supported by theoretical calculations of **3^ON^** and **3^OFF^** with the chloride anion (see Figure 4). In contrast to what was calculated for **3** ⊂ DMSO, the preferred binding mode is reversed for chloride anions (64.09 vs 55.02 kcal·mol^–1^ for **3^ON^** and **3^OFF^**, respectively) with a difference in stabilization of 14.02 kcal·mol^–1^ favorable to the **ON** HB motif. The **ON** bonding mode is displayed across a halide anion triad (X = Cl^–^, Br^–^, and I^–^). In all cases, the **3^ON^** ⊂ halide compounds exhibit low energies for such host–guest systems (Figure 5).

Moreover, excluding macrocycles, this type of adjacent and symmetrical HB interactions is rare^64,109,110^ and has only been previously described for C_3V_ tripodal type of frameworks—never for linear molecules—making the herein reported molecular switch unique.

Hence, we proceeded to study the **ON/OFF** host–guest properties of **3** toward anions. Compound **3** was selected over **2** due to its lower rotation barrier and its better expected performance than **2** in anion binding based on previous reports on monomeric P^V^_2_N_2_ species.^61,100^

Indeed, cocrystals of **3** obtained with anionic halides (i.e., Cl^–^, Br^–^, and I^–^) display an **ON** topological conformation with a fully engaged bis-P^V^_2_N_2_ backbone effectively utilizing all three NH moieties in HB interaction with the negatively charged halide (Figure 3B). To our knowledge, this is the first example of trifurcated R^3^_1_(8,8) HB in cyclodiphosphazane species. In addition, the **ON**/**OFF** molecular switch ability of the phosphazane host to selectively enable different HB modes to adapt to specific guests has never been reported for inorganic frameworks.

Remarkably, **3^ON^** also exhibits the ability to vary its cavity size by pivoting around the central NH moiety. This enables the compound to readily accommodate group 17 anions of different sizes, with little distortion to its framework (vide infra), which highlights its potential to respond to a broad range of differently sized anions. In contrast, such topological flexibility was not observed in its cyclic counterparts due to its rigid nature, limiting its supramolecular interactions to smaller guests.^111−114^ Hence, dimeric-P^V^_2_N_2_ species represent a promising framework with properties that are unique and complimentary to existing currently reported organic-based anion receptors and molecular switches.

### Broad Response P^V^_2_N_2_ Switch ON Mode: R^3^_1_(8,8) Trifurcated HB Anion Binding Abilities with Complex Polyatomic Anions

The ability of **3** to switch **ON** the R^3^_1_(8,8) trifurcated HB mode in response to anions showcases the potential of these species to act as high anion affinity molecular switches in supramolecular and chemically responsive architectures. To illustrate this, we titrated **3** with increasing amounts of Cl^–^. The gradual addition of tetrabutylammonium chloride (TBACl) to a solution of **3** in CDCl_3_ displayed new resonances corresponding to host–guest adduct **3^ON^** ⊂ Cl^–^, indicating negligible exchange of chloride ions between host molecules, with full conversion into **3^ON^** ⊂ Cl^–^ occurring at approximately two equivalents of TBACl. Due to these slow exchanges, the data obtained of **3^ON^** with Cl^–^ was not suitable to be fitted into a 1:1 binding isotherm model. Instead, the NH resonances were estimated using a concentration-weighted average of free host and host–guest complex, which was subsequently fitted into the 1:1 model.^115^ Using this method, the binding constant was estimated to be K_A_ = 192.92 ± 81.89 M^–1^ (Figure S31).

To demonstrate the previously proposed broad applicability of our system, larger monoatomic anions (i.e., larger halides) and polyatomic complex anions were used, namely, I^–^, HSO_4_^–^, and NO_3_^–^ (i.e., TBAI, TBAHSO_4_, and TBANO_3_, respectively). In these studies, the addition of increasing amounts of anionic species displayed a gradual downfield shift of NH resonances. This downfield shift is representative of anion binding to the NH sites present in **3^ON^** and indicative of a rapid anion exchange between molecules of **3**. The data obtained throughout the NMR titrations for each of these species were fitted into a 1:1 binding isotherm model.^115,116^ The binding constants obtained were 5.17 ± 0.13 (TBAI), 9.48 ± 0.25 (TBAHSO_4_), and 20.39 ± 1.17 M^–1^ (TBANO_3_). See Table 1.

**Table 1:**
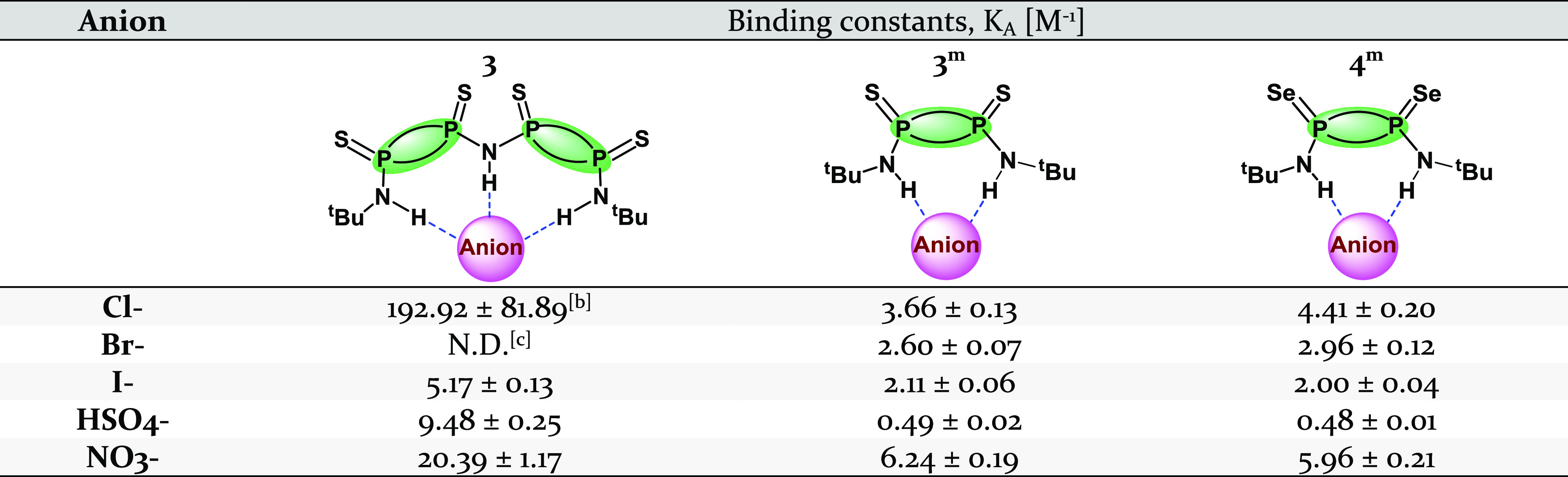
Summary of Experimental Binding Constants of 3 and its Monomeric Counterparts with Different Monoatomic and Polyatomic Anions^a^

a For theoretical calculations that assess the relative binding strengths of dimeric and monomeric P_2_^V^N_2_ species as well as urea derivatives, see Tables S8 and S9.

b Estimated based on concentration-weighted average of [H_0_] and [HG];^115^ for details, see Figure S31.

c N.D. = not determined.

Unfortunately, NMR titrations of **3** with Br^–^ displayed extensive broadening of the NH resonances of the **3^ON^** ⊂ Br^–^ adduct at lower concentrations, which was attributed to a guest exchange rate within NMR timescales. To corroborate this assumption, NMR spectroscopy of **3^ON^** with 0.5 equivalents of TBABr was conducted in CDCl_3_ at 223 K. The low temperature ^1^H NMR spectrum shows clear NH signals at 5.87 and 8.61 ppm, resulting from a lower exchange rate at 223 K, further supporting our hypothesis (Figure S18). As a result, the data collected are unsuitable to be fitted either in the isotherm binding model or estimated via the concentration-weighted average of free host and host–guest complex; thus, the binding affinity of **3^ON^** to Br^–^ was not determined.

To assess the relative binding strength of **3** to Br^–^ with respect to the other anions studied, a series of competitive binding studies involving **3** in the presence of two different anions were conducted using ESI-MS operated in negative mode (see Figures S20–29). From our results, a clear preference toward smaller halides like Cl^–^ and Br^–^ was observed. The binding of **3** toward the anions tested displays a relative binding strength of Cl^–^ > Br^–^ > I^–^ ≈ NO_3_^–^ ≈ HSO_4_^–^.

The distinct interaction of **3^ON^** with different halides is also observed in the solid state. The solid-state structures of **3^ON^** ⊂ Cl^–^, **3^ON^** ⊂ Br^–^, and **3^ON^** ⊂ I^–^ display an increasing N···X^–^ distance with increased halide size, illustrating a cavity capable of adapting to different-sized hosts while still showing good guest binding affinities. Notably, compound **3** exhibits at least twofold higher binding affinities to the anions studied (ca. 5–192 M^–1^) when compared to monomeric P^V^_2_N_2_ species (ca. 0.5–6 M^–1^; see Table 1), with binding affinity toward Cl^–^ approximately 40 times that of **4^m^**. This can be attributed to both the increased number of HB donors and larger cavity present in **3**, which can accommodate larger anions (i.e., I^–^, HSO_4_^–^, and NO_3_^–^). The cavity displays a steady increase of the terminal N···N distance on descending the group: **3^ON^** ⊂ Cl^–^ (6.272 Å) < **3^ON^** ⊂ Br^–^ (6.410 Å) < **3**^ON^ ⊂ I^–^ (6.611 Å). Such a feature is also reminiscent of the macrocyclic pentamer [{P(μ-N^t^Bu)}_2_(NH)]_5_, distorting its planar structure to host larger halides.^114^ However, in contrast to **3**, this macrocycle is not known to accommodate larger complex anions (i.e., HSO_4_^–^ or NO_3_^–^), likely due to the sterically encumbered and rigid nature of its cavity. In addition, simple *ortho*-phenylenediamine bridged bis-ureas and bis-thioureas have been demonstrated to exhibit low binding affinity toward Br^–^ anion, whereas **3** displays good binding to Br^–^ as evident through experimental results and solid-state structures.^86^

**Figure 6:**
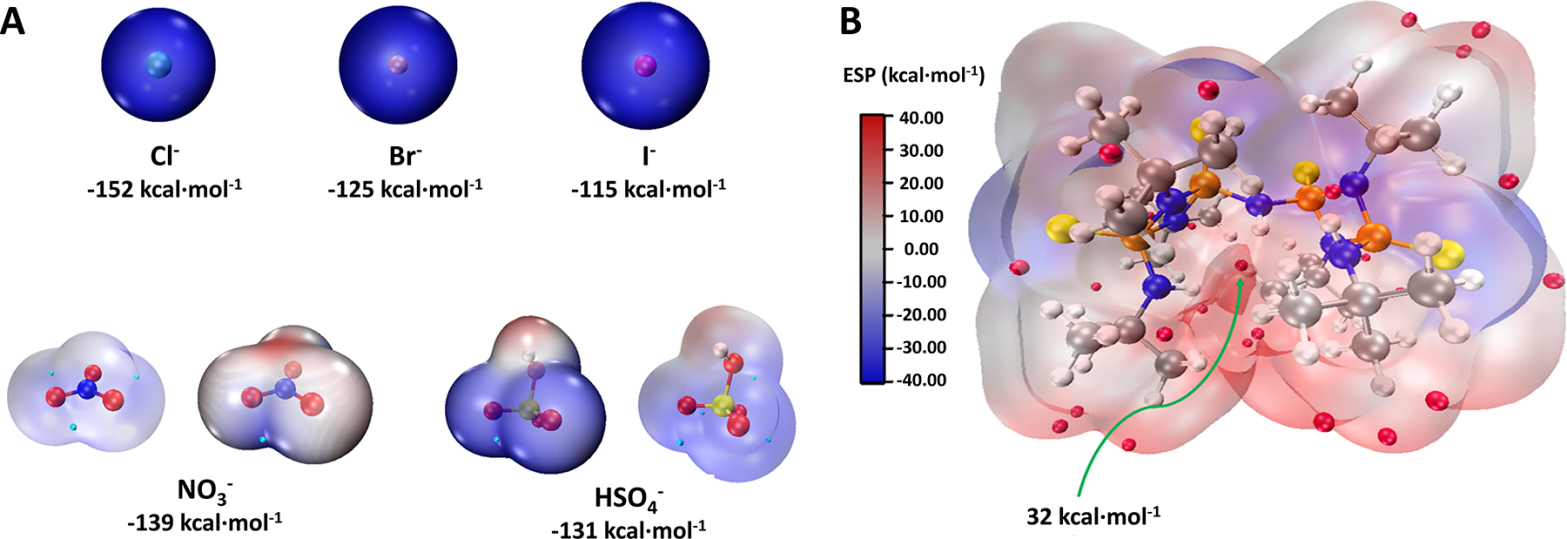
ESP surfaces of various mono- and polyatomic anions (A) and compound **3** (B) plotted on the electronic density with isovalue = 0.01.

Theoretical binding energies calculated at the B3pw91/6-311 g(d,p) theory level for **3^ON^** ⊂ X^–^ were consistent with the experimental binding trend, alongside displaying higher binding strengths when compared to monomeric P^V^_2_N_2_ species, as well as monomeric urea derivatives (Tables S8 and S9). The trend obtained is slightly overestimated due to the absence of counterions to reduce computational load (Figure 5). Similarly, the observed experimental binding trend is consistent with the electrostatic potential trend of the various mono- and polyatomic anions studied (Figure 6A). In addition, electrostatic potential (ESP) analysis of **3^ON^** displays a positive potential within the trifurcated cavity, along with negative potential around the P=S moiety (Figure 6B), indicating a polarized N–H system favoring anion complexation, consistent with our results.

### P^V^_2_N_2_ Switch ON/OFF Reversibility: R^2^_1_(8) Bifurcated ↔ R^3^_1_(8,8) Trifurcated Transitions

Given the **OFF**/**ON** modes observed, we hypothesize that these systems are topologically responsive and can regain their original topology once the chemical stimulus is withdrawn. This would thus provide the first example of a fully reversible molecular switch in the main group arena. Due to the distinct differences between **2^OFF^** and **2^ON^** observed throughout our studies, the reversibility of bifurcated and trifurcated transitions was probed using **2** as a model.

**Figure 7:**
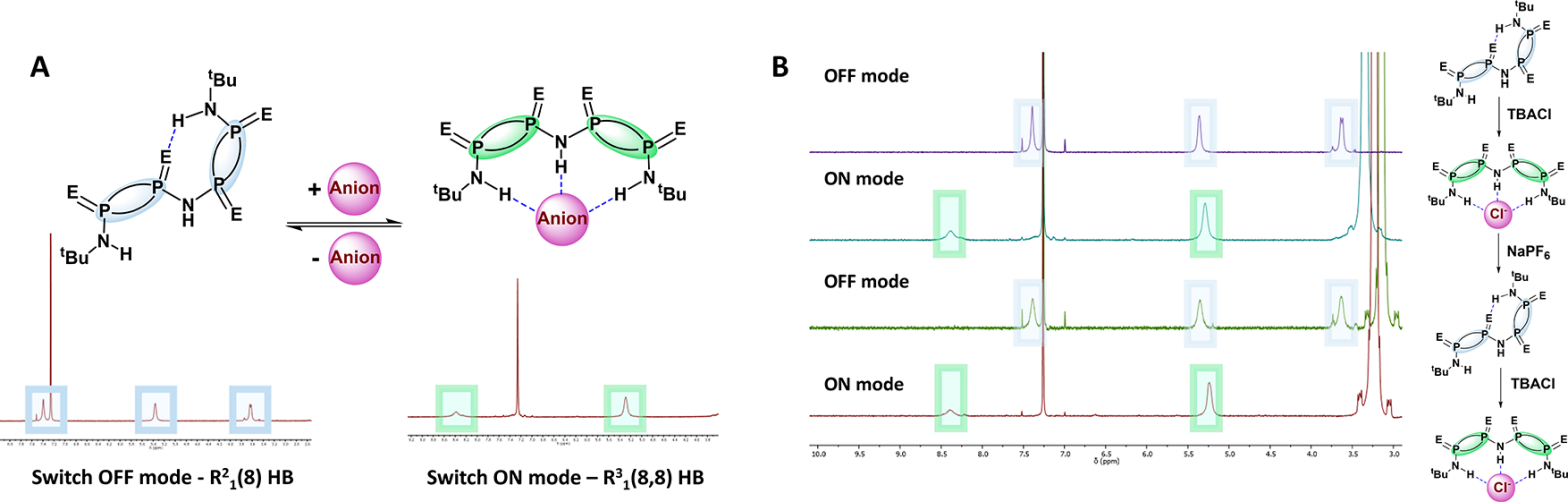
Molecular switch reversibility studies. (A) Characteristic signals corresponding to **2^ON^** and **2^OFF^**. (B) Partial ^1^H NMR spectra displaying two cycles of the reversible **ON**/**OFF** switching modes.

However, full reversibility can only be fully demonstrated when the host is able to return to its initial topological state upon removal of the chemical stimulus. For this purpose, an excess of five equivalents of TBACl was added to a solution of **2** in CDCl_3_. As expected, the original NH resonances transform into two signals at 5.29 and 8.38 ppm, indicating a halide-induced **OFF** to **ON** topological transformation (Figure 7A; see Figure S19 for more details). To prove reversibility, the halide anion guest was removed from the **2^ON^** host. The addition of five equivalents of NaPF_6_ to this solution resulted in an anion exchange, which is accompanied by the precipitation of insoluble NaCl as byproduct and the return to the **2^OFF^** topological conformation. The **OFF** topology was confirmed by in situ ^1^H NMR of the mixture, which was illustrated by the presence of the three characteristic −NH signals at approximately the same chemical shifts (Figure 7B). Further addition of five equivalents of TBACl results in **2^ON^** topology again, thus demonstrating the reversibility of the main group molecular switch.

## Conclusions

A novel molecular chemically responsive switch based on a fully inorganic backbone (i.e., carbon-free backbone) has been demonstrated for the first time. The reported NH-bridged acyclic dimeric cyclodiphosphazane molecular switches, [(μ-NH){PE(μ-N^t^Bu)_2_PE(NH^t^Bu)}_2_] (E = O and S), display an anion-responsive bimodal bifurcated R^2^_1_(8) to trifurcated R^3^_1_(8,8) hydrogen bonding transitions.

In contrast to conventional organic frameworks where carbon atoms display fixed valency and oxidation states, the reported parent P^III^N backbone readily gives rise to two different P^V^N species with distinct switching energy barriers (i.e., O vs S derivatives) via a single synthetic backbone modification step, which is enabled by the readily accessible variable oxidation states.

In addition, the reported species display a higher affinity toward anion species than their monomeric counterparts (see Supporting Information)—previously described in the literature as excellent alternatives to squaramides and thioureas—with a topologically responsive and adaptable cavity size.

Finally, our work serves as a proof of concept to highlight main group frameworks as powerful chemically responsive switches. Apart from being excellent anion receptors/sensors, we believe that such frameworks would give rise to potential applications such as anion-activated molecular tweezers or anion-responsive materials and polymers, when implemented as building blocks in combination with existing well-established systems. Therefore, we envision main group elements playing a key role in supramolecular chemistry through the design of carbon-free and hybrid (i.e., organic/inorganic) molecular machinery and host–guest systems in the near future.

## Supporting Information Available

Additional synthetic methods, nuclear magnetic resonance spectra, single-crystal X-ray diffraction data, and computational details (PDF)
XYZ files for theoretical calculations (ZIP)

## Dedication

In memory of Professor Mary McPartlin.
