# Supplementary material for: Main Group Molecular Switches with Swivel Bifurcated to Trifurcated Hydrogen Bond Mode of Action
Source: J Am Chem Soc. 2023 Jun 2;145(23):12475–86. doi: 10.1021/jacs.2c12713 (PMC10273318; doi:10.1021/jacs.2c12713)
Supplement: Supplementary file 1 — ja2c12713_si_001.pdf [file ja2c12713_si_001.pdf]

Main Group Molecular Switches with Swivel Bifurcated to Trifurcated Hydrogen Bond Mode of Action

Gavin Hum,[a,‡] Si Jia Isabel Phang,[a,‡] How Chee Ong,[a] Felix León,[a] Shina Quek,[a] Yi Xin Joycelyn Khoo,[a] Chenfei Li,[a] Yongxin Li,[a] Jack K. Clegg,[b] Jesús Díaz,[c] Mihaiela C. Stuparu,[a] and Felipe García[d,e*]

a School of Chemistry, Chemical Engineering & Biotechnology, Nanyang Technological University, 21 Nanyang Link 637371, Singapore (Singapore)

b School of Chemistry and Molecular Biosciences, Cooper Road, The University of Queensland, St Lucia, 4072, QLD, Australia

c Departamento de Química Orgánica e Inorgánica, Facultad de Veterinaria Extremadura, Avda de la Universidad s/n, 10003, Spain.

d Departamento de Química Orgánica e Inorgánica, Facultad de Química, Universidad de Oviedo, Julián Claveria 8, Oviedo 33006, Asturias, Spain

e. School of Chemistry, Monash University, Clayton, Victoria 3800, Australia.

Table of Contents

1. Experimental Section

2. NMR Spectra

3. Topological Rearrangement and Competitive Binding Experiments

4. Binding Studies

5. X-ray analyses

6. Theoretical studies

8. References

1. Experimental Section

Compounds **1**, **2**, **3**, **3m**, **4**, **4m** were prepared under dry, O_2-free Ar atmosphere on a double manifold (argon/vacuum) line. All solvents (toluene, THF, n-hexane, diethyl ether, n-pentane) were freshly distilled over appropriate drying agents (sodium/benzophenone) under nitrogen atmosphere, degassed and stored under molecular sieves. Starting materials were either synthesised as described below or obtained commercially from Strem, Sigma-Aldrich, Alfa-Aesar and used without further purification unless otherwise stated. Et_3N was distilled from calcium hydride before use and stored under argon and molecular sieves. Starting material Cl[P(μ-N^{t}Bu)]$_2$NH^{t}Bu,[1] **3m**[2] and **4m**[3] were synthesized according to reported procedures. Compounds **1**, **2**, **3**, **3m**, **4a**, **4m** were isolated and characterised with the aid of an Ar-filled Innovative Technology glove box. ^{1}H, ^{13}C and ^{31}P{^{1}H} NMR spectra were recorded on Bruker BBFO 400 MHz spectrometer in the appropriate deuterated solvent (using the solvents resonances as the internal standard for ^{1}H and ^{13}C NMR and 85% H_3PO_4 – D_2O as the external standard for ^{31}P NMR). In situ ^{31}P{^{1}H} NMR spectroscopic studies on reaction mixtures in non-deuterated solvents were recorded using an internal acetone-d$_6$ capillary to obtain a lock. Single crystal x-ray diffraction studies were carried out with Bruker X8 CCD diffractometer.

1.1 Synthesis of [(μ-NH){P(μ-N^{t}Bu)$_2$P(NH^{t}Bu)}$_2$] (1)

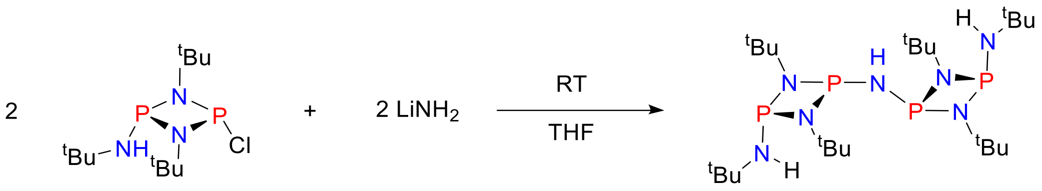

Scheme S1

A suspension of Cl[P(μ-N^{t}Bu)]$_2$NH^{t}Bu (5.00 g, 16.0 mmol) and $LiNH_2$ (0.39 g, 17.0 mmol) in dry THF (60 mL) was stirred at room temperature for 3 hours. The solvent was removed under vacuum and the white residue obtained was extracted with dry hexanes (40 mL) and filtered through celite (P3). The residue was washed with hexanes (3 x 5 mL) and the solvent was removed under vacuum. The obtained crude product was dissolved in minimal amounts of hot toluene and storage at -20°C for 12 hours yielded colourless crystals. The crystals were isolated after washing with cold n-pentane (1.5 mL). Isolated Yield: 1.02 g, 1.80 mmol (22%).

^{1}H NMR (C_6D_6, 400 MHz): δ 4.26 (t, *J* = 7.3 Hz, 1H), 3.30 (d, *J* = 6.9 Hz, 2H), 1.49 (s, 36H), 1.21 (s, 18H); ^{13}C{^{1}H} NMR (C_6D_6, 101 MHz): δ 52.49 (dt, *J* = 15.1, 7.6 Hz), 51.29 (d, *J* = 13.5 Hz), 32.86 (d, *J* = 9.5 Hz), 31.13 (dt, *J* = 7.9, 4.0 Hz); ^{31}P{^{1}H} NMR (C_6D_6, 162 MHz): δ 104.62 (s), 100.90 (s); HRMS (ESI) m/z for $C_{24}H_{58}N_7P_4$ [M+H]$^+$, Calculated: 568.3704 Found: 568.3694.

1.2 Synthesis of [(μ-NH){PO(μ-N^{t}Bu)$_2$PO(NH^{t}Bu)}$_2$] (2)

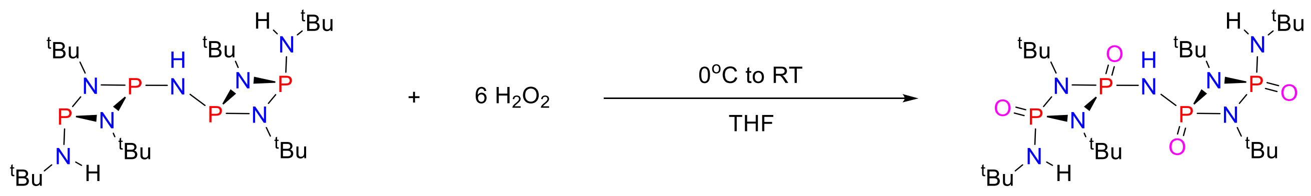

Scheme S2

A solution of 30% H_2O_2 in water (324 μL, 3.17 mmol) was added dropwise to a solution of [(μ-NH){P(μ-N^{t}Bu)$_2$P(NH^{t}Bu)}$_2$] (300 mg, 0.528 mmol) in dry THF (15 mL) at 0°C and stirred for 1 hour. The reaction mixture was allowed to warm up to room temperature and stirred for an additional 24 hours. The reaction mixture was then dried over $MgSO_4$ and filtered. The solvent was removed under vacuum and the white solid was recrystallised in minimal

amounts of THF to yield colourless crystals. Isolated Yield: 102 mg, 0.161 mmol (30%). X-ray quality crystals were grown from slow evaporation of a saturated chloroform or THF solution.

^{1}H NMR ($CDCl_3$, 400 MHz): δ 7.39 (s, 1H), 5.36 (s, 1H), 3.63 (d, *J* = 10.2 Hz, 1H), 1.55 (s, 36H), 1.47 (s, 9H), 1.34 (s, 9H); ^{1}H NMR (CD_3OD, 400 MHz): δ 1.55 (s, 36H), 1.38 (s, 18H);^{13}C{^{1}H} NMR (CD_3OD, 101 MHz): δ 57.36 (s), 53.89 (s), 32.29 (d, *J* = 4.8 Hz), 31.27 (t, *J* = 4.6 Hz); ^{31}P{^{1}H} NMR ($CDCl_3$, 162 MHz): δ -1.11 (br), -6.66 (br); ^{31}P{^{1}H} NMR (CD_3OD, 162 MHz): δ -0.68 (d, *J* = 57.3 Hz), -5.76 (d, *J* = 56.9 Hz); HRMS (ESI) m/z for $C_{24}H_{58}N_7O_4P_4$ $[M+H]^+$, Calculated: 632.3501 Found: 632.3497

1.3 Synthesis of [(μ-NH){PS(μ-N^{t}Bu)$_2$PS(NH^{t}Bu)}$_2$] (3)

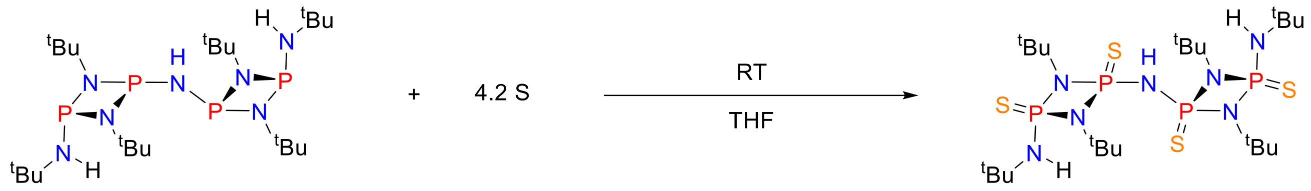

Scheme S3

A suspension of [(μ-NH){P(μ-N^{t}Bu)$_2$P(NH^{t}Bu)}$_2$] (300 mg, 0.528 mmol) and elemental sulfur (71.2 mg, 2.22 mmol) in dry THF (12 mL) was stirred at room temperature for 12 hours. The reaction mixture was filtered, and the solvent was removed under vacuum. Methanol was added to the crude product and the solution was filtered to remove the unreacted elemental sulfur. The solvent was removed under vacuum and the pale-yellow solid was recrystallised in minimal amounts of methanol to yield colourless crystals. Isolated Yield: 118 mg, 0.170 mmol (32%).

Co-crystals and solvates of **3**.

3⊂Acetonitrile: X-ray quality crystals were grown from slow evaporation of a saturated acetonitrile solution.
3⊂DMSO: X-ray quality crystals were grown from slow evaporation of a saturated dichloromethane solution with a few drops of DMSO.
3⊂Cl^-: X-ray quality crystals were grown from slow evaporation of a 1:1 **3**/TBACl in chloroform solution.
3⊂Br^-: X-ray quality crystals were grown from a 1:1 **3**/TBABr in chloroform solution layered with pentane.
3⊂I^-: X-ray quality crystals were grown from a 1:1 **3**/TBAI in chloroform solution layered with pentane.
3⊂NO_3^-: X-ray quality crystals were grown from a 1:1 **3**/$TBANO_3$ in chloroform solution layered with pentane.

^{1}H NMR ($CDCl_3$, 400 MHz): δ 5.16 (br, 1H), 4.12 (br, 2H), 1.72 (s, 36H), 1.44 (s, 18H); ^{13}C{^{1}H} NMR ($CDCl_3$, 101 MHz): δ 58.61 (s), 55.23 (d, *J* = 4.1 Hz), 31.58 (d, *J* = 4.5 Hz), 30.06 (dt, *J* = 4.6, 2.3 Hz); ^{31}P{^{1}H} NMR ($CDCl_3$, 162 MHz): δ 41.93 (d, *J* = 29.8 Hz), 34.28 (s); HRMS (ESI) m/z for $C_{24}H_{58}N_7P_4S_4$ $[M+H]^+$, Calculated: 696.2587 Found: 696.2588

1.4 Oxidation of [(μ-NH){P(μ-N^{t}Bu)$_2$P(NH^{t}Bu)}$_2$] employing elemental selenium

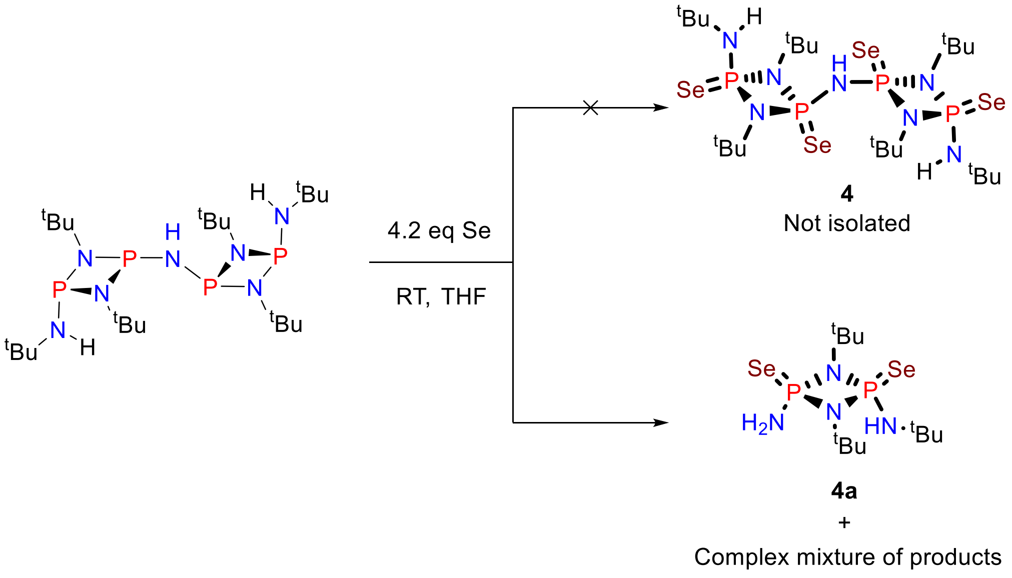

Scheme S4

A mixture of [(μ-NH){P(μ-N^{t}Bu)$_2$P(NH^{t}Bu)}$_2$] (300 mg, 0.528 mmol) and elemental selenium (175 mg, 2.22 mmol) in dry THF (12 mL) was stirred at room temperature for 12 hours. The reaction mixture was filtered, and solvent was removed under vacuum. The crude product was dissolved in minimum amounts of toluene and slow evaporation of this saturated solution yielded x-ray diffraction quality crystals. However, $^{31}P\{^1H\}$ NMR of these crystals shows multiple signals similar to that of the crude mixture, suggestive that the crystals are a mixture of species, therefore, the product was only characterised by single crystal x-ray diffraction.

2. NMR Spectra

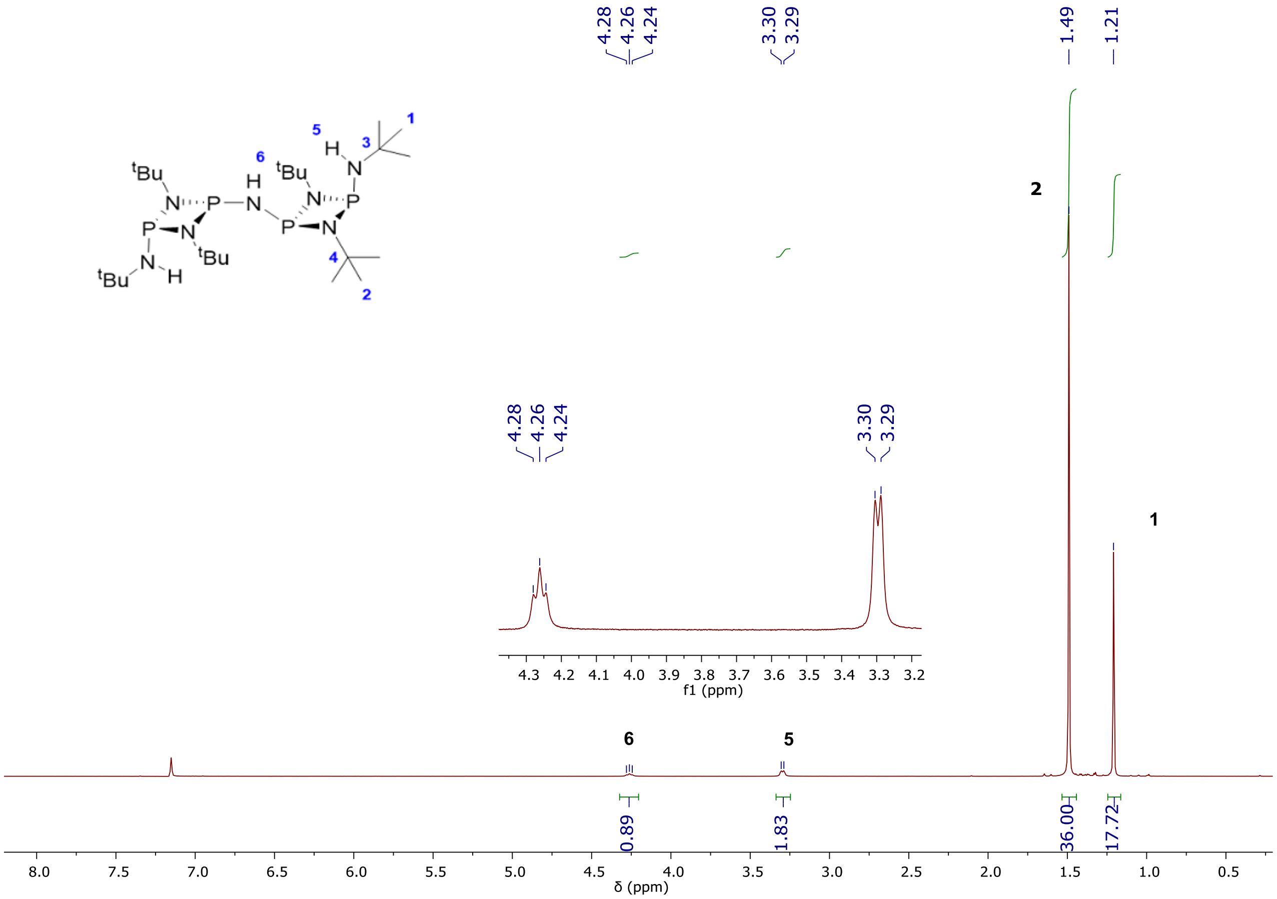

Figure S1: ^{1}H NMR spectrum of [(μ-NH){P(μ-N^{t}Bu)$_2$P(NH^{t}Bu)}$_2$] (**1**) in C_6D_6.

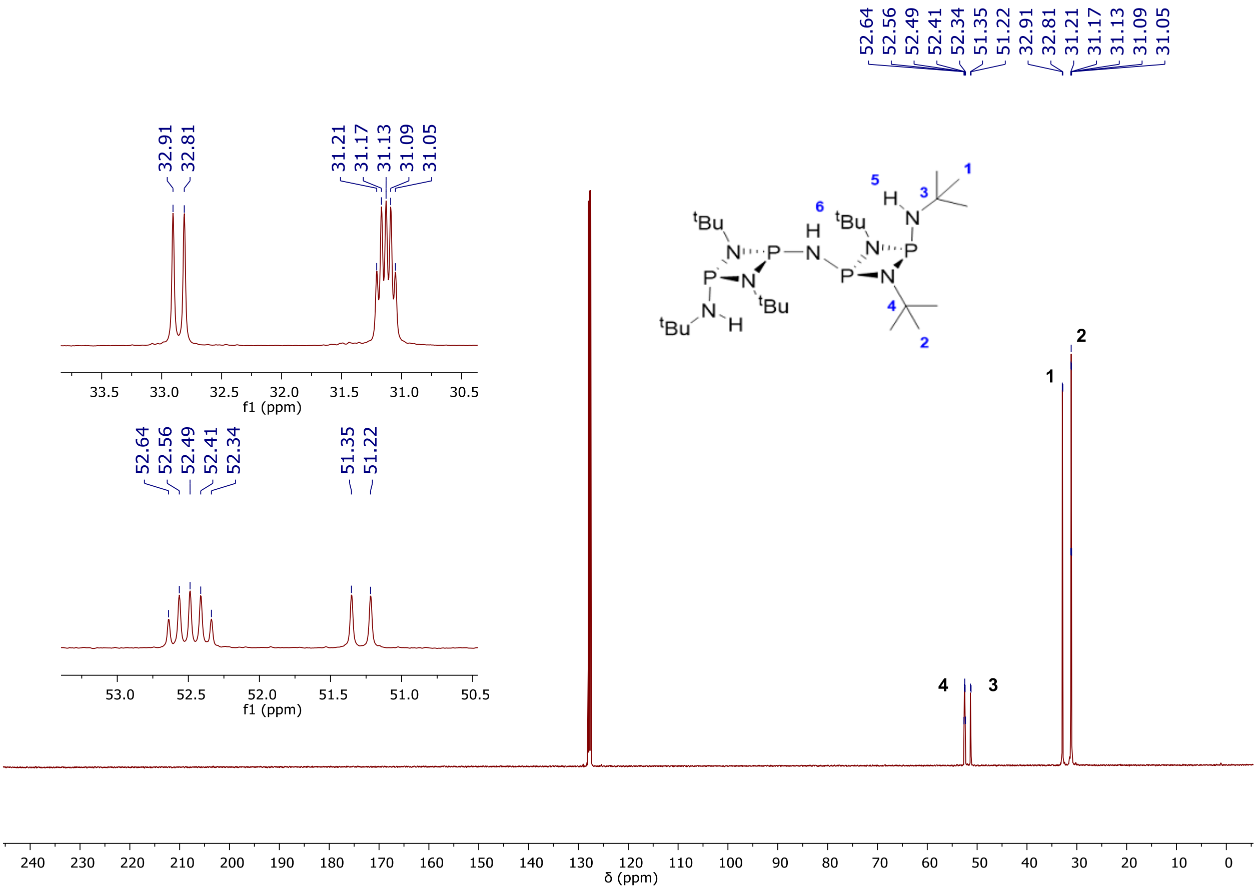

Figure S2: $^{13}C\{^{1}H\}$ NMR spectrum of [(μ-NH){P(μ-N^{t}Bu)$_2$P(NH^{t}Bu)}$_2$] (**1**) in C_6D_6.

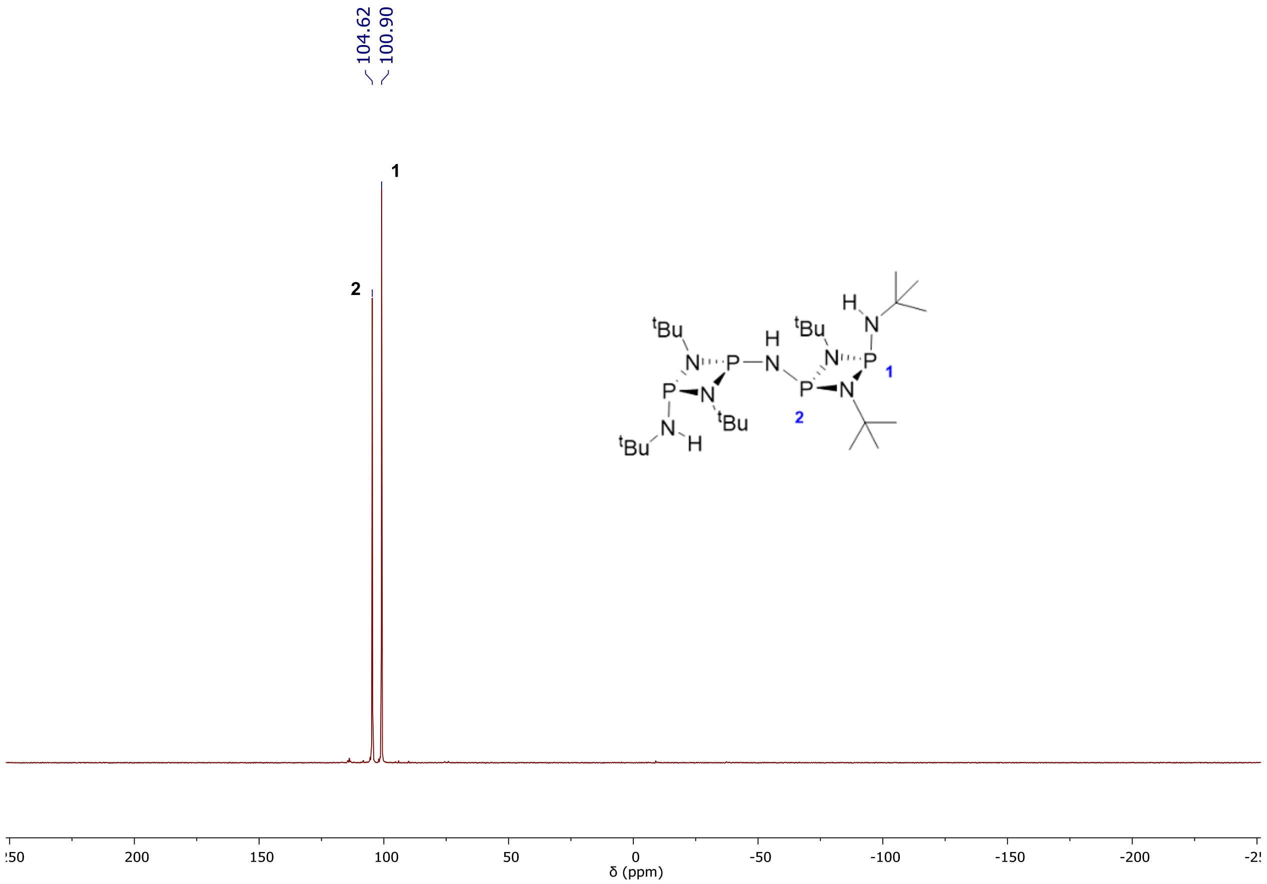

Figure S3: ^{31}P{^{1}H} NMR spectrum of [(μ-NH){P(μ-N^{t}Bu)$_2$P(NH^{t}Bu)}$_2$] (**1**) in C_6D_6.

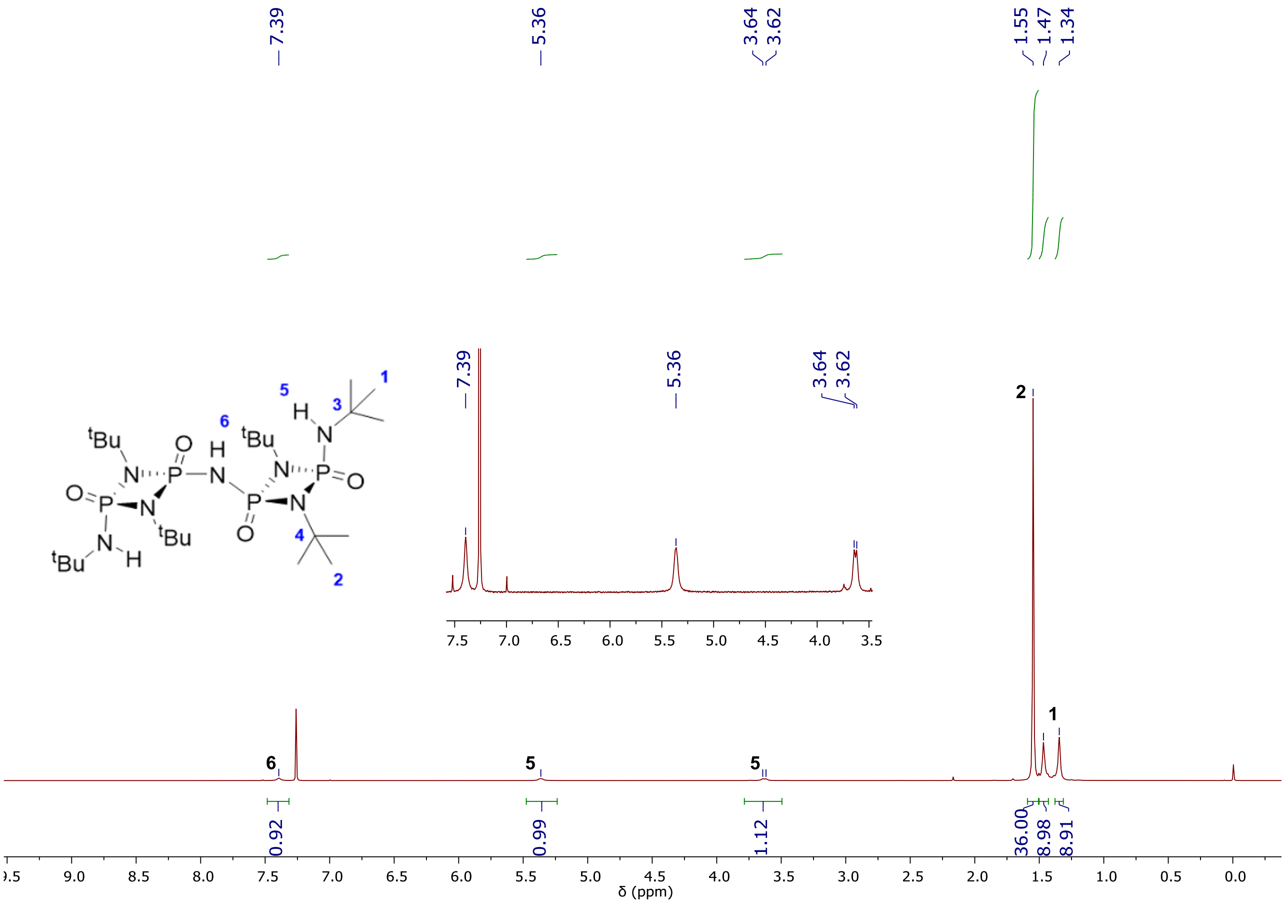

Figure S4: ^{1}H NMR spectrum of [(μ-NH){PO(μ-N^{t}Bu)$_2$PO(NH^{t}Bu)}$_2$] (**2**) in $CDCl_3$.

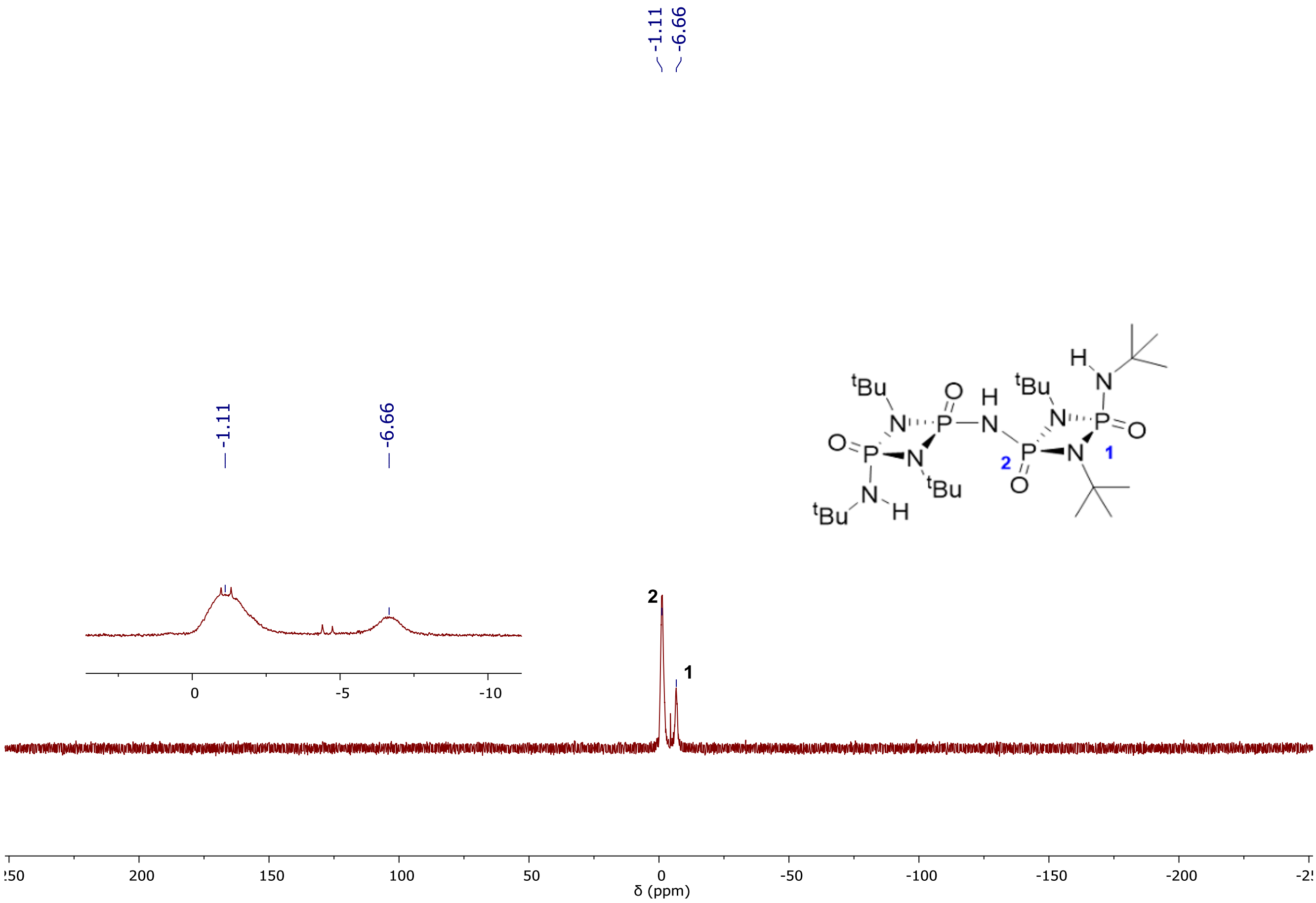

Figure S5: $^{31}P\{^1H\}$ NMR spectrum of [(μ-NH){PO(μ-N^{t}Bu)$_2$PO(NH^{t}Bu)}$_2$] (**2**) in $CDCl_3$.

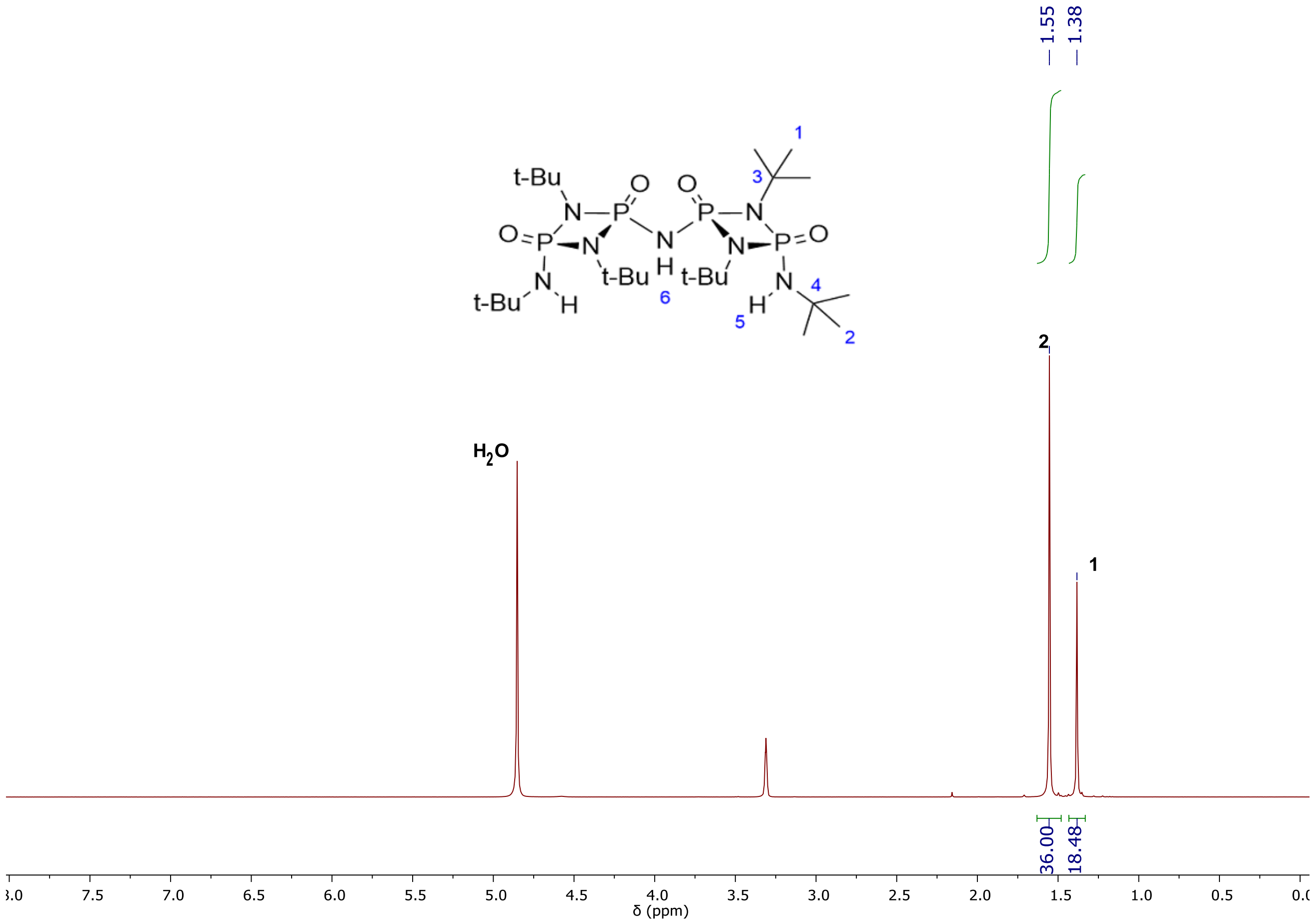

Figure S6: ^1H NMR spectrum of [(μ-NH){PO(μ-N^{t}Bu)$_2$PO(NH^{t}Bu)}$_2$] (**2**) in CD_3OD.

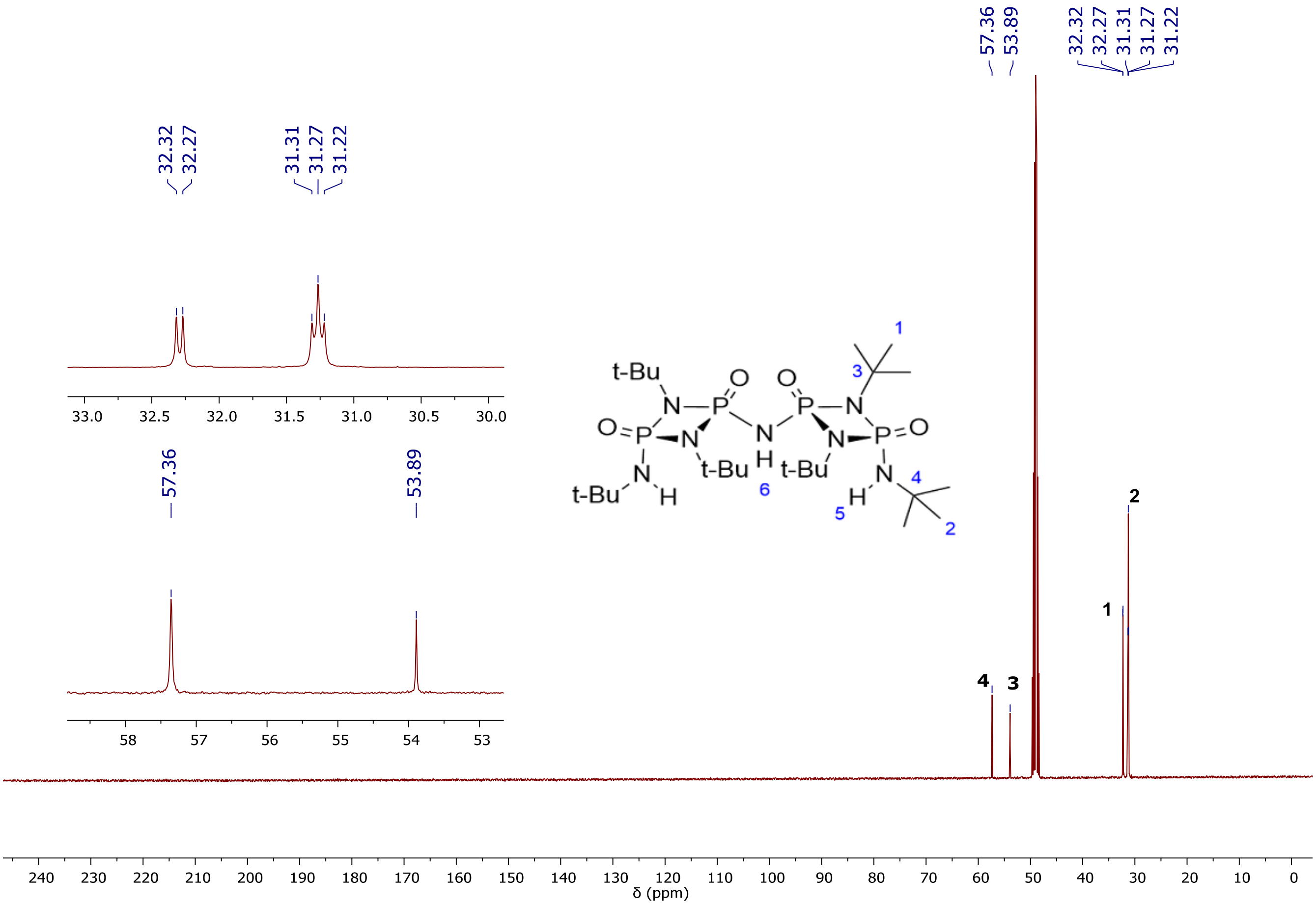

Figure S7: $^{13}C\{^{1}H\}$ NMR spectrum of [(μ-NH){PO(μ-N^{t}Bu)$_2$PO(NH^{t}Bu)}$_2$] (**2**) in CD_3OD.

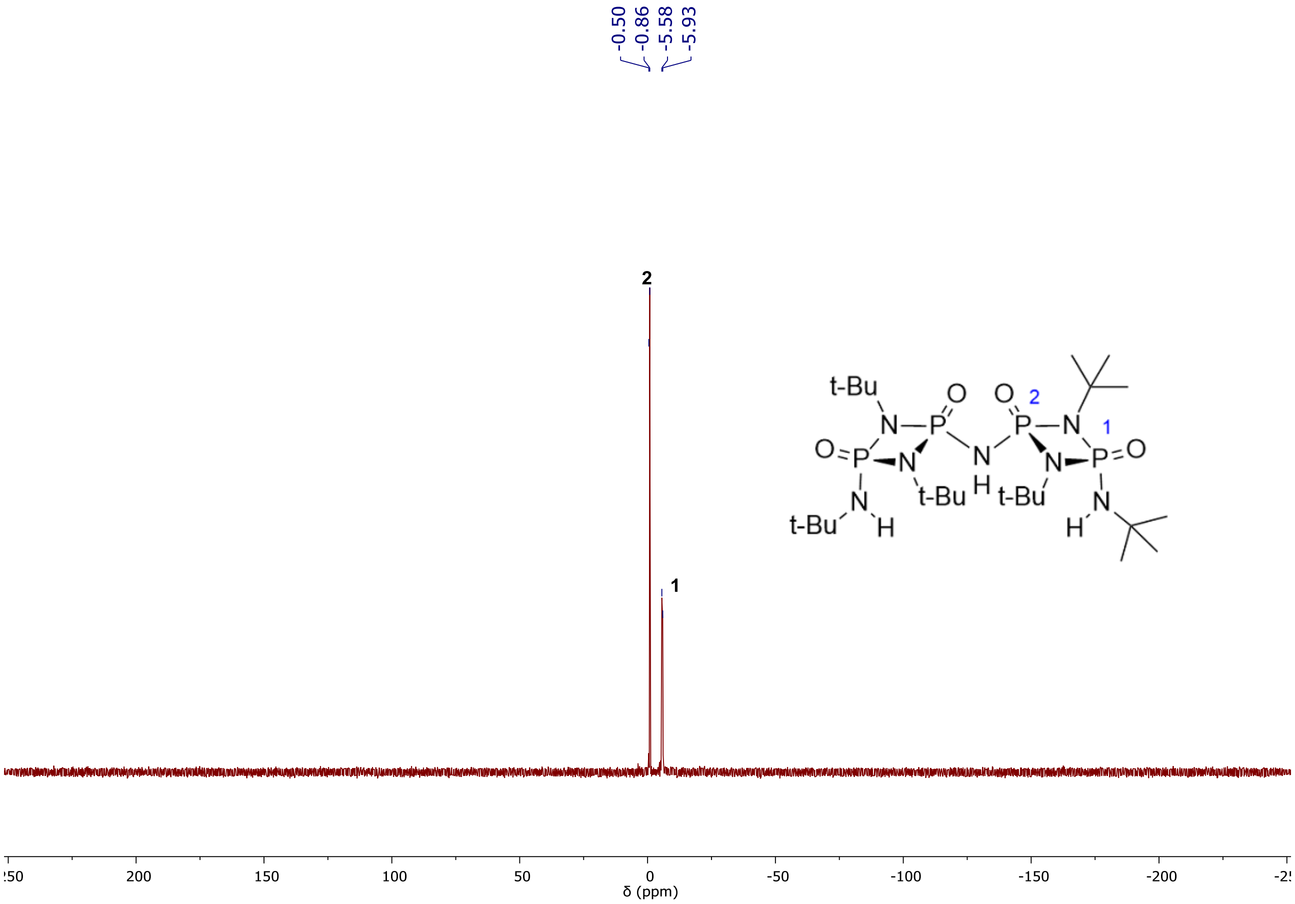

Figure S8: $^{31}P\{^{1}H\}$ NMR spectrum of [(μ-NH){PO(μ-N^{t}Bu)$_2$PO(NH^{t}Bu)}$_2$] (**2**) in CD_3OD.

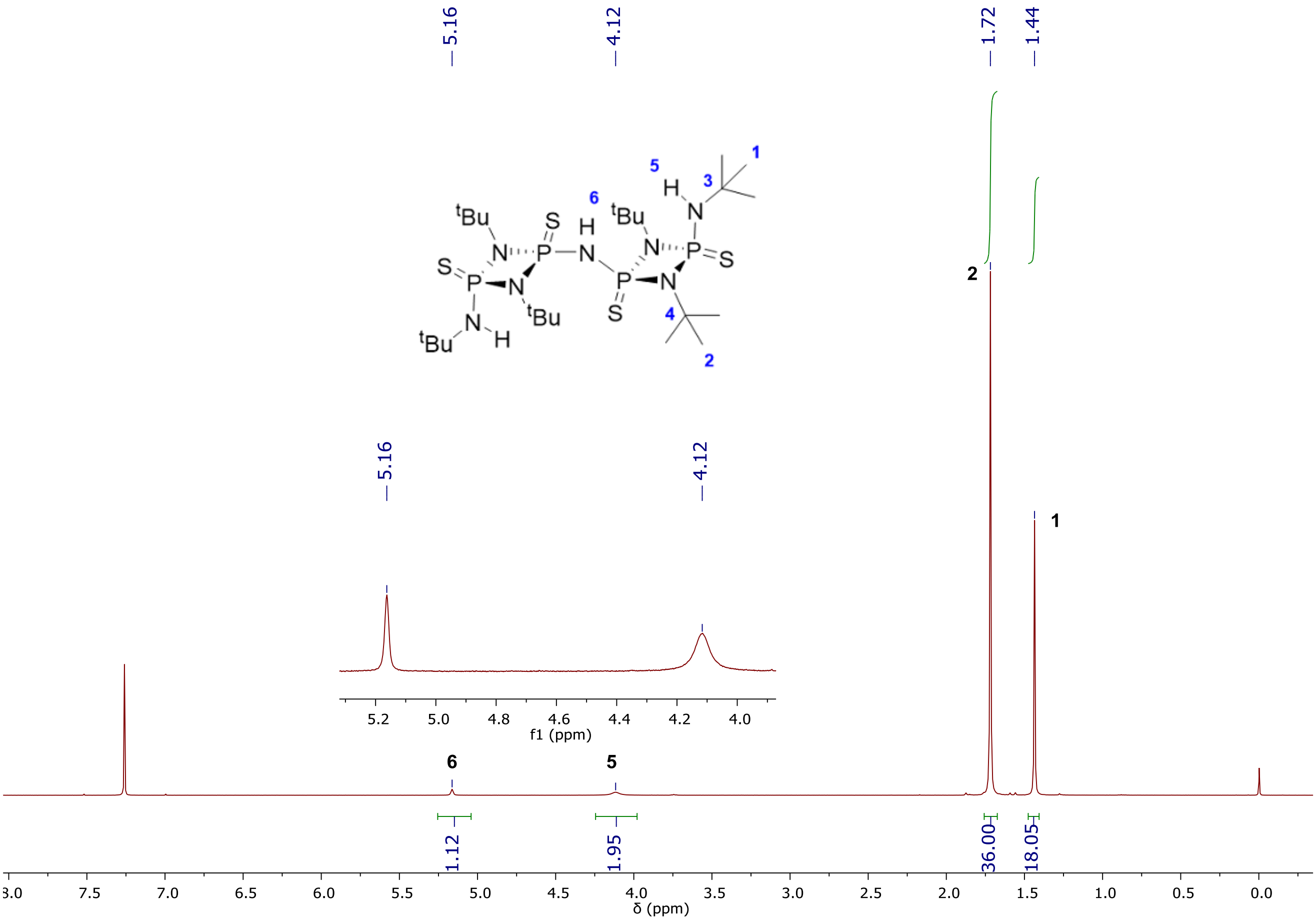

Figure S9: ^{1}H NMR spectrum of [(μ-NH){PS(μ-N^{t}Bu)$_2$PS(NH^{t}Bu)}$_2$] (**3**)in $CDCl_3$.

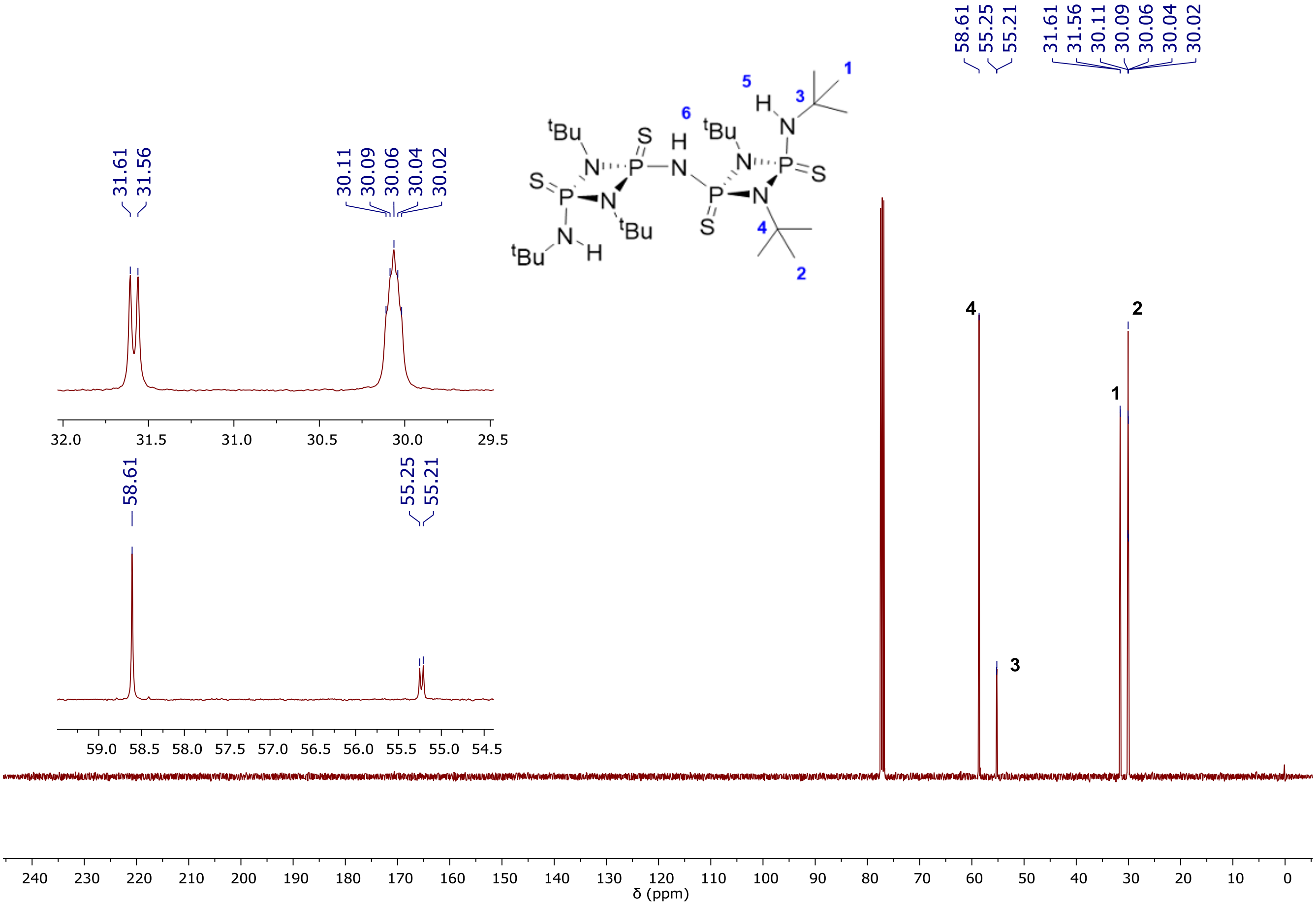

Figure S10: $^{13}C\{^{1}H\}$ NMR spectrum of [(μ-NH){PS(μ-N^{t}Bu)$_2$PS(NH^{t}Bu)}$_2$] (**3**) in $CDCl_3$.

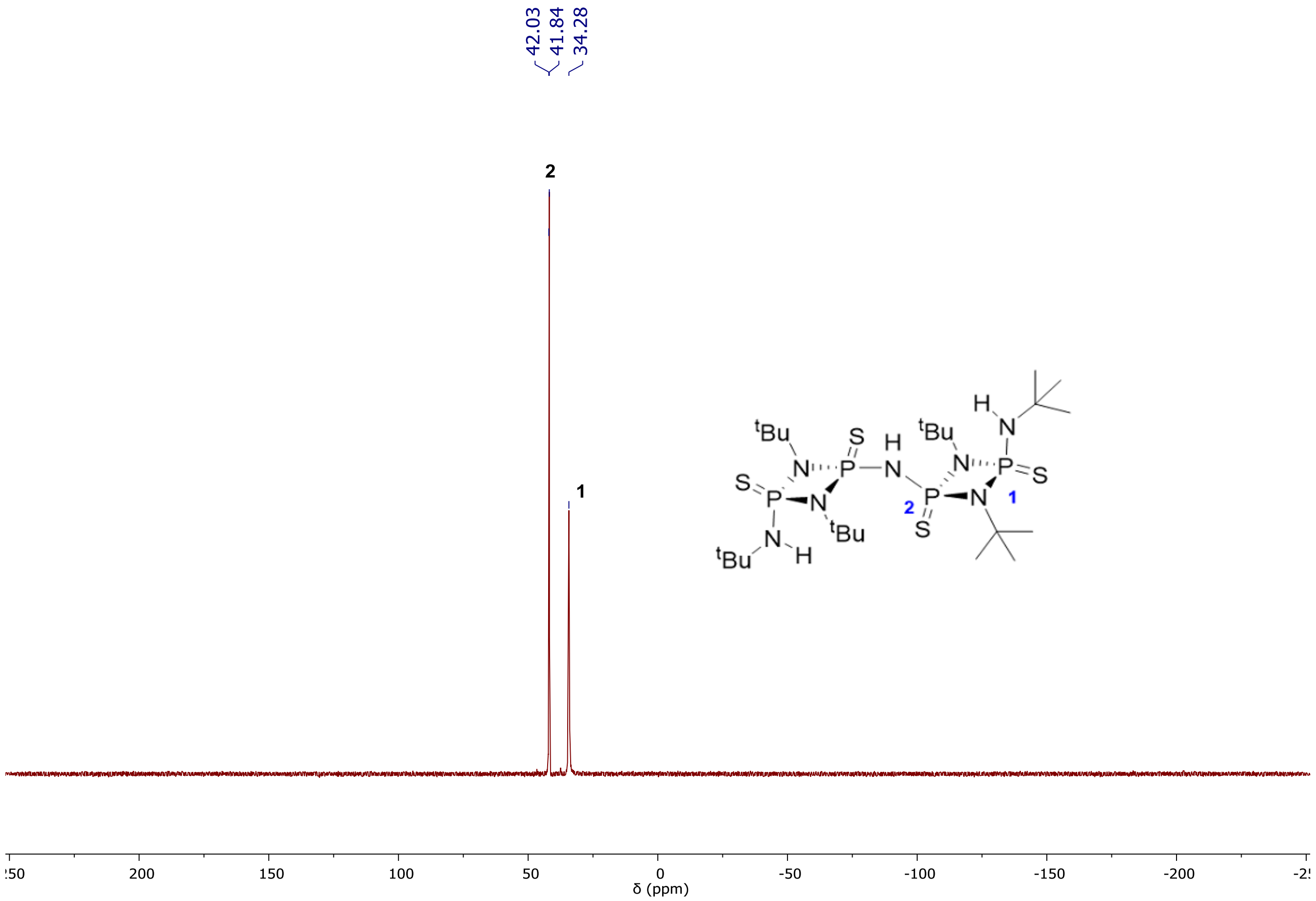

Figure S11: $^{31}P\{^{1}H\}$ NMR spectrum of [(μ-NH){PS(μ-N^{t}Bu)$_2$PS(NH^{t}Bu)}$_2$] (**3**) in $CDCl_3$.

3. Topological Rearrangement and Competitive Binding Experiments

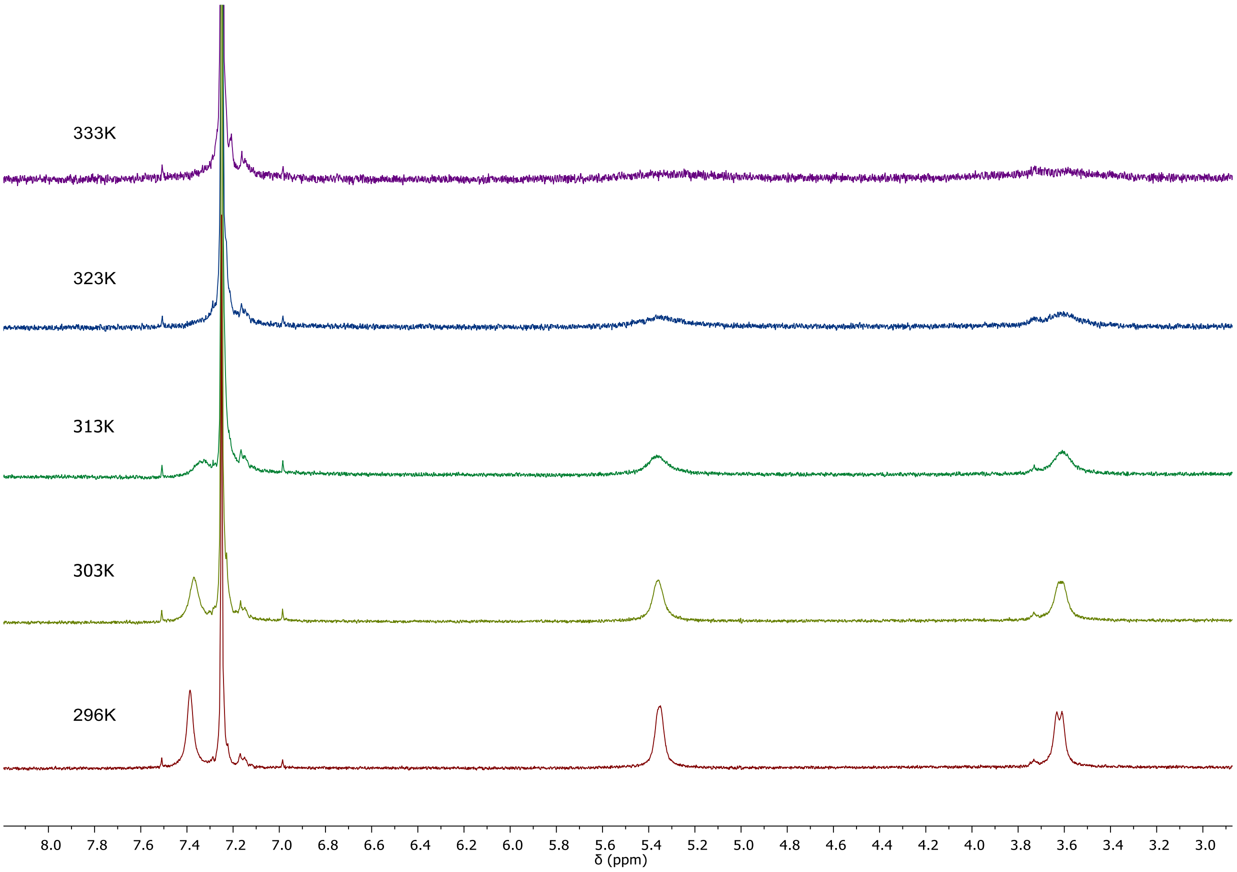

Figure S12: Variable temperature ^{1}H NMR of **2** in $CDCl_3$ at different temperatures showcasing N-H signals.

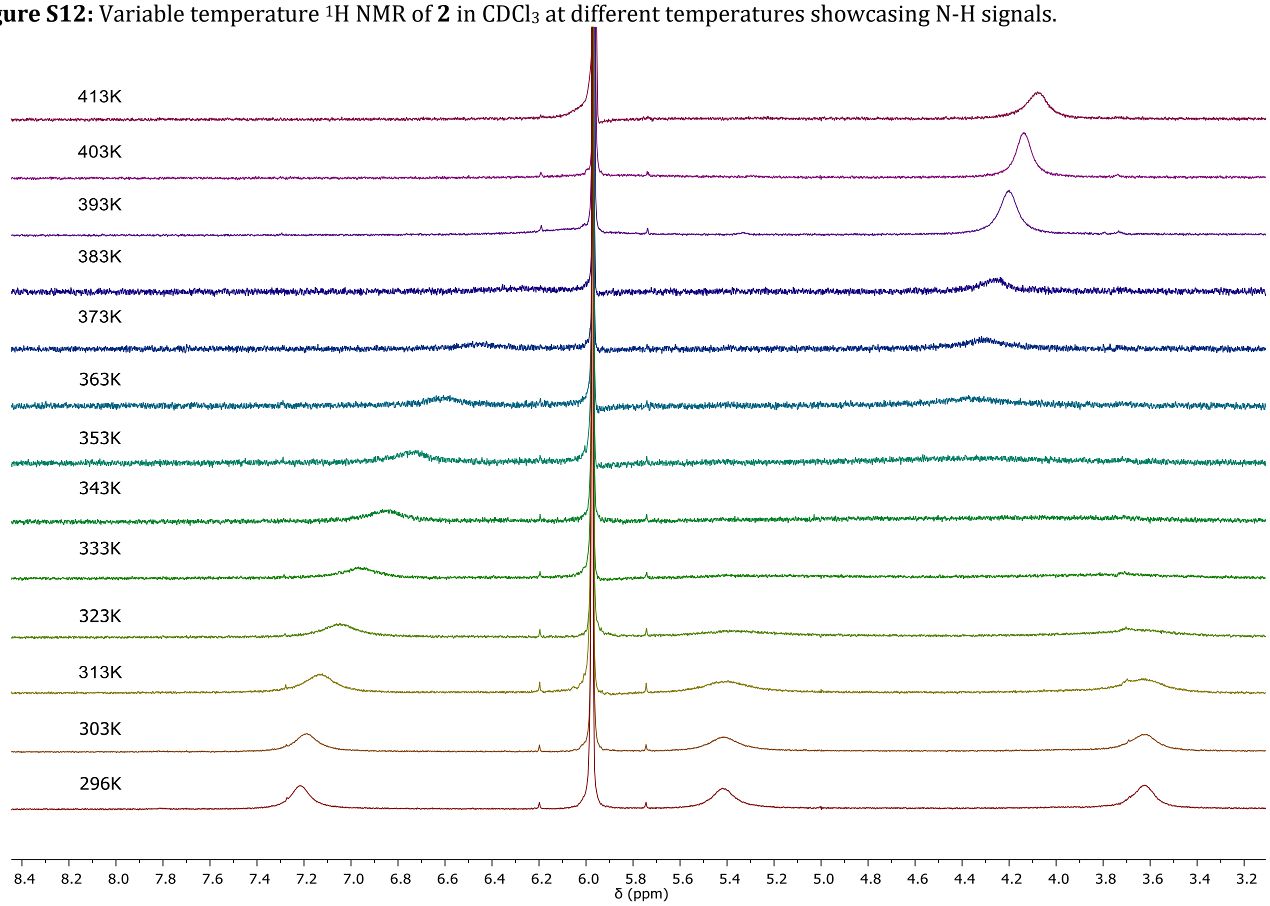

Figure S13: Variable temperature ^{1}H NMR of **2** in tetrachloroethane-d_2 at different temperatures showcasing N-H signals.

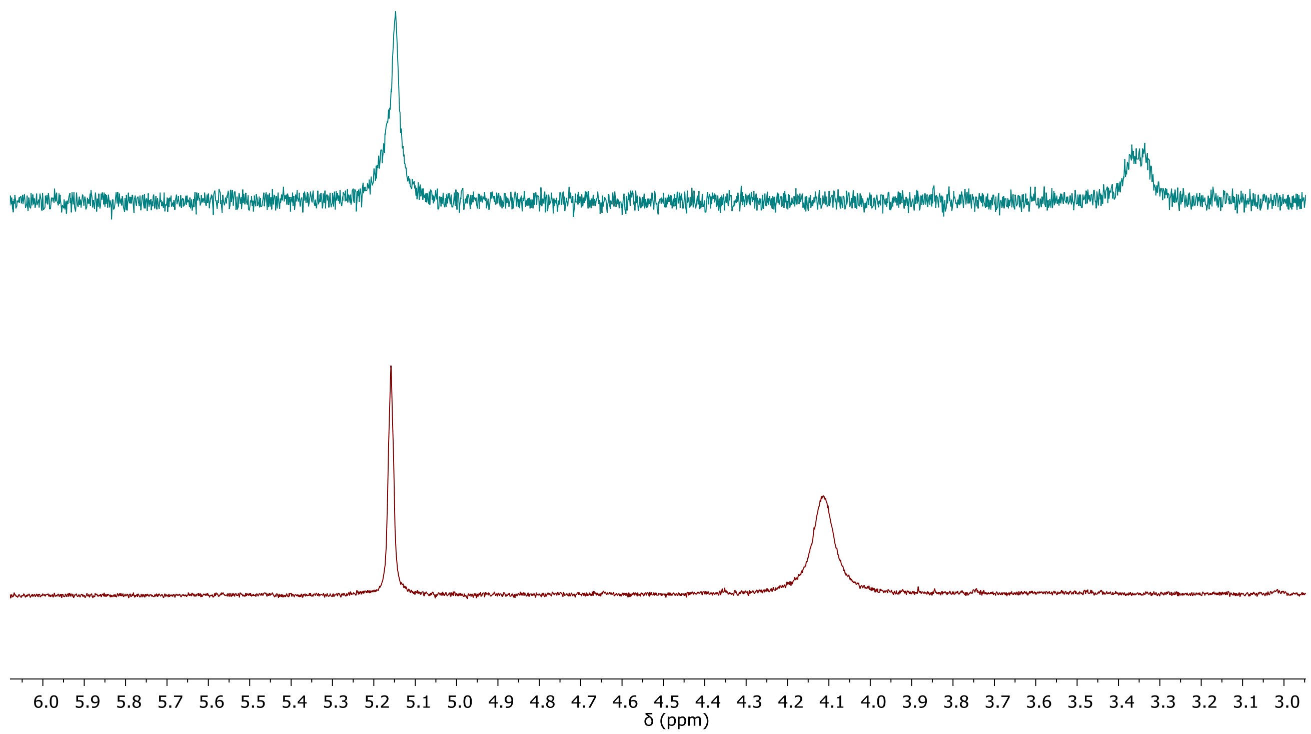

Figure S14: Overlaid ^{1}H NMR of **3** in $CDCl_3$ at room temperature (bottom) and 213 K (top).

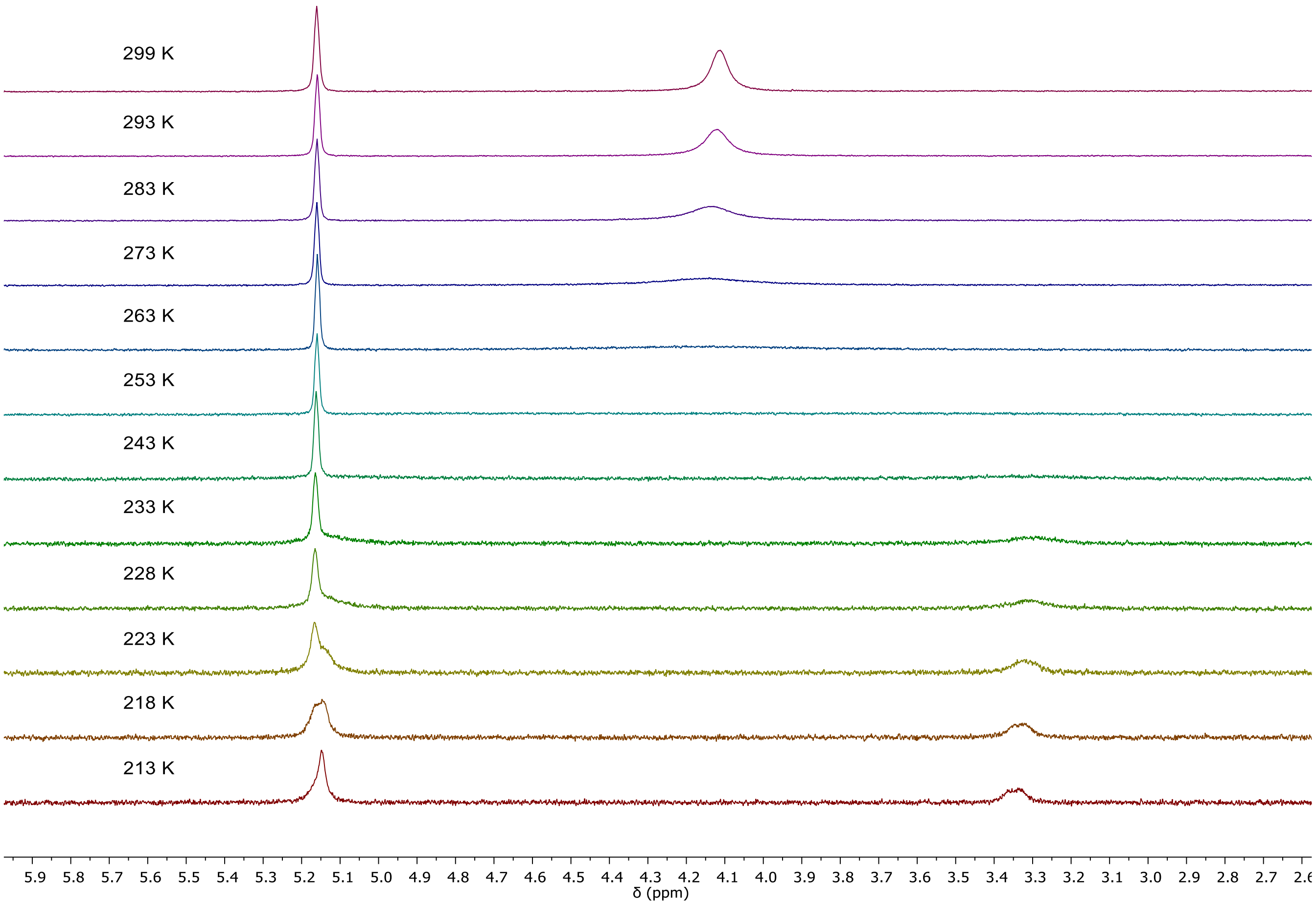

Figure S15: Variable temperature ^{1}H NMR of **3** in $CDCl_3$ at different temperatures showcasing N-H signals.

Using the data obtained from the variable temperature ^{1}H NMR spectroscopy experiments, the rotational energy barrier for the topological rearrangement for both compounds **2** and **3** between **C** and **S** conformation was estimated using the following equation derivated from the Eyring equation.[4]

$$\Delta G = RT_c\left[22.96 + ln\left(\frac{T_c}{\Delta v}\right)\right]$$

Where,

R = gas constant in $cal{\cdot}K^{-1}{\cdot}mol^{-1}$
T_c = coalescence temperature in K
$\Delta\nu$ = the difference in chemical shift ($\delta_A - \delta_B$) in Hz

Rotational energy barrier for **2** in tetrachloroethane-d_2:

Coalescence temperature = 353 K

$$\Delta G = 1.987 \times 353\left[22.96 + ln\left(\frac{353}{720}\right)\right]$$

ΔG = 15.6 $kcal{\cdot}mol^{-1}$

Rotational energy barrier for **3** in $CDCl_3$:

Coalescence temperature = 253 K

$$\Delta G = 1.987 \times 253\left[22.96 + ln\left(\frac{253}{722}\right)\right]$$

ΔG = 11.0 $kcal{\cdot}mol^{-1}$

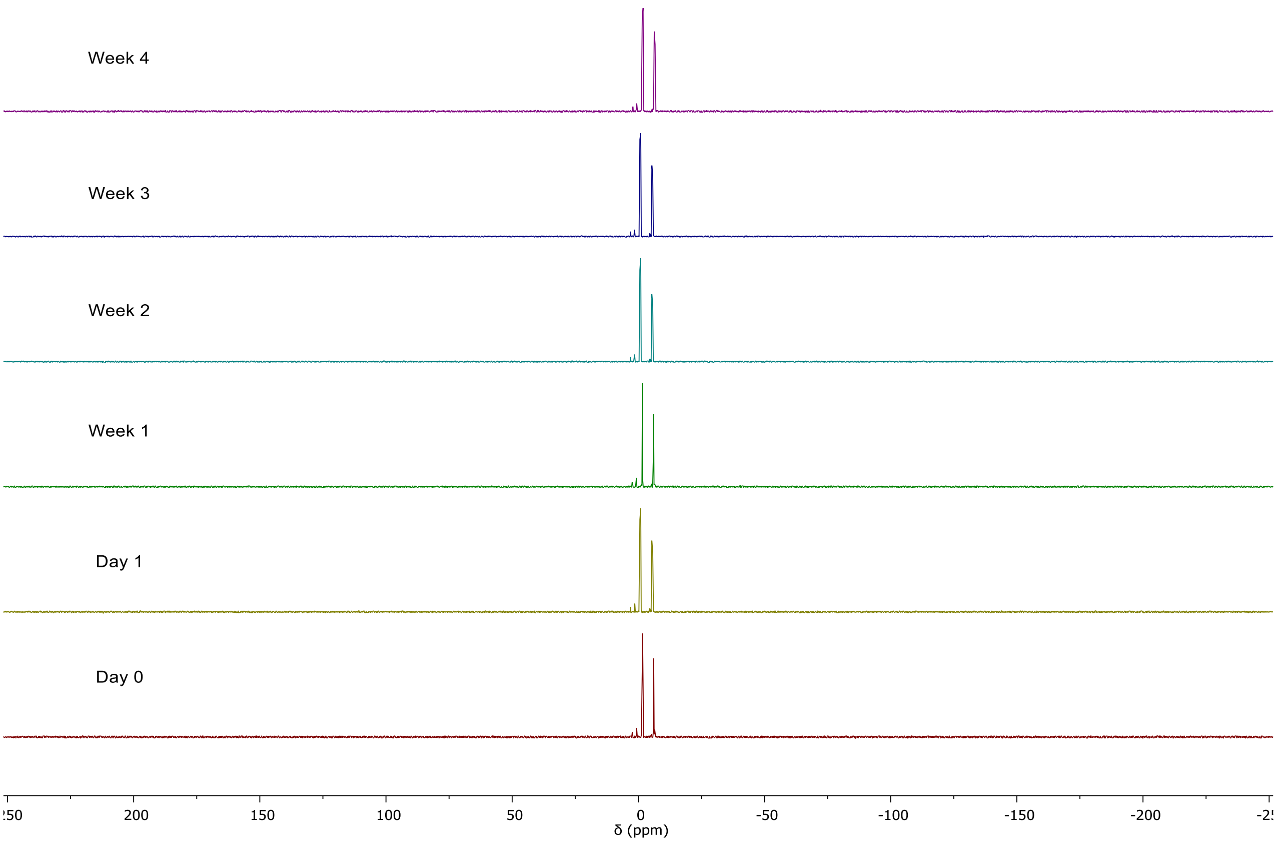

Figure S16: Overlaid $^{31}P\{^{1}H\}$ NMR spectrum of **2** in 1:9 H_2O/THF at different time points.

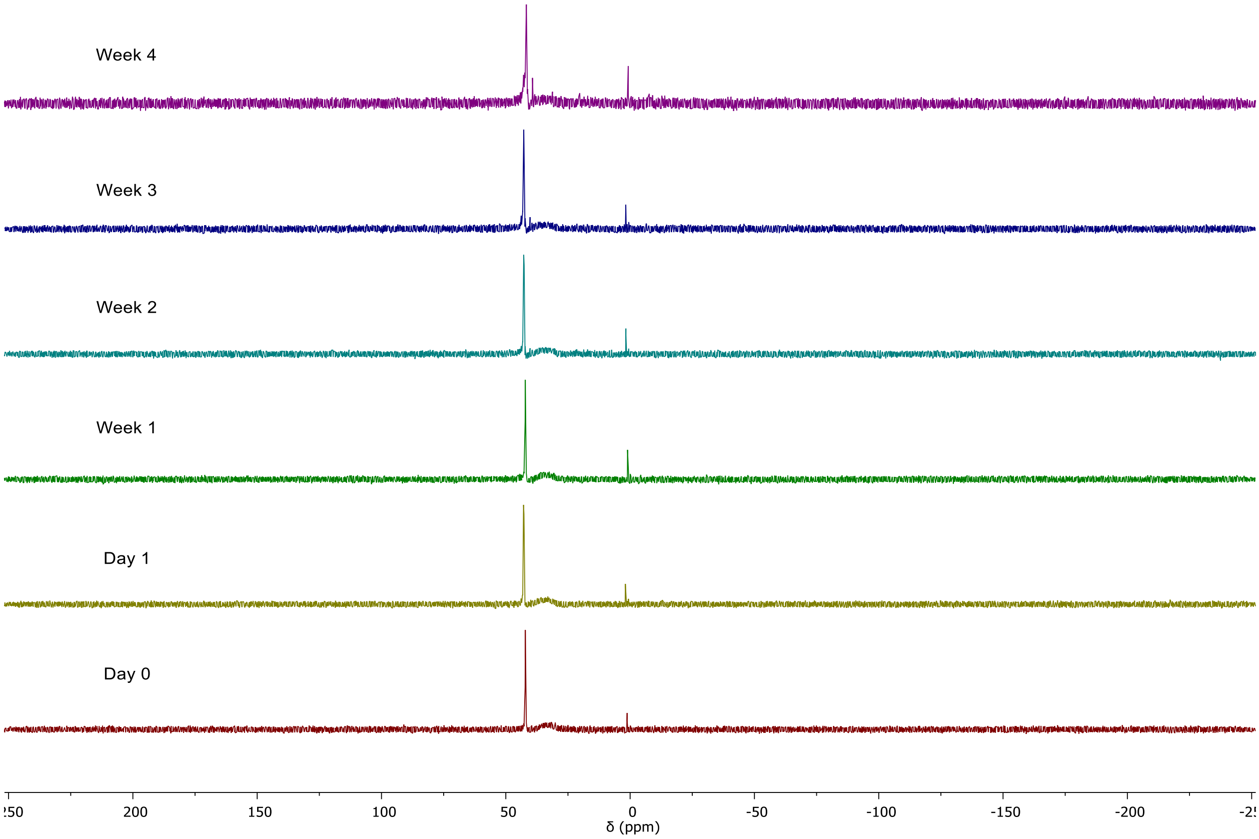

Figure S17: Overlaid $^{31}P\{^{1}H\}$ NMR spectrum of **3** in 1:9 H_2O/THF at different time points.

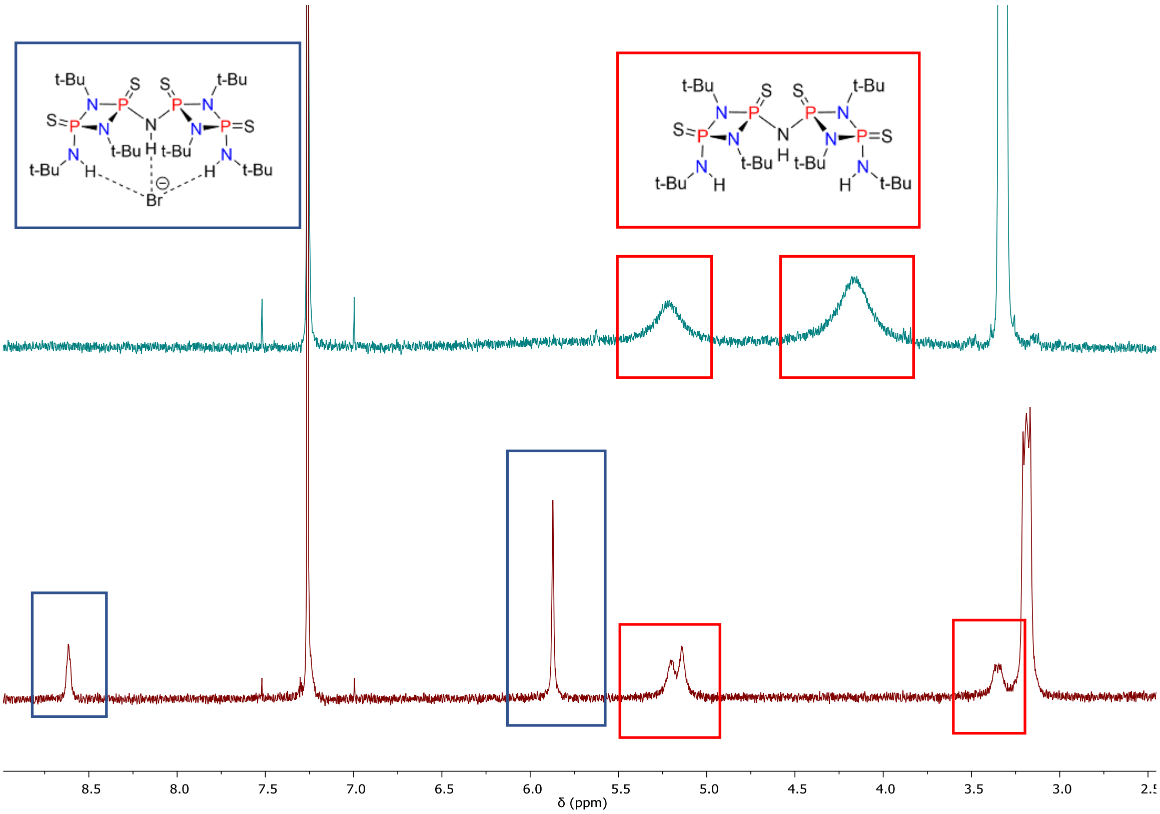

Figure S18: Overlaid ^{1}H NMR of 10 mM of **3** with 0.5 equivalents of TBABr in $CDCl_3$ at room temperature (top) and 223 K (bottom).

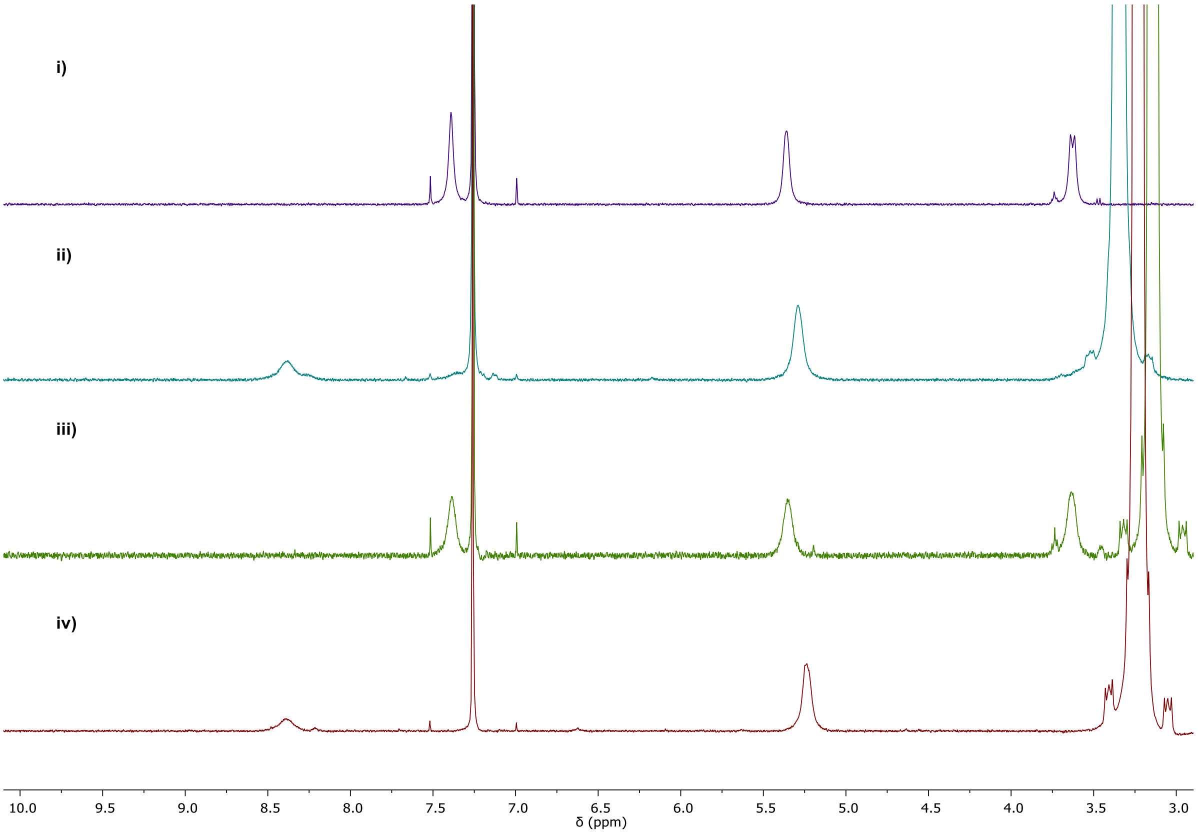

Figure S19: Overlaid ^{1}H NMR spectrum of (i) **2** in $CDCl_3$, (ii) **2** in $CDCl_3$ in the presence of 5 equivalents of TBACl, (iii) **2** in $CDCl_3$ in the presence of 5 equivalents of TBACl followed by addition of 5 equivalents of $NaPF_6$ (stirred overnight) and (iv) **2** in $CDCl_3$ in the presence of 5 equivalents of TBACl followed by addition of 5 equivalents of $NaPF_6$ (stirred overnight) followed by an additional 5 equivalents of TBACl.

To understand compound **3**'s preference to certain anions, competitive anion studies using ESI mass spectroscopy were conducted in negative mode with **3** in the presence of 10 equivalents of two different anions. From the spectrums below (**Figure S20-28**), we can deduce that **3** has a strong preference for smaller halides (such as Cl and Br), with the binding preference being in the order: Cl > Br > I ≈ NO_3 ≈ HSO_4. In all cases, deprotonation was observed which is attributed to the harsh condition of mass spectroscopy as evident from the sample containing only **3** (**Figure S29**).

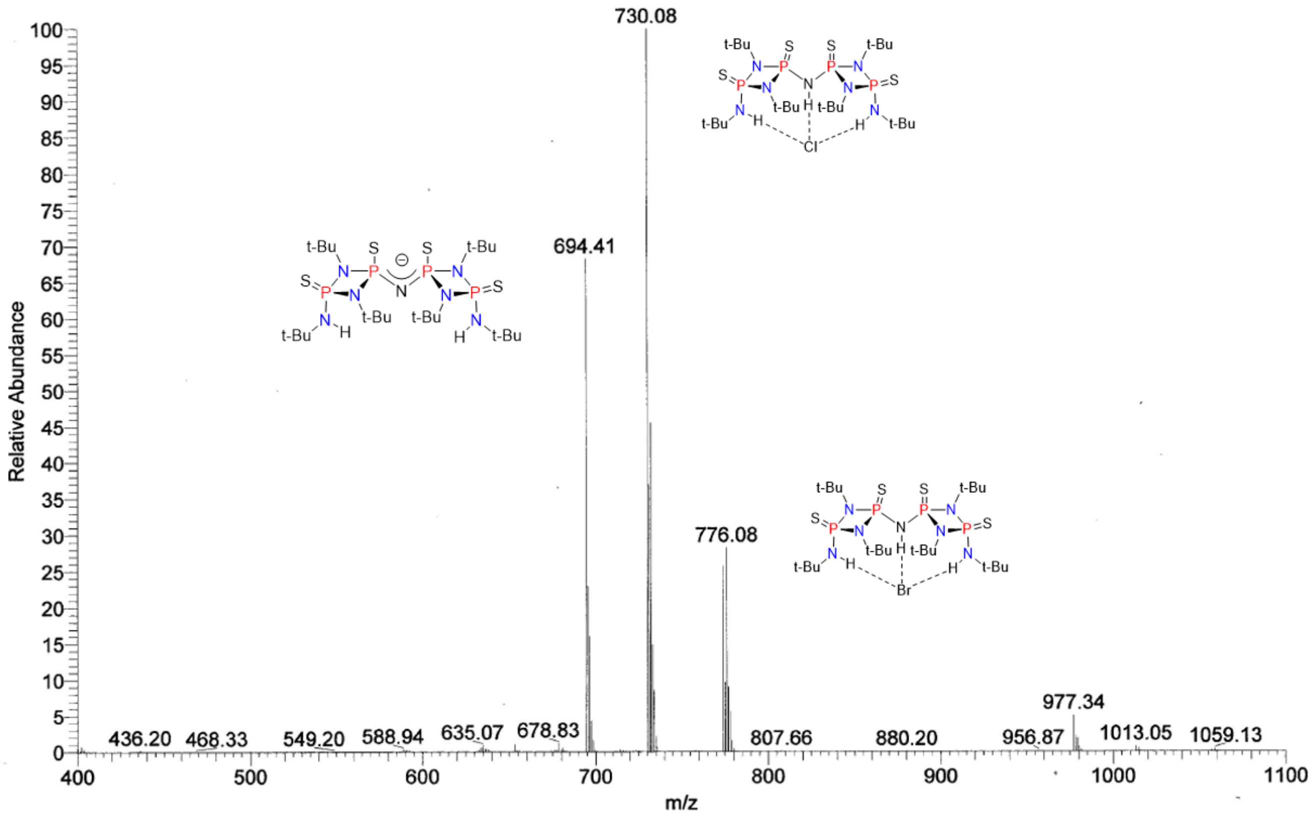

Figure S20: ESI-MS of **3** in the presence of 10 equivalents of TBACl and 10 equivalents of TBABr.

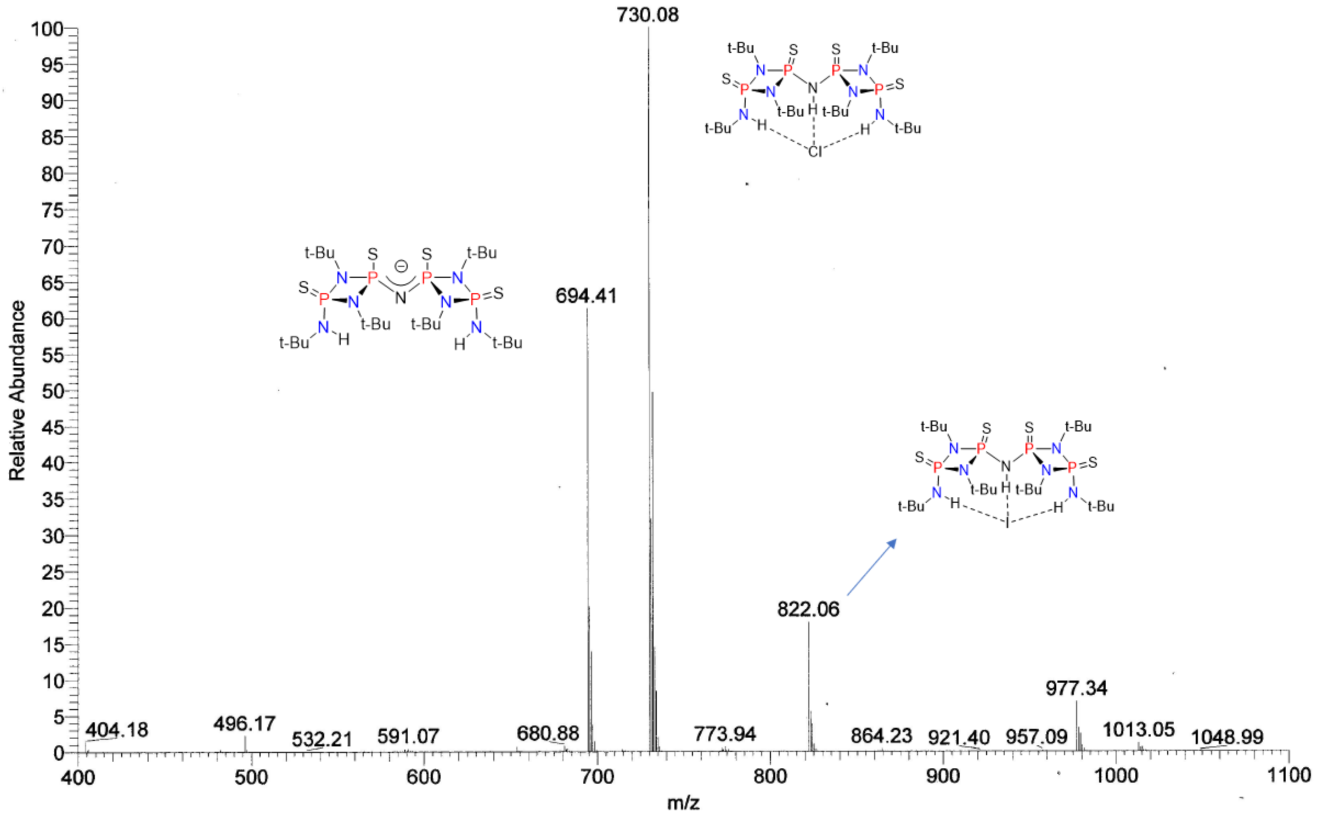

Figure S21: ESI-MS of **3** in the presence of 10 equivalents of TBACl and 10 equivalents of TBAI.

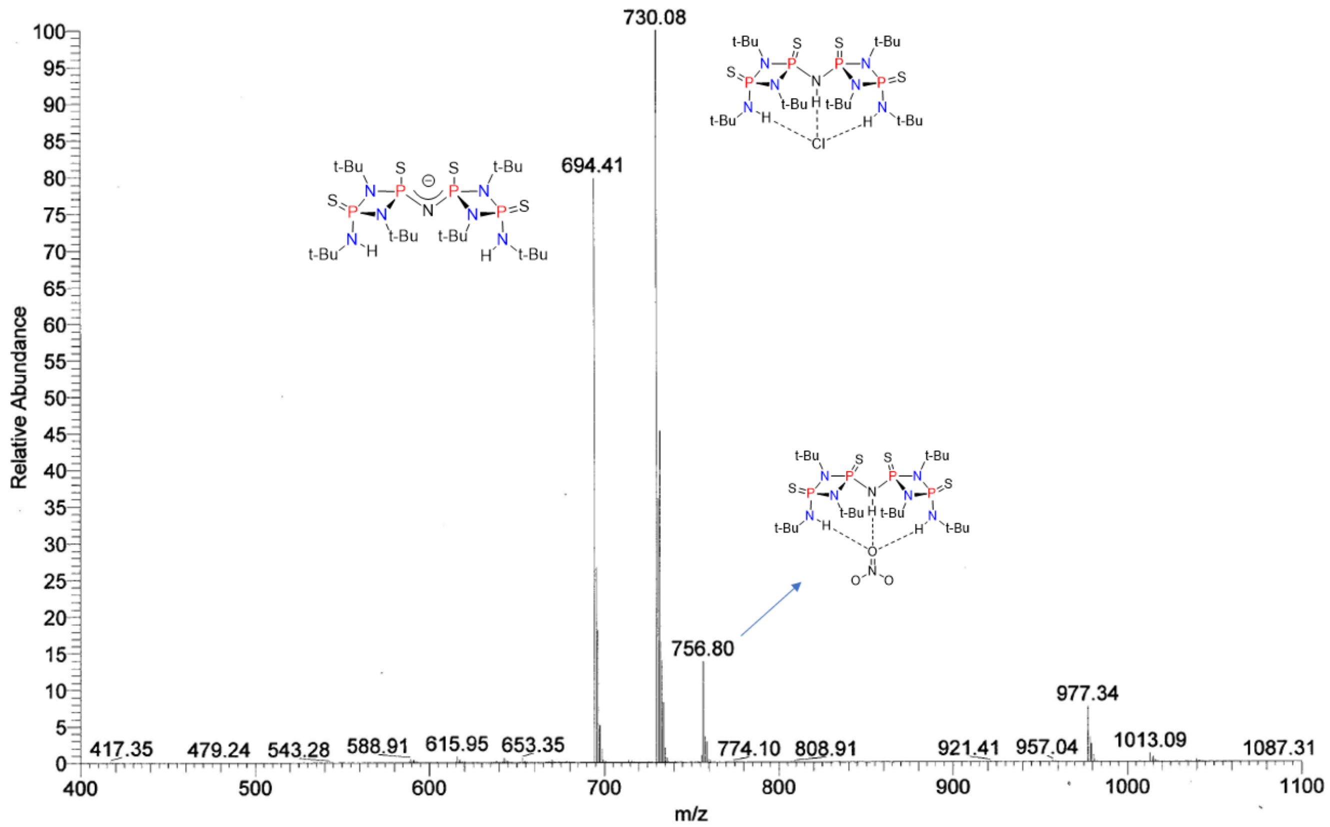

Figure S22: ESI-MS of **3** in the presence of 10 equivalents of TBACl and 10 equivalents of $TBANO_3$.

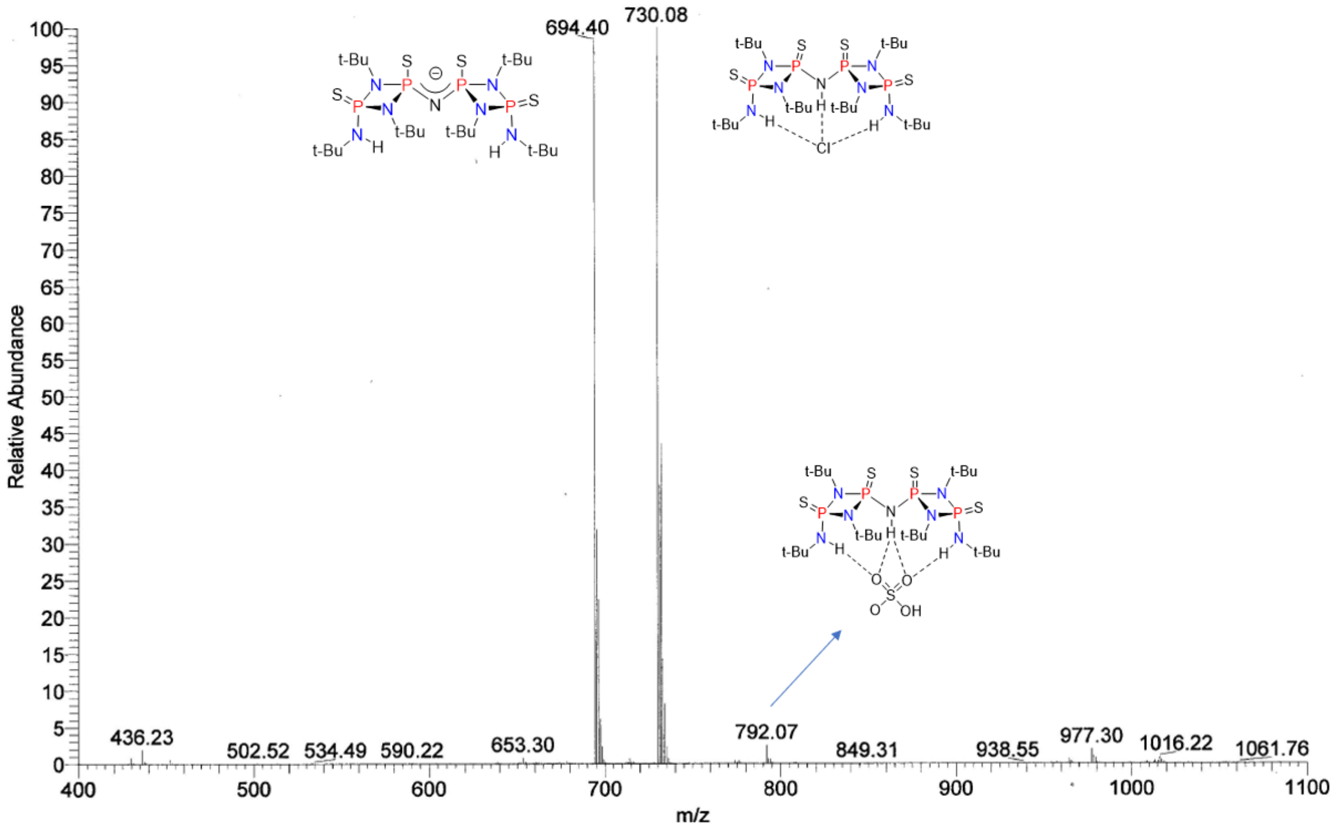

Figure S23: ESI-MS of **3** in the presence of 10 equivalents of TBACl and 10 equivalents of $TBAHSO_4$.

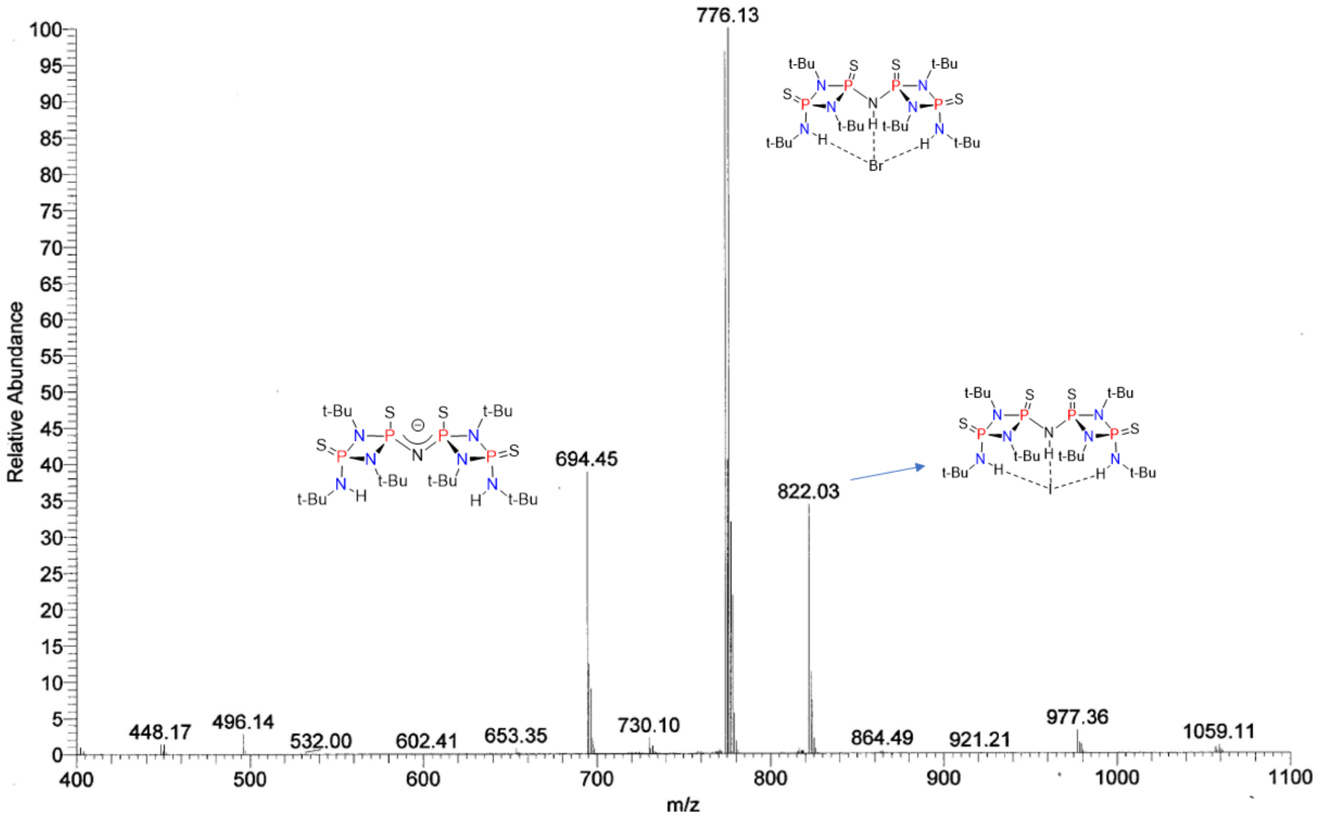

Figure S24: ESI-MS of **3** in the presence of 10 equivalents of TBABr and 10 equivalents of TBAI.

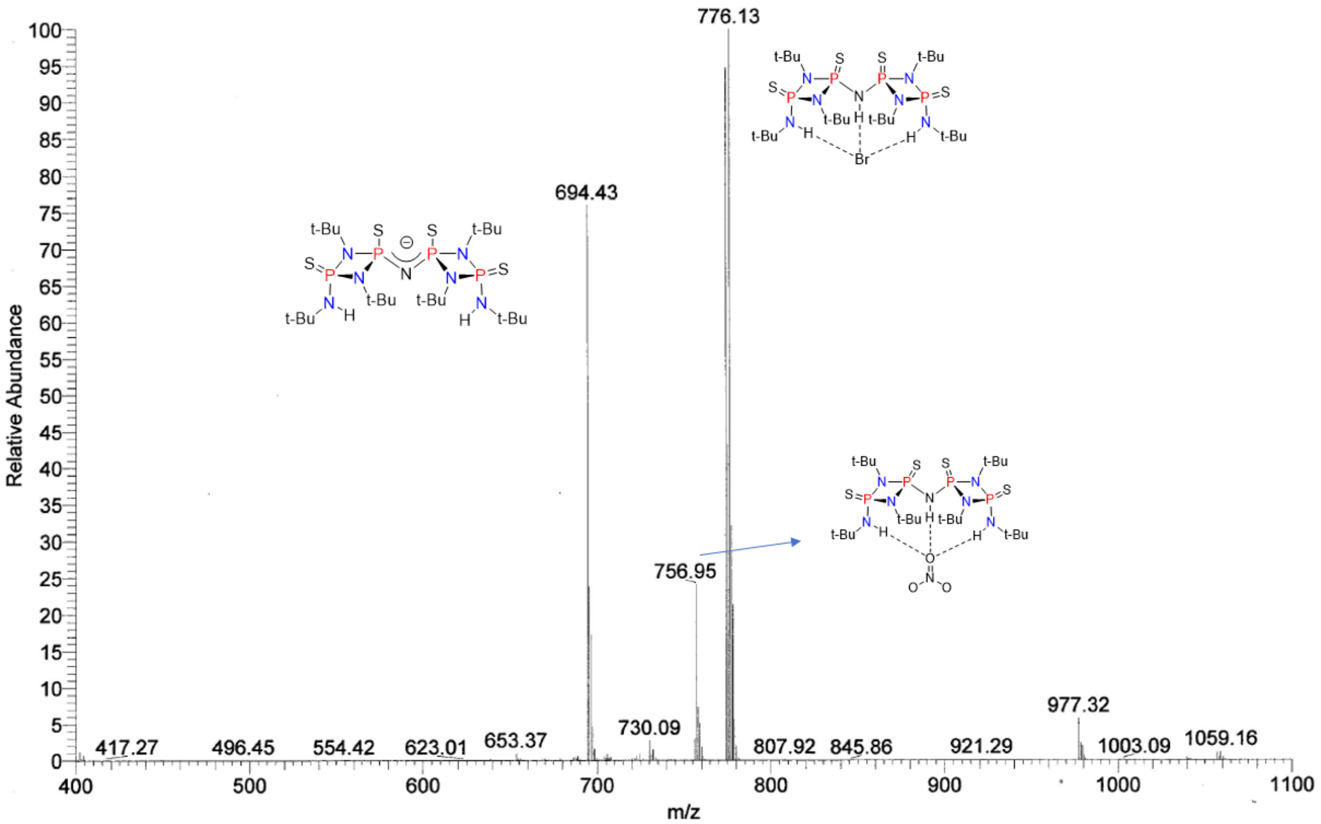

Figure S25: ESI-MS of **3** in the presence of 10 equivalents of TBABr and 10 equivalents of $TBANO_3$.

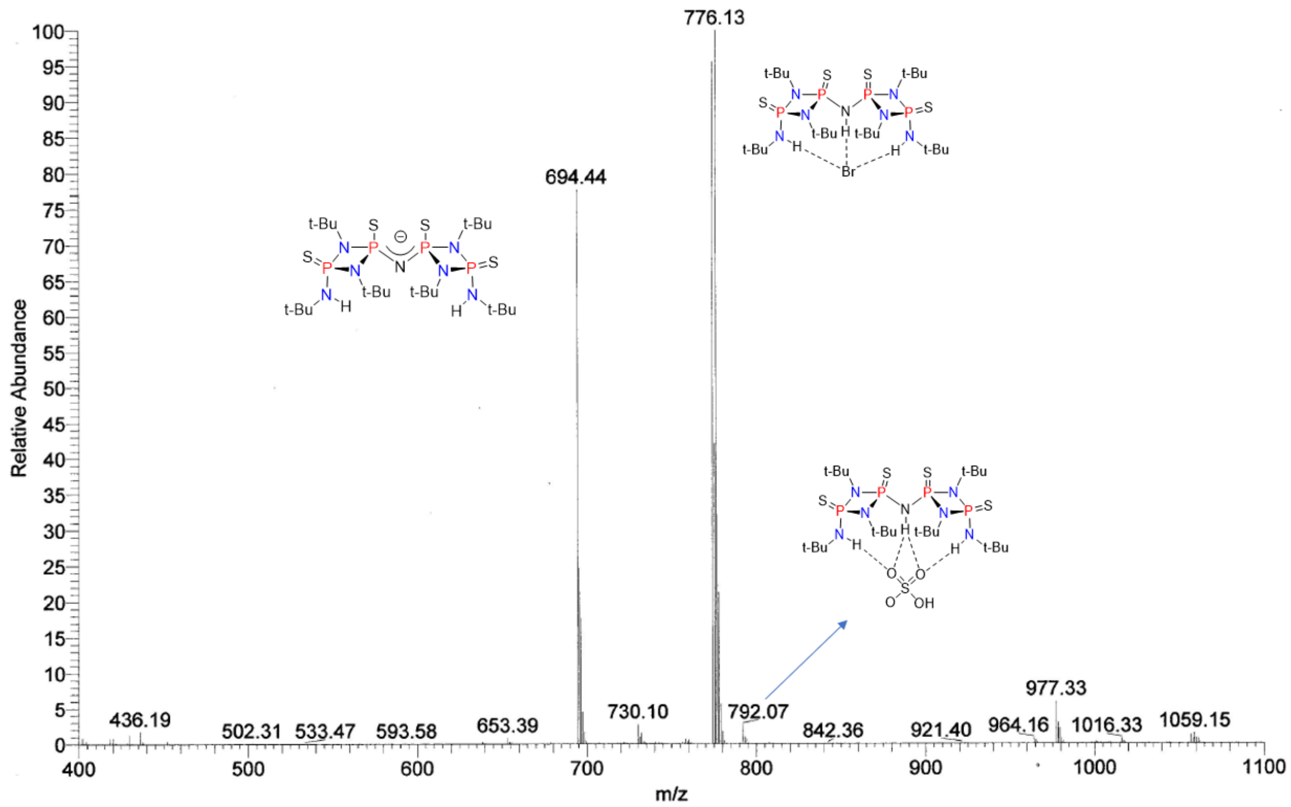

Figure S26: ESI-MS of **3** in the presence of 10 equivalents of TBABr and 10 equivalents of $TBAHSO_4$.

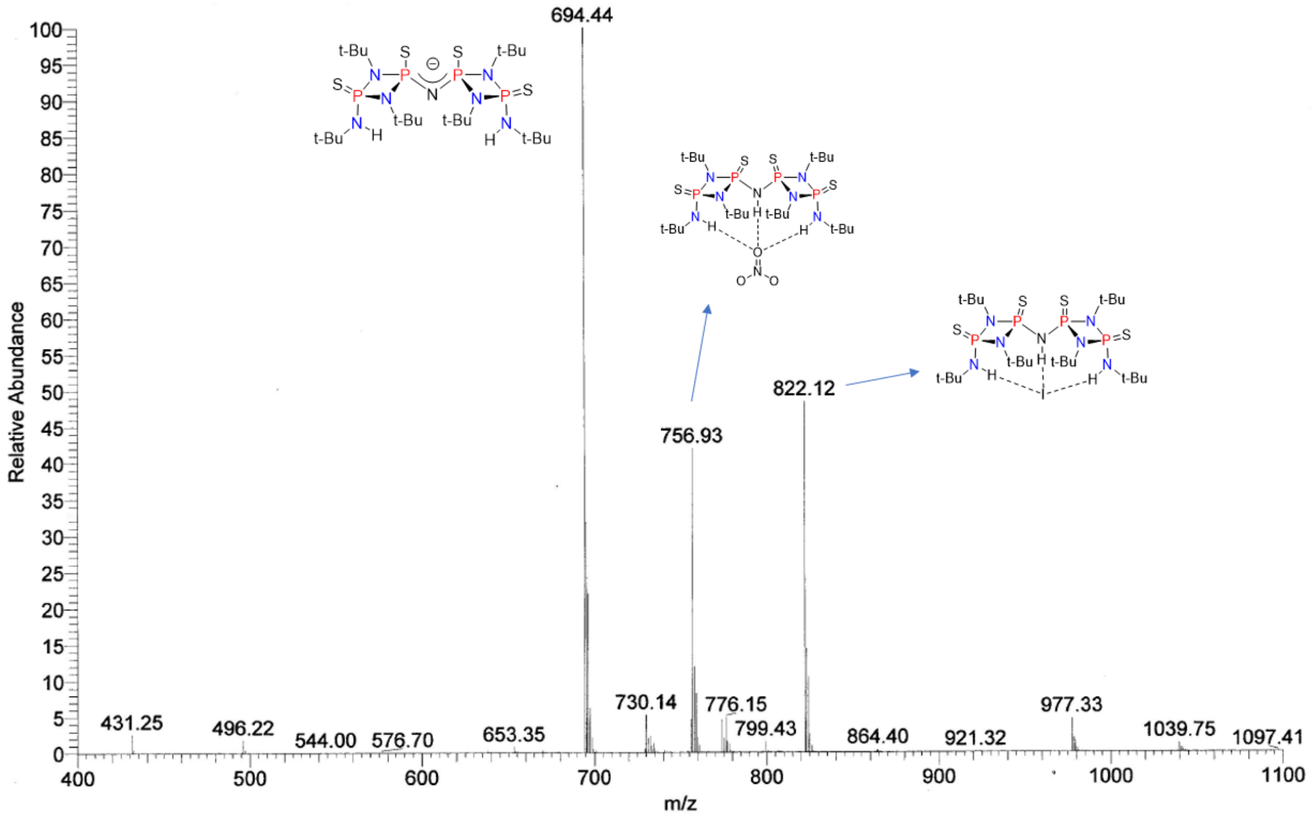

Figure S27: ESI-MS of **3** in the presence of 10 equivalents of TBAI and 10 equivalents of $TBANO_3$.

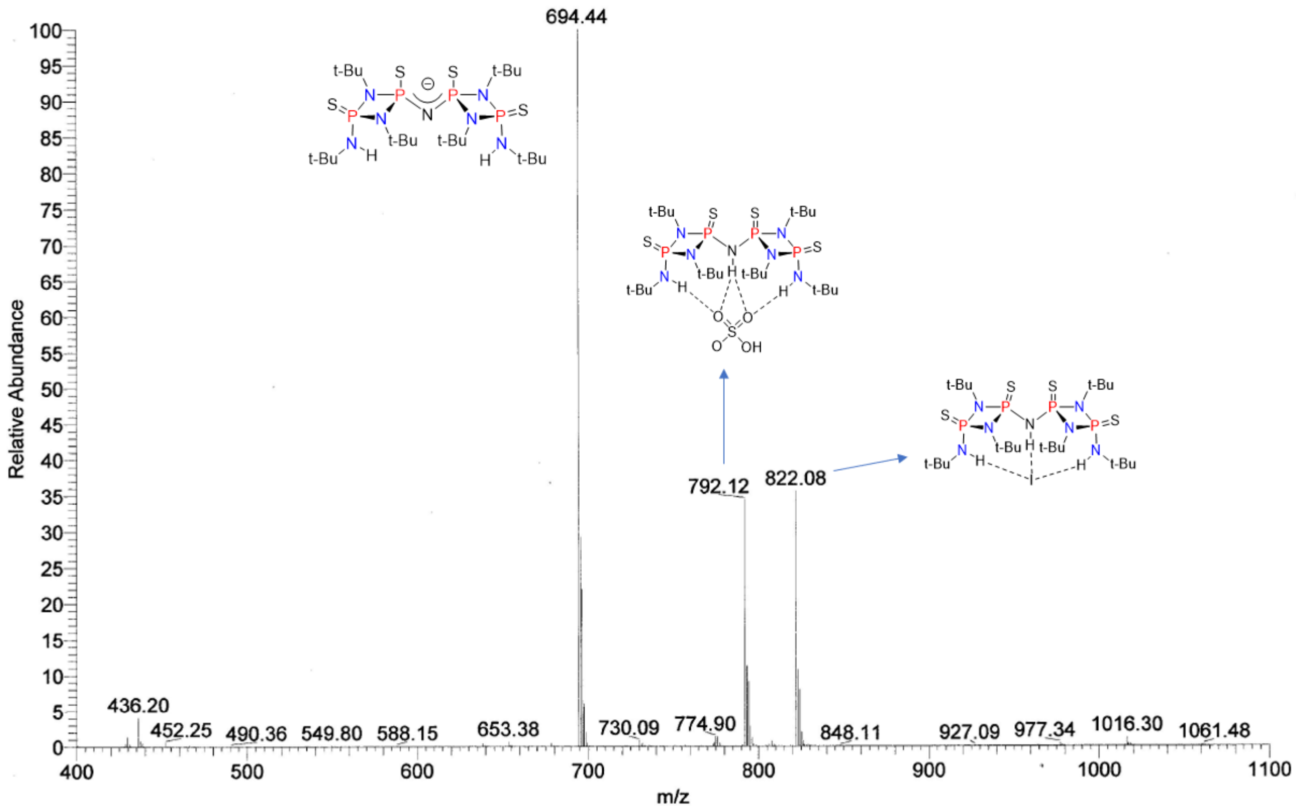

Figure S28: ESI-MS of **3** in the presence of 10 equivalents of TBAI and 10 equivalents of TBAHSO4.

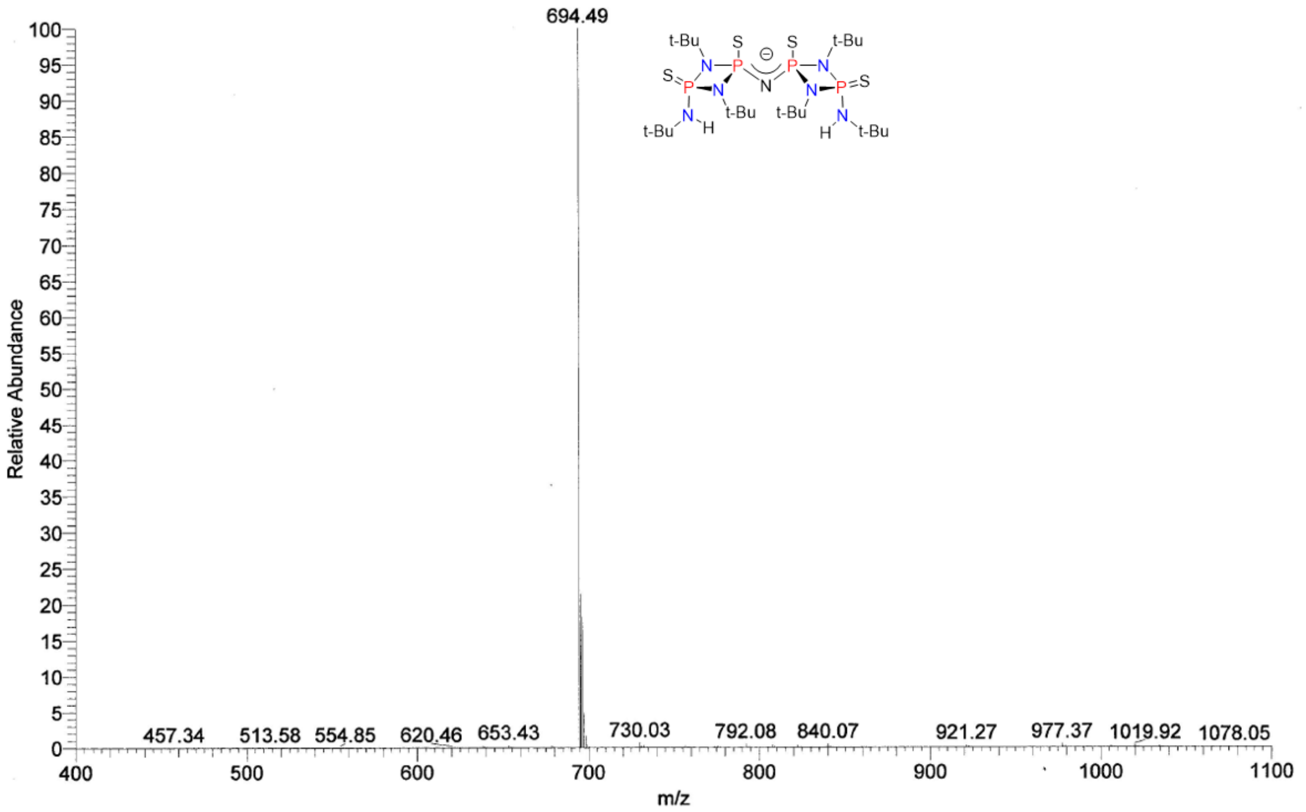

Figure S29: ESI-MS of **3** without the presence of additional anions showcasing that deprotonation is a result of the harsh conditions within mass spectroscopy.

4. Binding Studies

Anion binding abilities of **3**[ON] was benchmarked against monomeric $P^{V}_2N_2$ species. An analogous study was performed for monomeric [^{t}BuHNPE(μ-N^{t}Bu)]$_2$ (E = S and Se for **3**[m] and **4**[m], respectively) in $CDCl_3$ (**Table S1**). Compound **4**[m], previously reported as the best monomeric counterpart, exhibits increased affinity for Cl^- over **3**[m] which is in line with previous reports.[5] Notably, among **3**[m] and **4**[m], binding strengths decrease as the size of anions increases, with decreasing binding strength differences for larger anions, such as Br^-, I^-, HSO_4^- and NO_3^-, along with low affinities for HSO_4^-. This is attributed to both the reduced number of NH HB donors as well as the smaller cavity size within the monomeric $P^{V}_2N_2$, which results in lower binding affinities across all anions studied - except for Br^-, *vide supra*.

Table S1: Comparison of binding constants of **3** *vs* **3**[m] and **4**[m].

**Anion**	**Binding constants, K_A [M^{-1}]**		
	**3**	**3**[m]	**4**[m]
	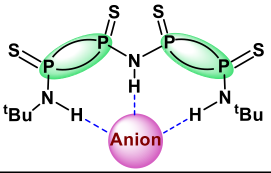	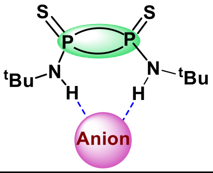	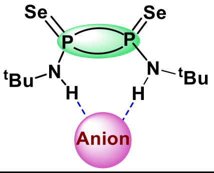
**Cl^-**	192.92 ± 81.89[a]	3.66 ± 0.13	4.41 ± 0.20
**Br^-**	**N.D.**	2.60 ± 0.07	2.96 ± 0.12
**I^-**	5.17 ± 0.13	2.11 ± 0.06	2.00 ± 0.04
**HSO_4^-**	9.48 ± 0.25	0.49 ± 0.02	0.48 ± 0.01
**NO_3^-**	20.39 ± 1.17	6.24 ± 0.19	5.96 ± 0.21

[a] Estimated based on concentration-weighted average of [H_0] and [HG][6]; N.D. = not determined.

4.1 Dimeric S (3)

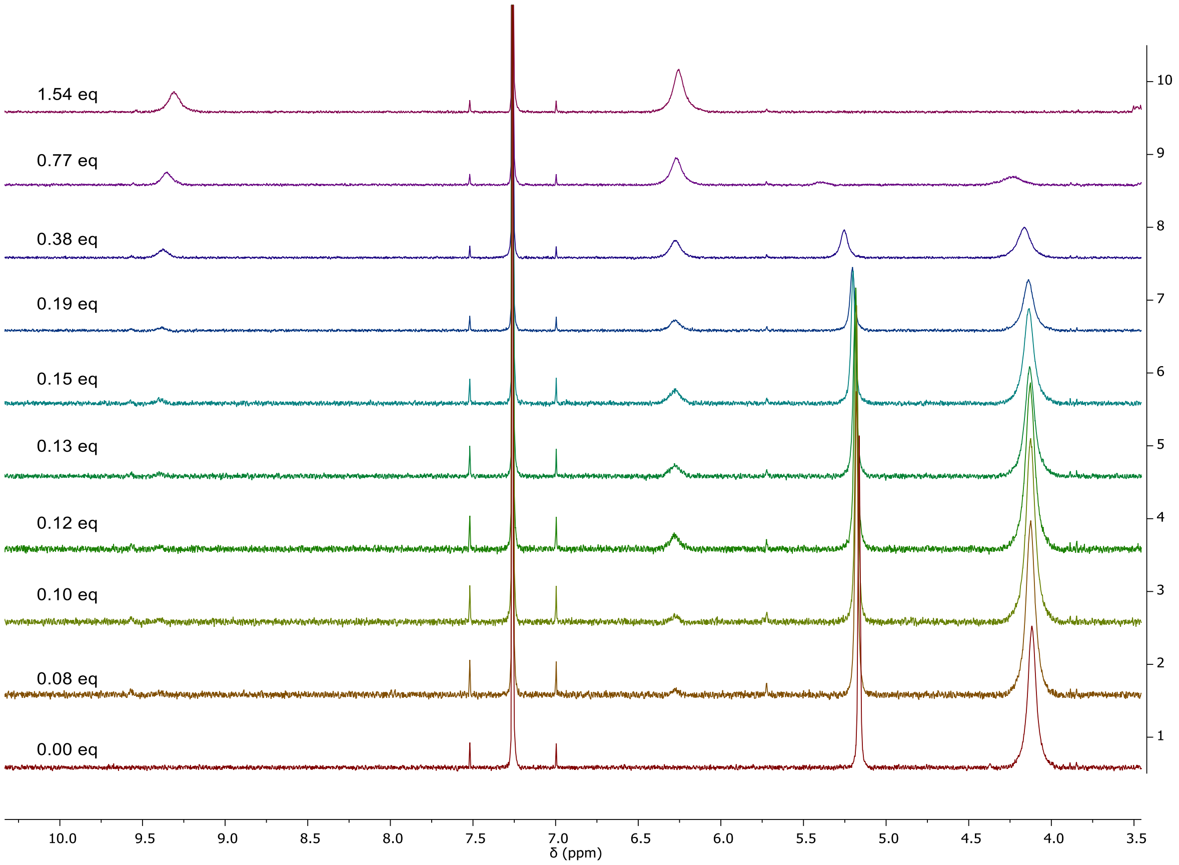

Figure S30: ^{1}H NMR titration (25 °C, $CDCl_3$, 400 MHz) of **3** with increasing amounts of TBACl.

Due to the neligible exchange of chloride ions between host molecules at room temperature, the chemical shifts of the NH protons due to fast equilibirum was estimated using a concentration-weighted average of free host and host-guest complex,

$$\Delta\delta = \delta_{\Delta HG}\left(\frac{[HG]}{[H]_0}\right)$$

Table S2: Estimated values of NH protons chemical shifts based on concentration-weighted average of free host and host-guest complex of **3** and chloride.

	Terminal NH			Internal NH		
Guest:Host (eq)	$\delta_{\Delta HG}$ (ppm)	$[HG]/[H_0]$ (ppm)	Δδ (ppm)	$\delta_{\Delta HG}$ (ppm)	$[HG]/[H_0]$ (ppm)	Δδ (ppm)
0.00	0.00	0.00	0.00	0.00	0.00	0.00
0.08	2.15	0.15	0.32	4.21	0.16	0.68
0.10	2.15	0.17	0.37	4.20	0.21	0.89
0.12	2.15	0.25	0.54	4.21	0.23	0.97
0.13	2.15	0.29	0.63	4.21	0.25	1.06
0.15	2.14	0.31	0.67	4.19	0.25	1.06
0.19	2.14	0.34	0.73	4.18	0.32	1.35
0.38	2.11	0.42	0.88	4.12	0.38	1.56
0.77	2.04	0.60	1.23	3.95	0.59	2.35
1.54	2.14	1.00	2.14	4.14	1.00	4.14

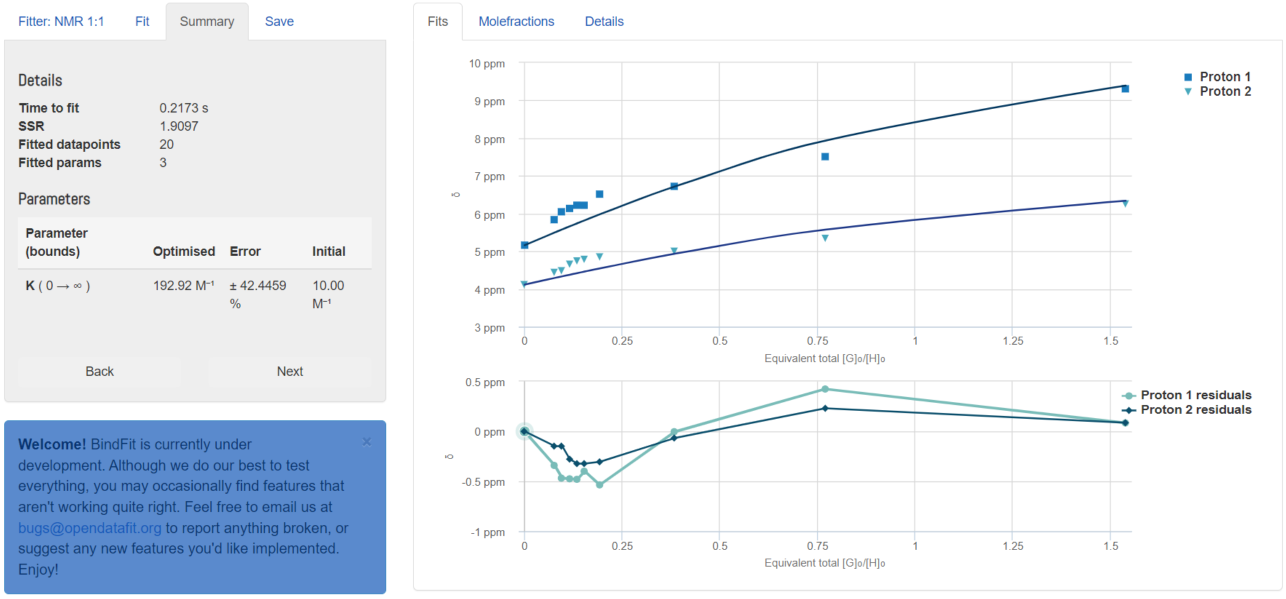

Figure S31: Binding isotherm (1:1 system) fitted to the ^{1}H NMR chemical shift of the NH protons in **3** with increasing amounts of TBACl after estimation using the concentration-weighted average of free host and host-guest complex.[6] Due to this approximation based on integrals of the amino peaks, the error is approixmately 40% despite multiple titration runs. Despite that, these values serves as an illustration that binding affinity of **3** to chloride is much higher than those compared to other anions studied, even after accounting for the large error. Graph obtained using the bindfit tool from www.supramolecular.org.[6]

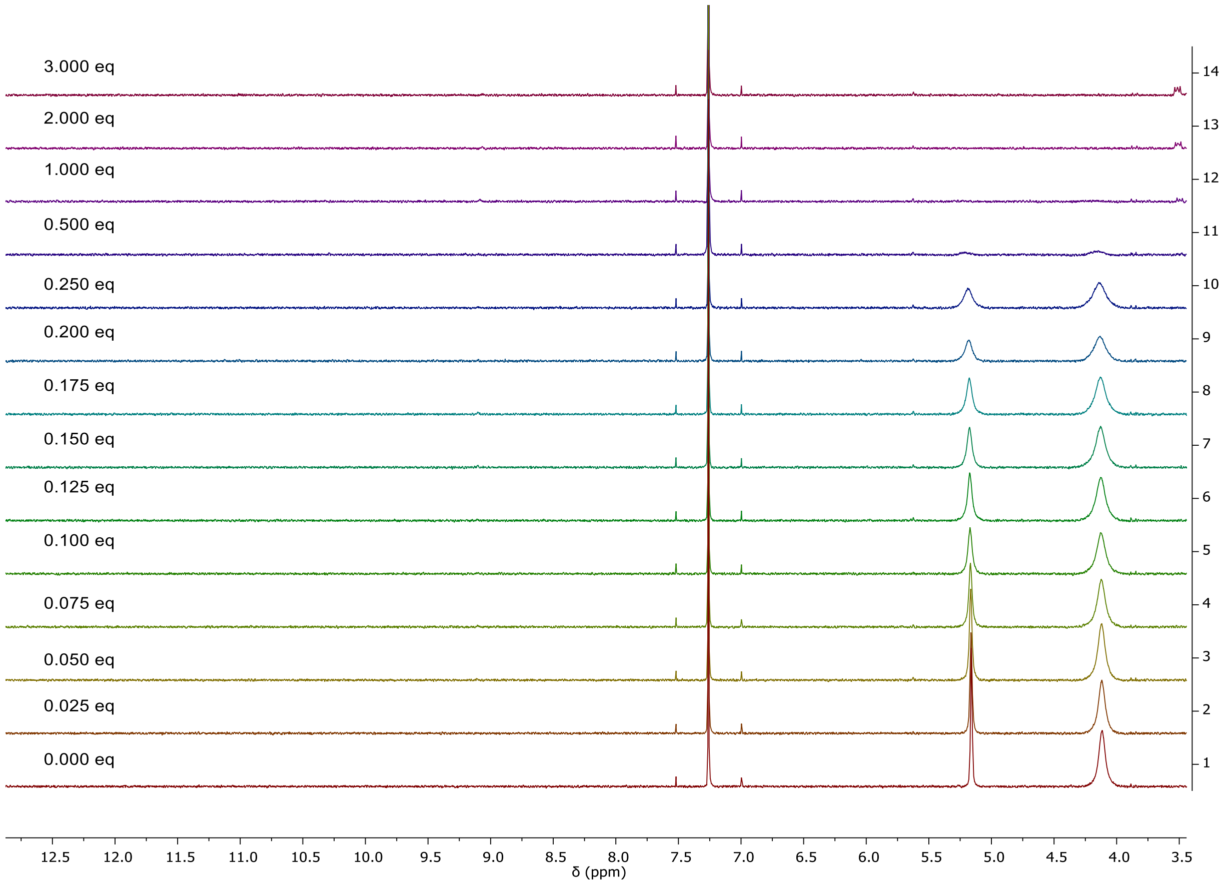

Figure S32: ^{1}H NMR titration (25 °C, $CDCl_3$, 400 MHz) of **3** with increasing amounts of TBABr.

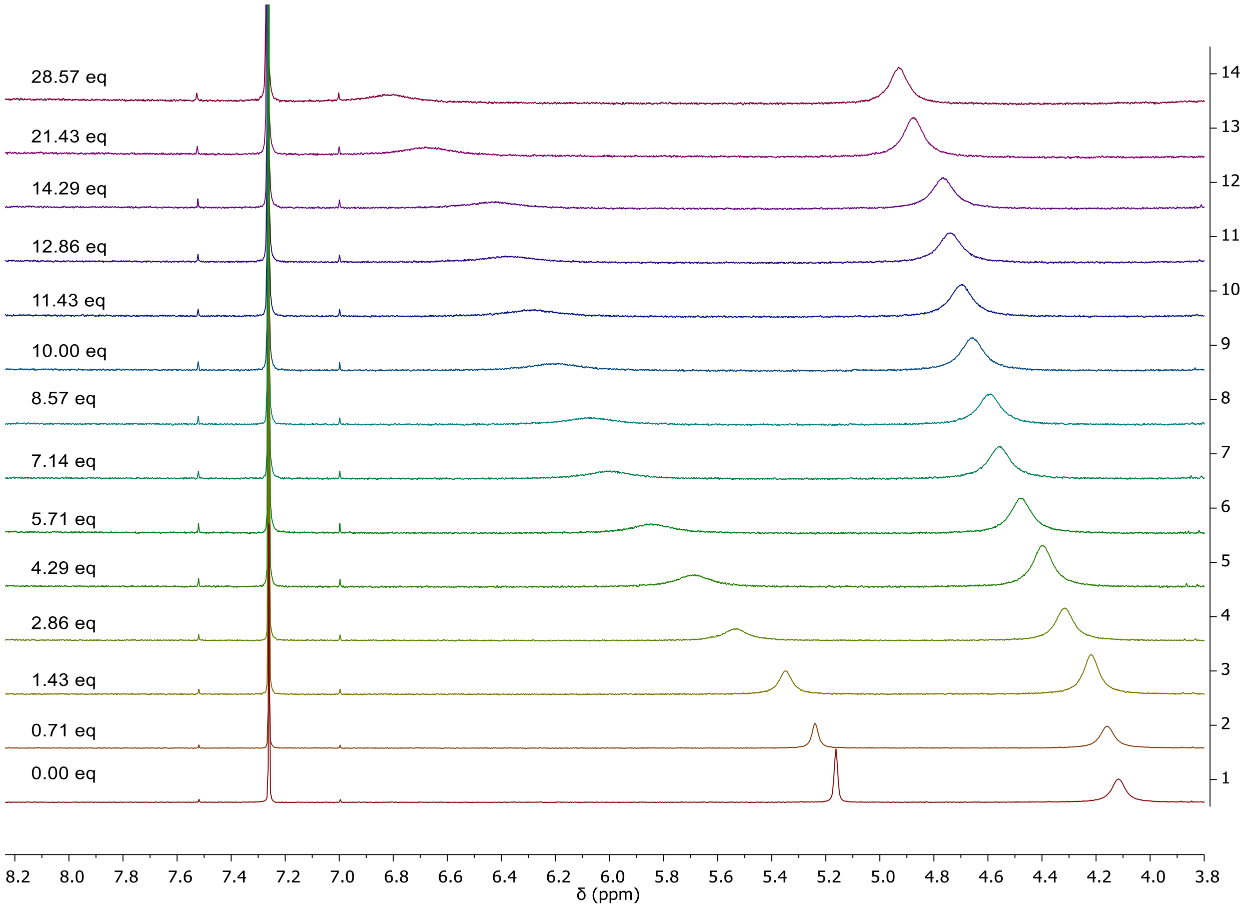

Figure S33: ^{1}H NMR titration (25 °C, $CDCl_3$, 400 MHz) of **3** with increasing amounts of TBAI.

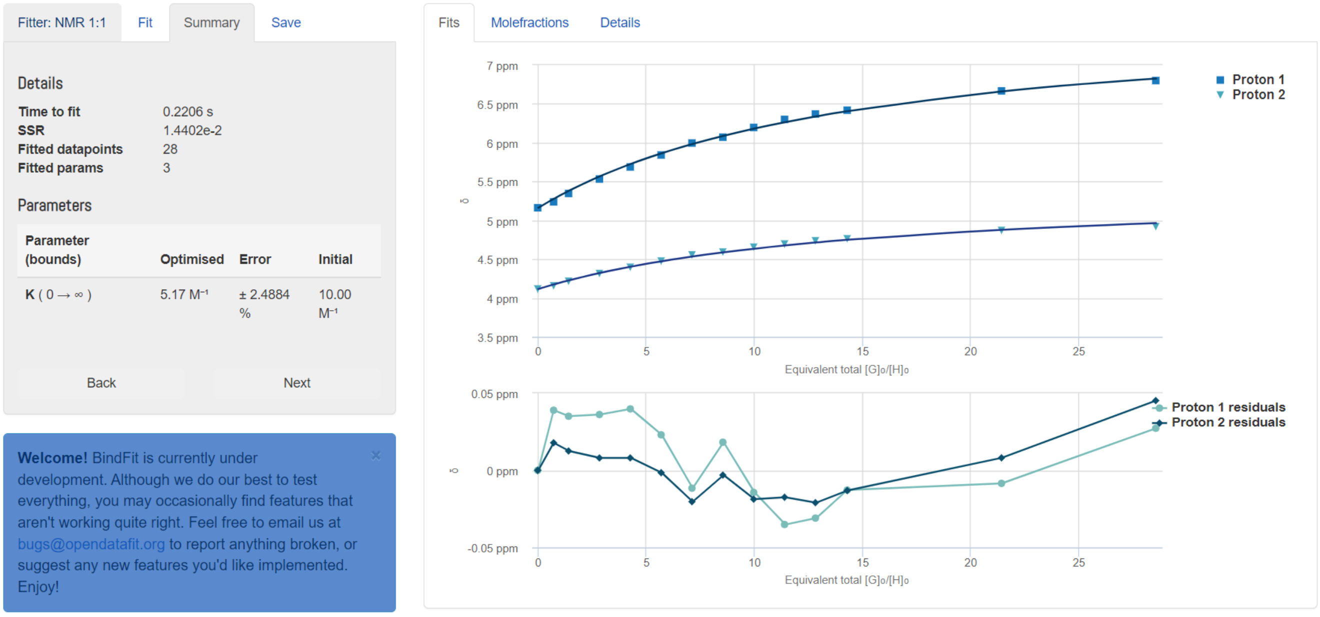

Figure S34: Binding isotherm (1:1 system) fitted to the ^{1}H NMR chemical shift of the NH protons in **3** with increasing amounts of TBAI. Graph obtained using the bindfit tool from www.supramolecular.org.[6]

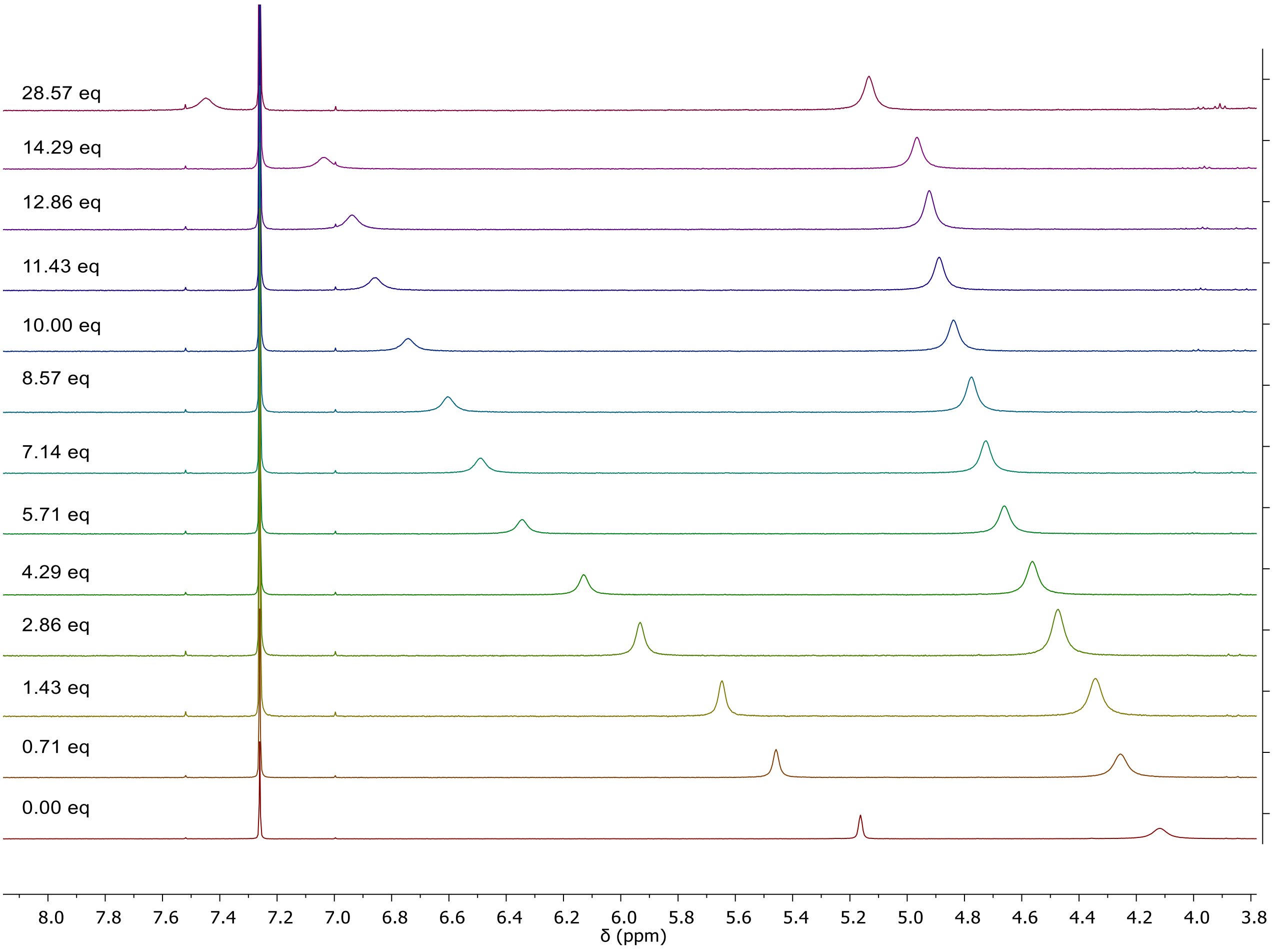

Figure S35: ^{1}H NMR titration (25 °C, $CDCl_3$, 400 MHz) of **3** with increasing amounts of $TBAHSO_4$.

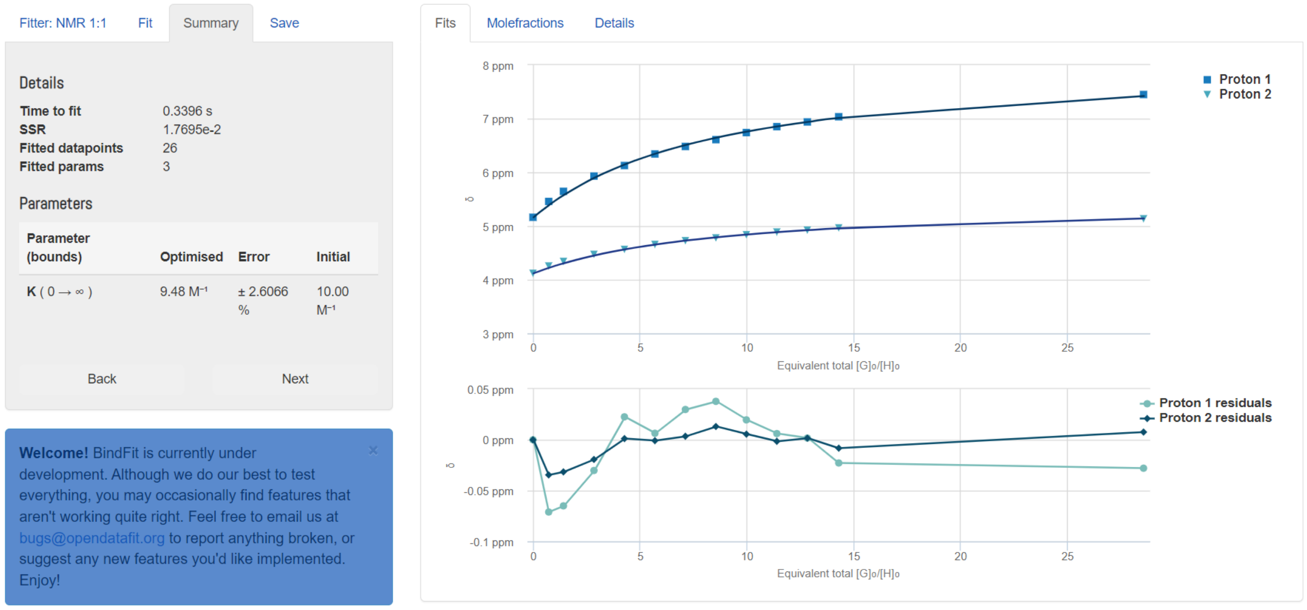

Figure S36: Binding isotherm (1:1 system) fitted to the ^1H NMR chemical shift of the NH protons in **3** with increasing amounts of TBAHSO$_4$. Graph obtained using the bindfit tool from www.supramolecular.org.[6]

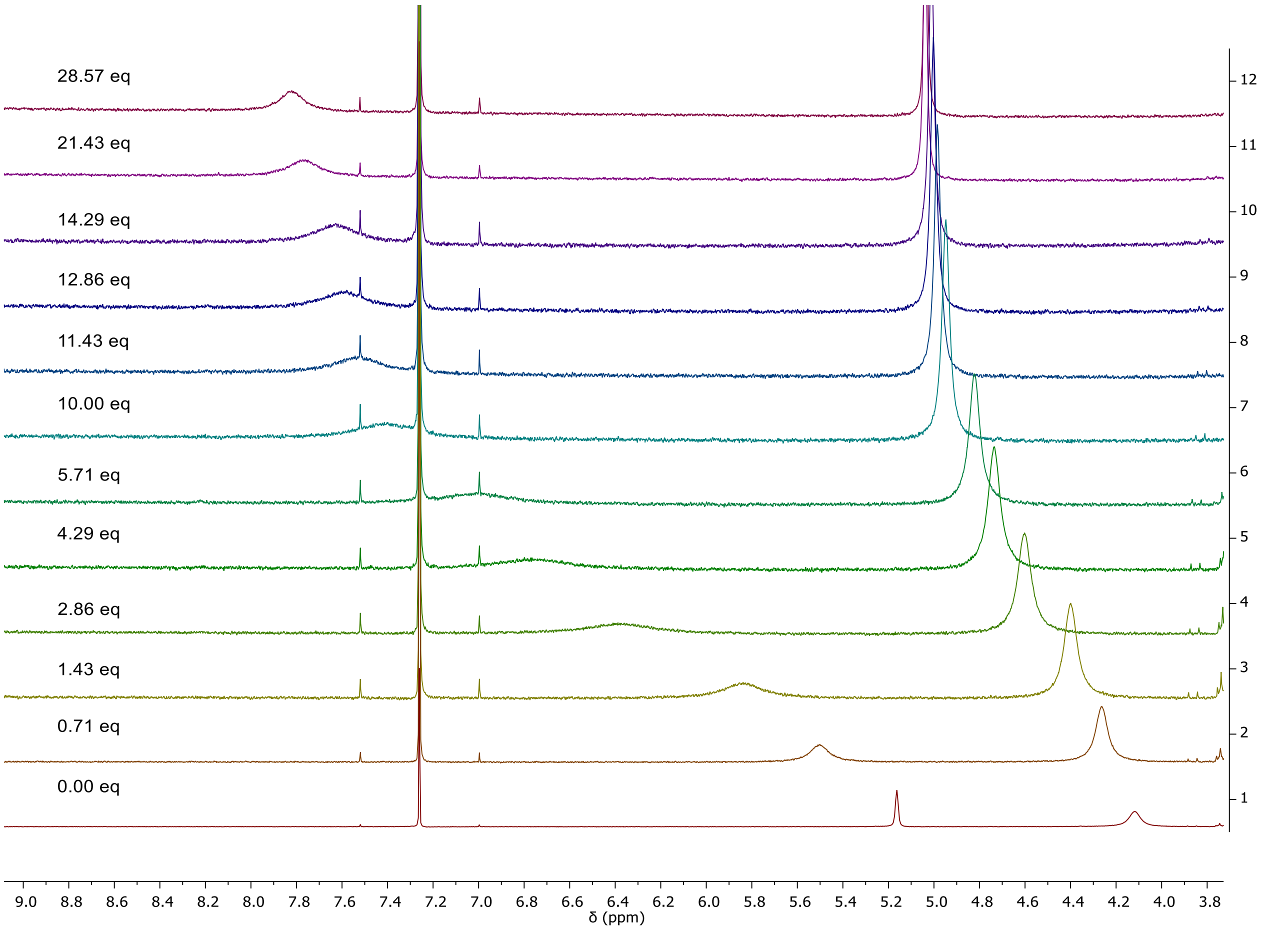

Figure S37: ^1H NMR titration (25 °C, CDCl$_3$, 400 MHz) of **3** with increasing amounts of TBANO$_3$.

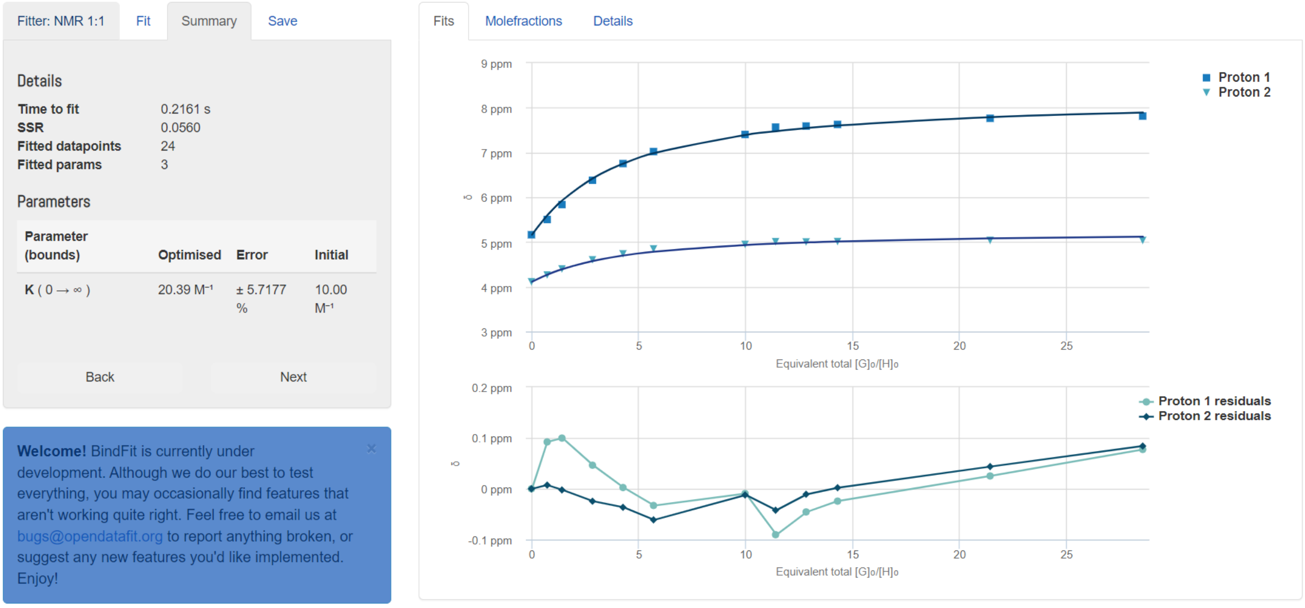

Figure S38: Binding isotherm (1:1 system) fitted to the ^{1}H NMR chemical shift of the NH protons in **3** with increasing amounts of $TBANO_3$. Graph obtained using the bindfit tool from www.supramolecular.org.[6]

4.2 Monomeric S (3^m)

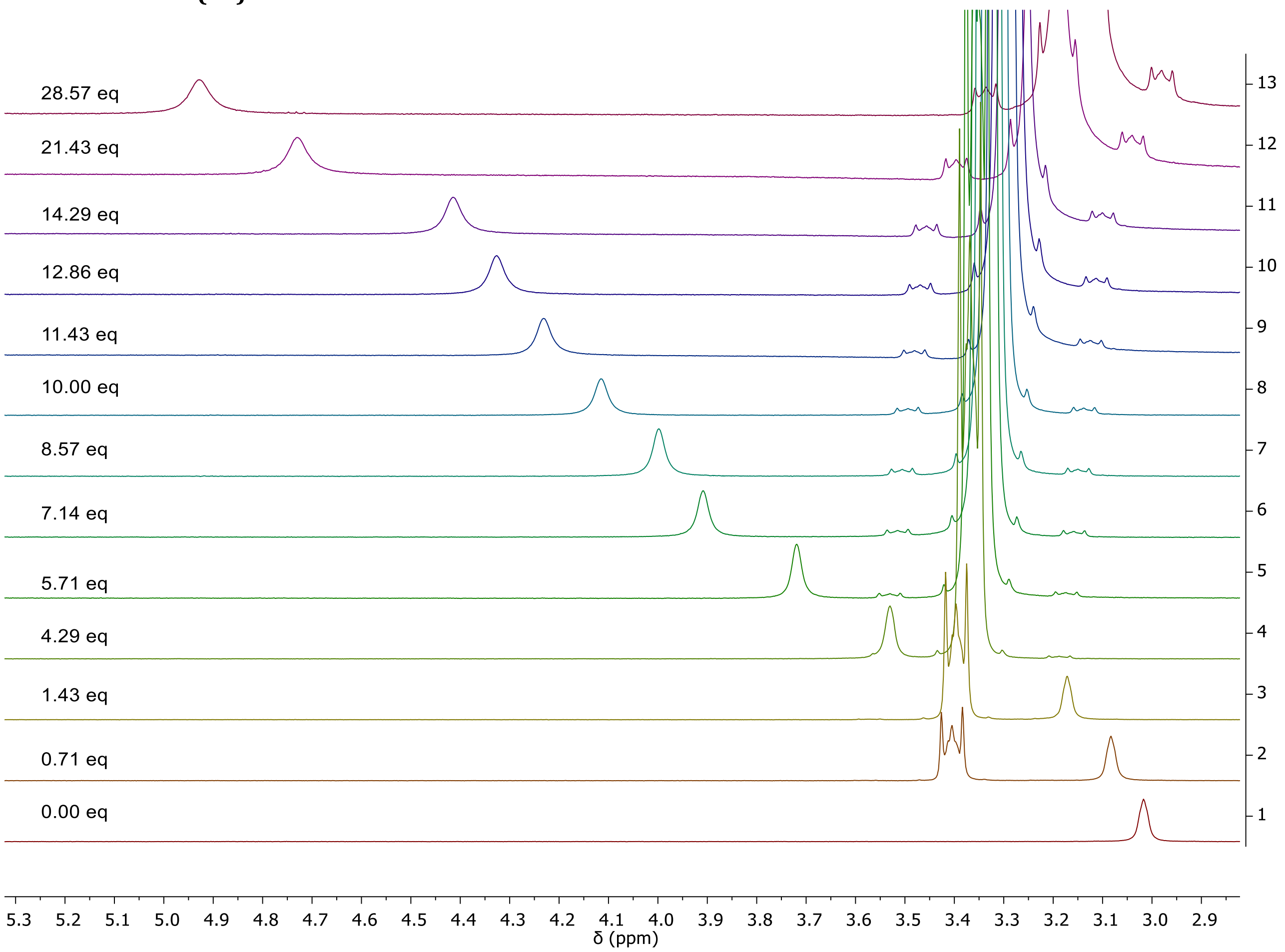

Figure S39: ^{1}H NMR titration (25 °C, $CDCl_3$, 400 MHz) of $\mathbf{3^m}$ with increasing amounts of TBACl.

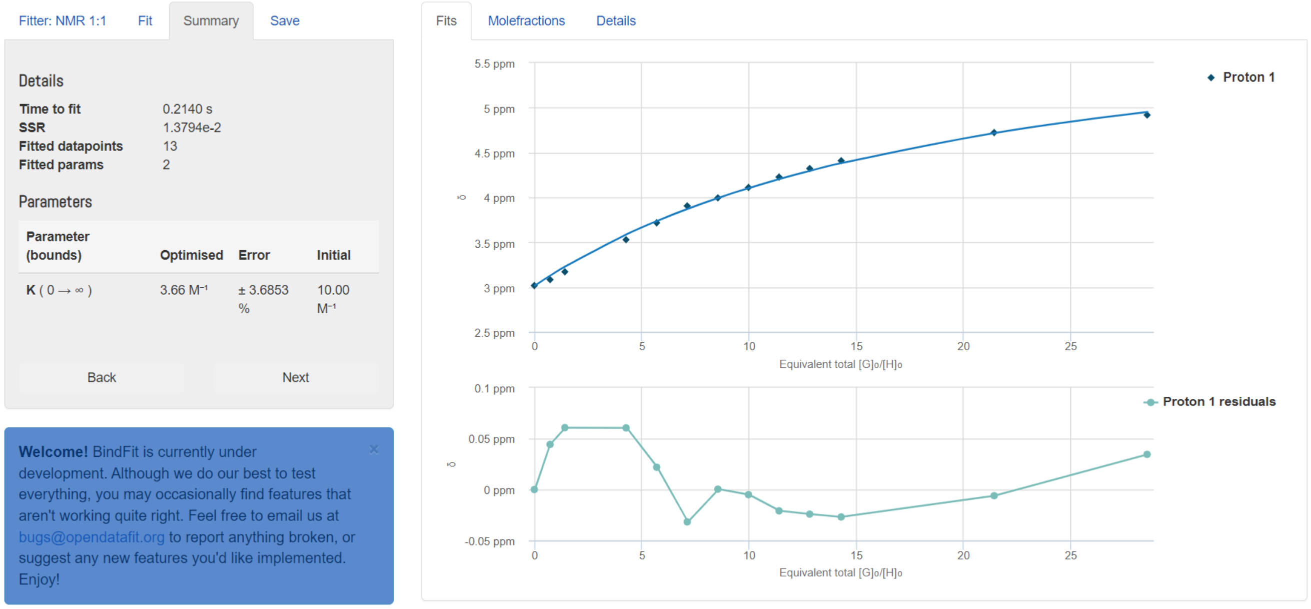

Figure S40: Binding isotherm (1:1 system) fitted to the ^{1}H NMR chemical shift of the NH protons in $\mathbf{3^m}$ with increasing amounts of TBACl. Graph obtained using the bindfit tool from www.supramolecular.org.[6]

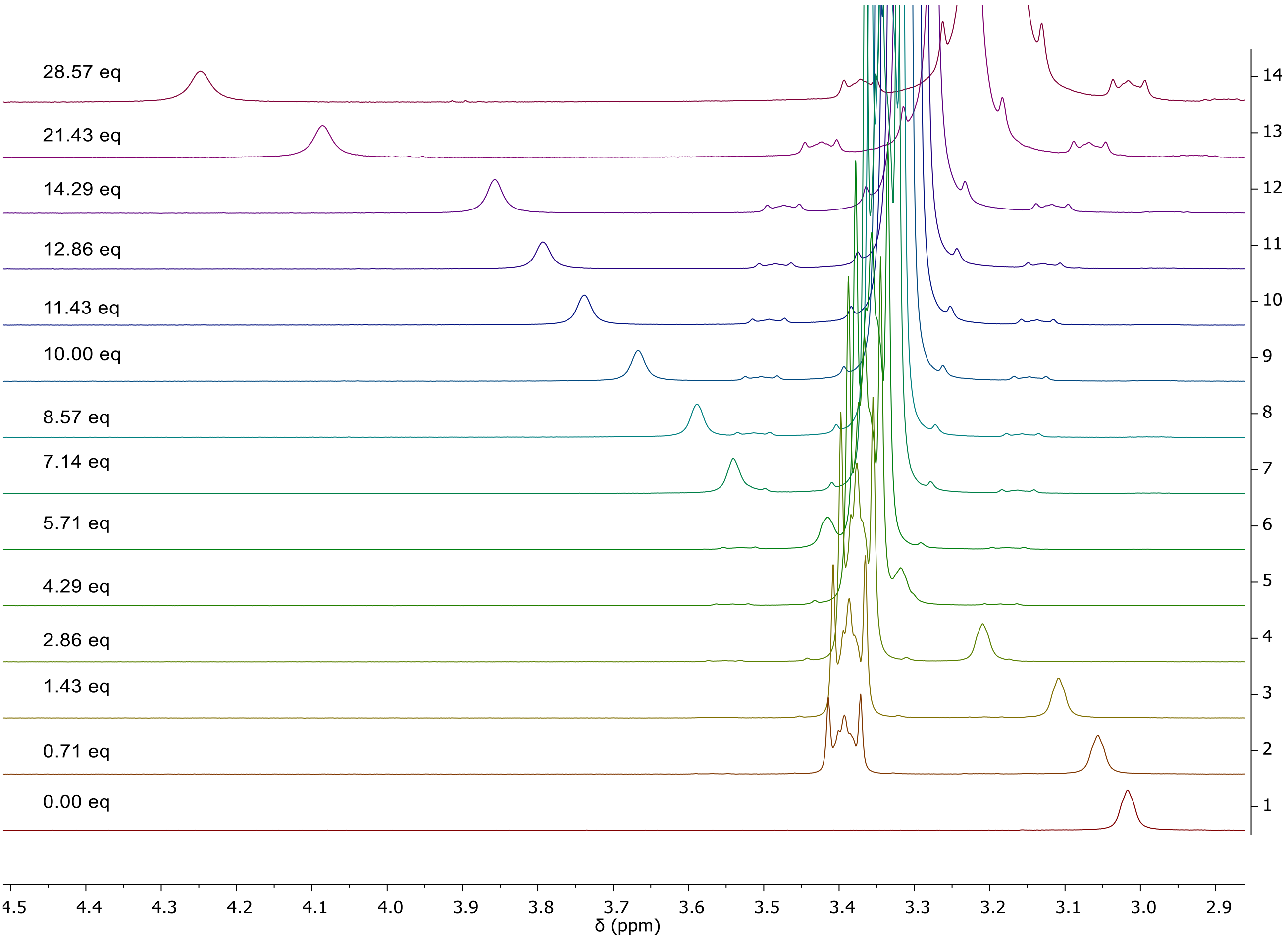

Figure S41: ^1H NMR titration (25 °C, $CDCl_3$, 400 MHz) of **3m** with increasing amounts of TBABr.

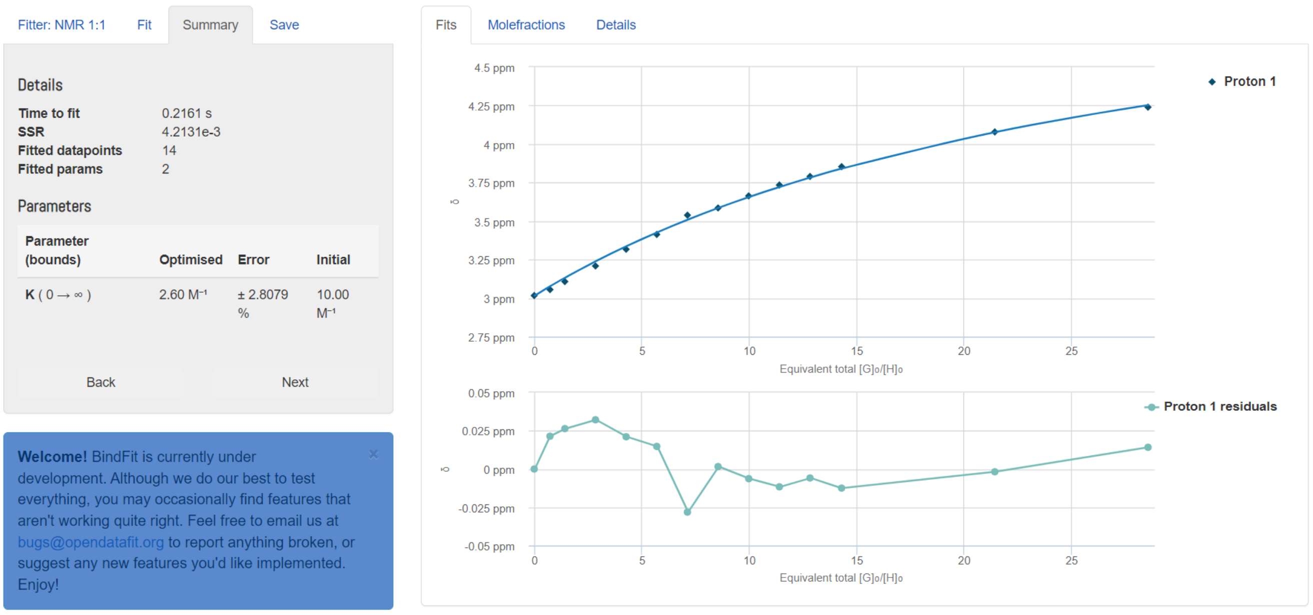

Figure S42: Binding isotherm (1:1 system) fitted to the ^1H NMR chemical shift of the NH protons in **3m** with increasing amounts of TBABr. Graph obtained using the bindfit tool from www.supramolecular.org.[6]

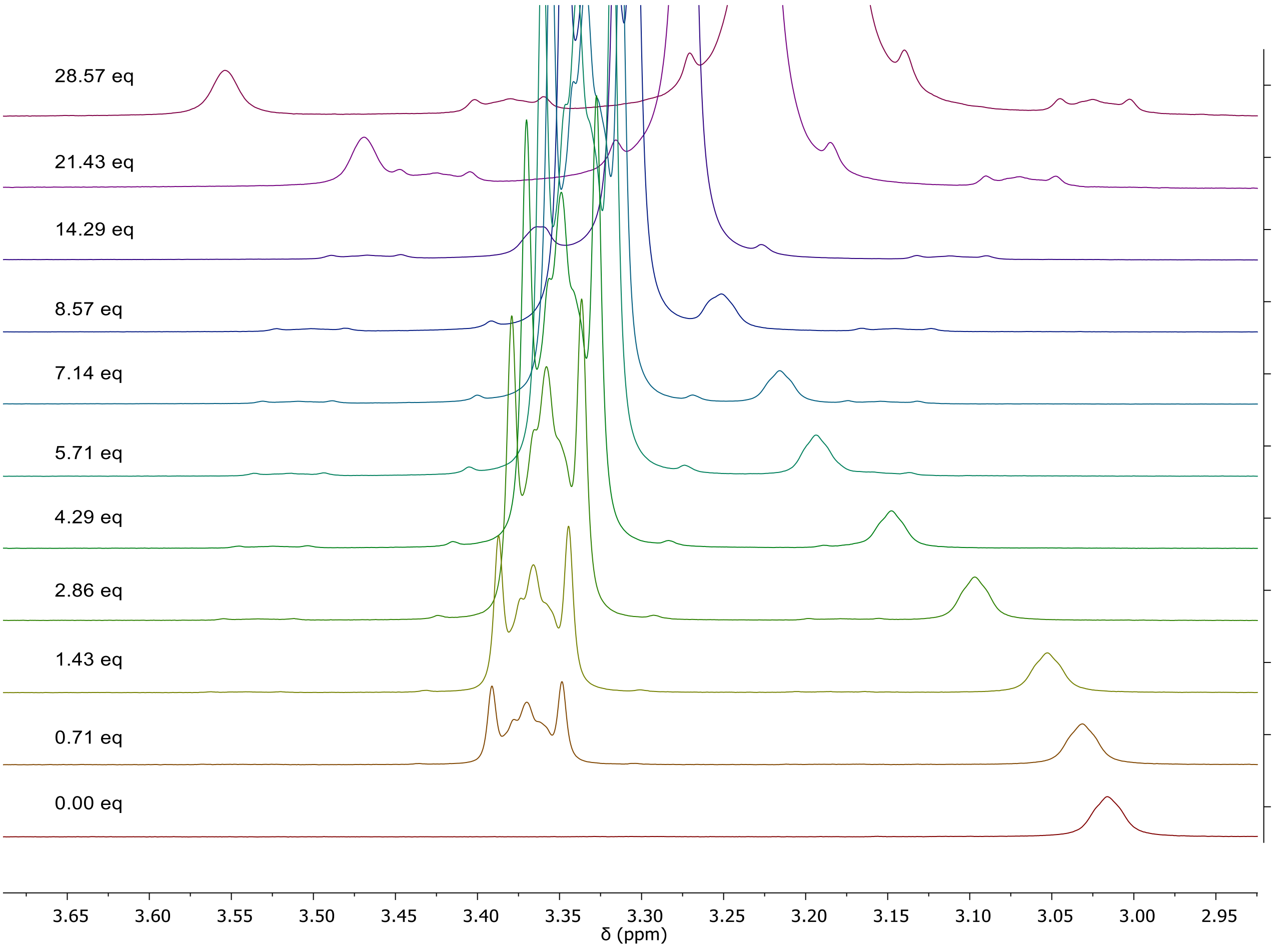

Figure S43: ^1H NMR titration (25 °C, $CDCl_3$, 400 MHz) of **3m** with increasing amounts of TBAI.

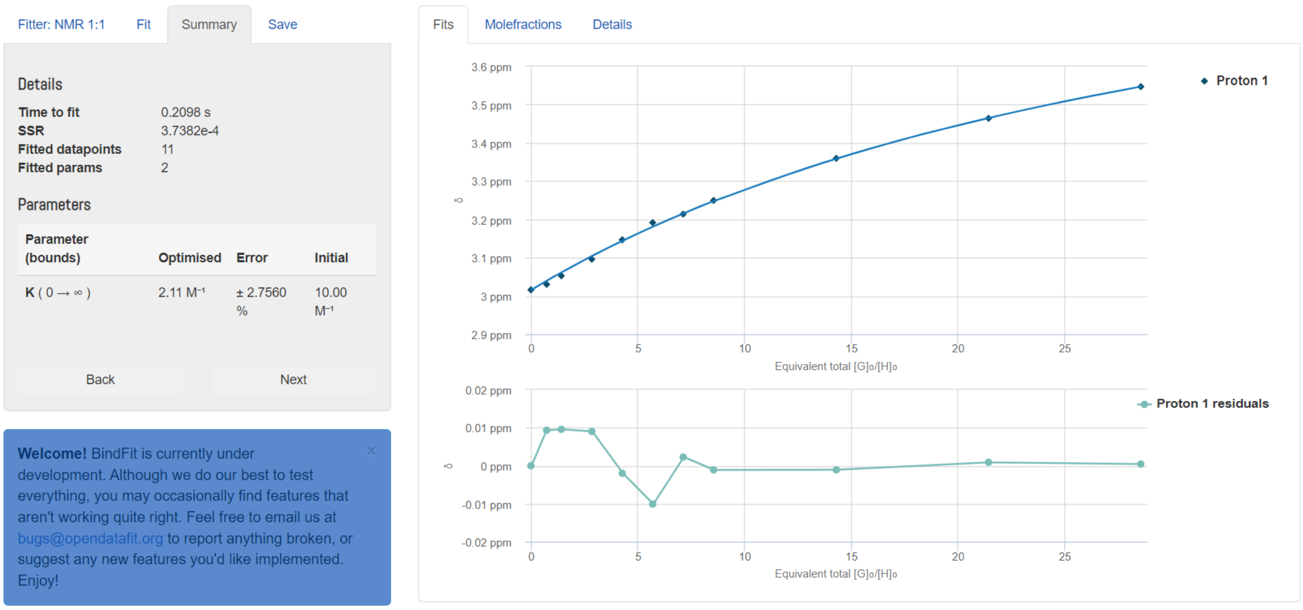

Figure S44: Binding isotherm (1:1 system) fitted to the ^1H NMR chemical shift of the NH protons in **3m** with increasing amounts of TBAI. Graph obtained using the bindfit tool from www.supramolecular.org.[6]

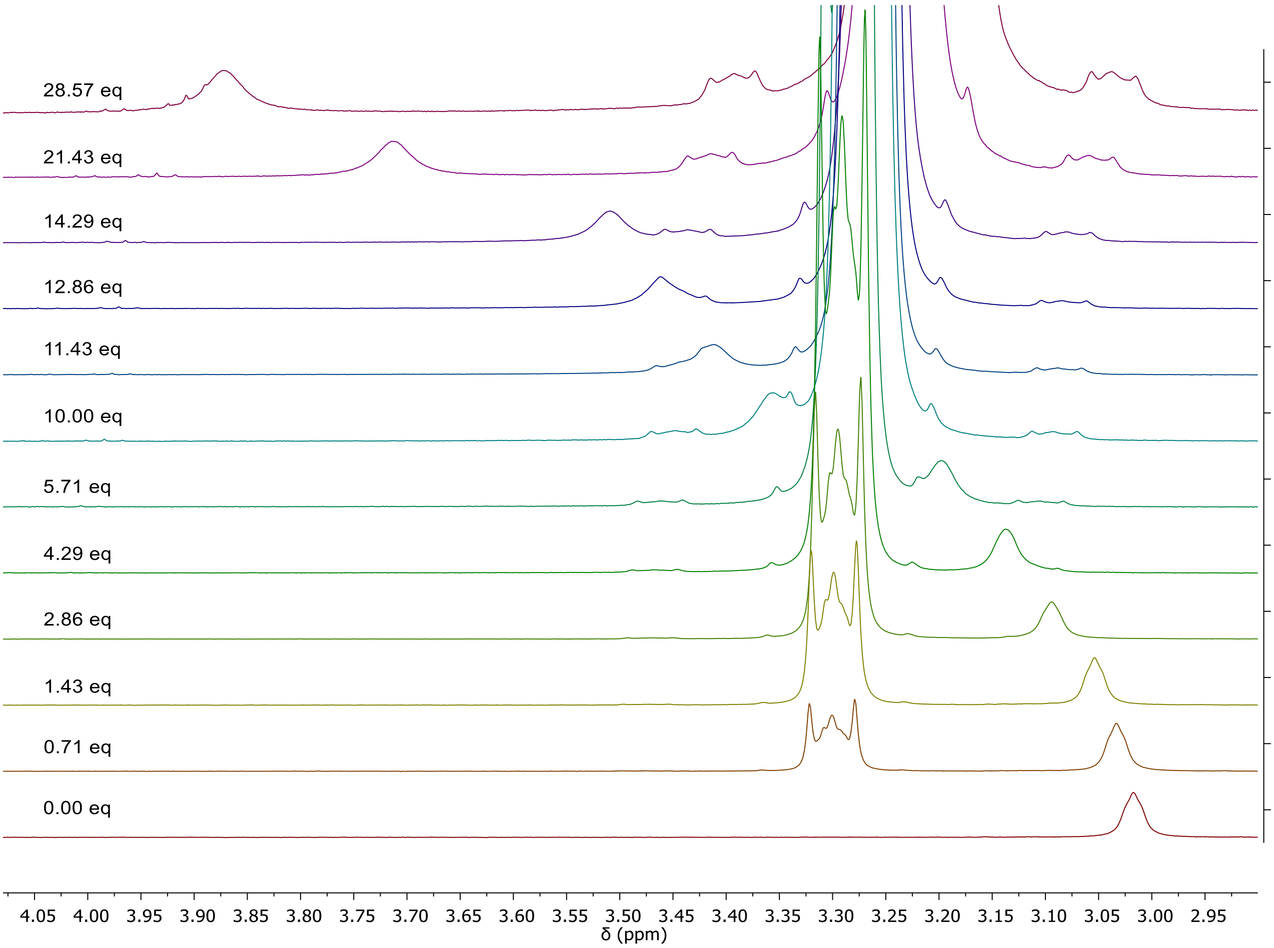

Figure S45: ^1H NMR titration (25 °C, $CDCl_3$, 400 MHz) of **3m** with increasing amounts of $TBAHSO_4$.

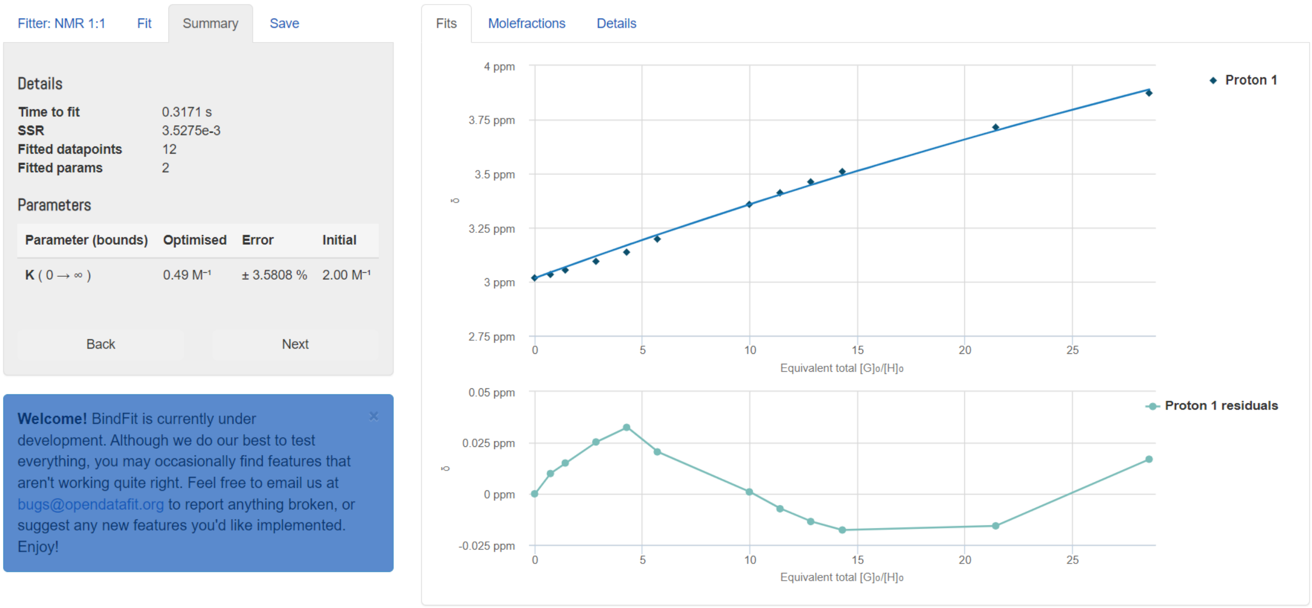

Figure S46: Binding isotherm (1:1 system) fitted to the ^1H NMR chemical shift of the NH protons in **3m** with increasing amounts of $TBAHSO_4$. Graph obtained using the bindfit tool from www.supramolecular.org.[6]

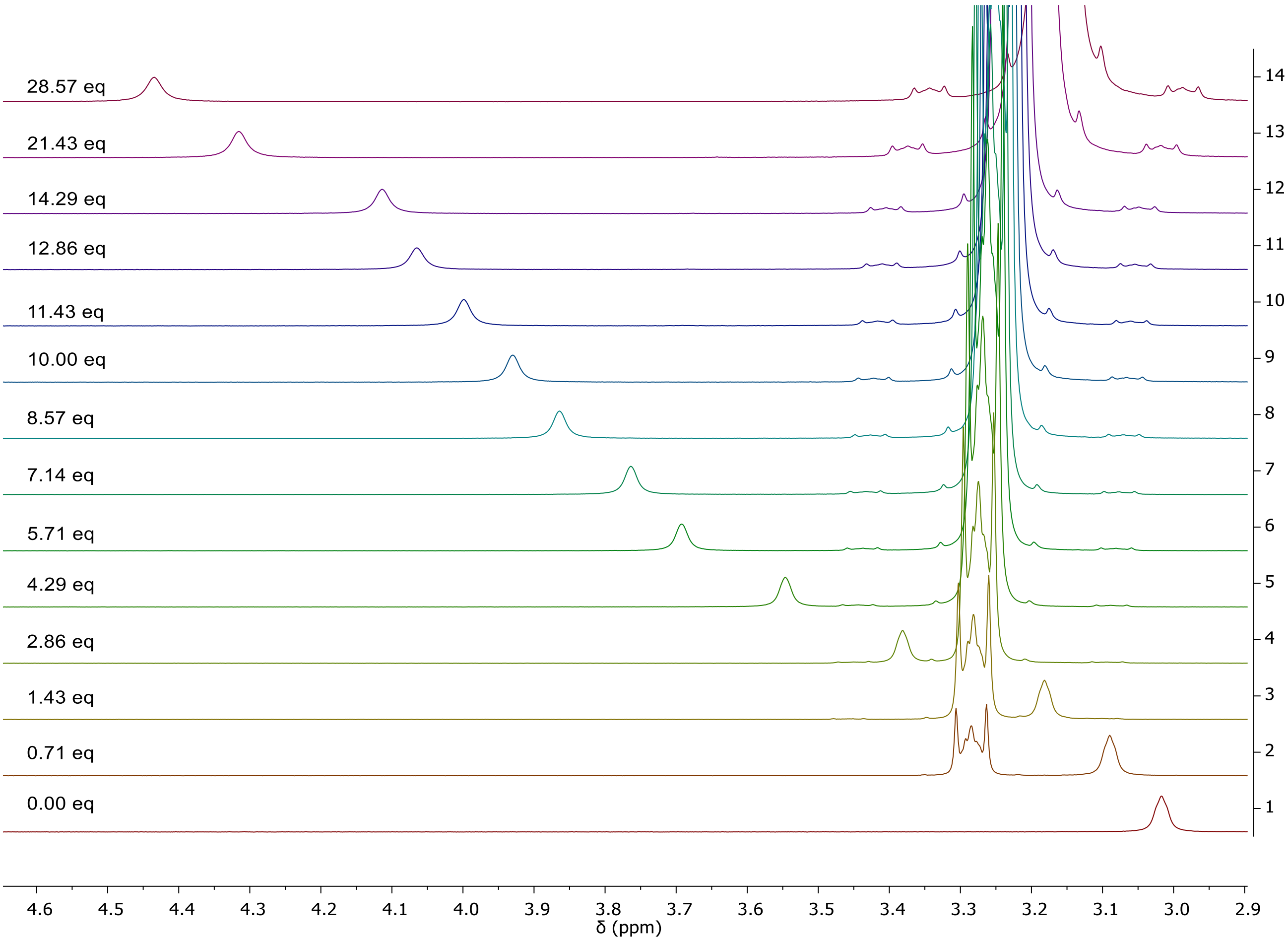

Figure S47: ^{1}H NMR titration (25 °C, $CDCl_3$, 400 MHz) of **3m** with increasing amounts of $TBANO_3$.

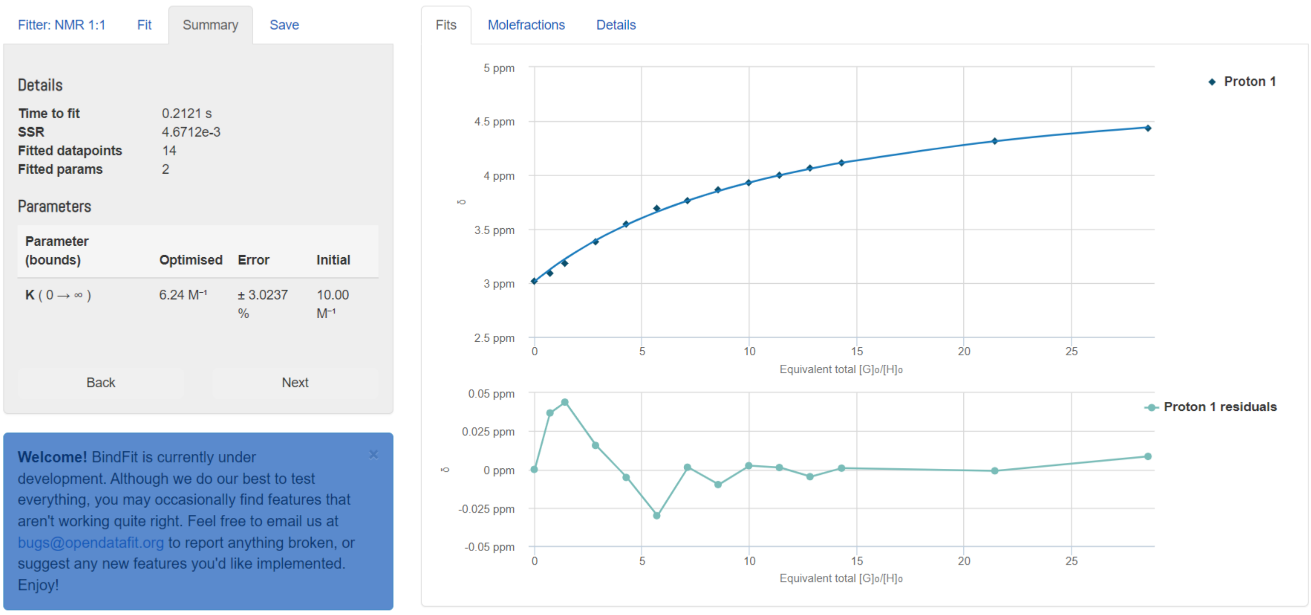

Figure S48: Binding isotherm (1:1 system) fitted to the ^{1}H NMR chemical shift of the NH protons in **3m** with increasing amounts of $TBANO_3$. Graph obtained using the bindfit tool from www.supramolecular.org.[6]

4.3 Monomeric Se (4^m)

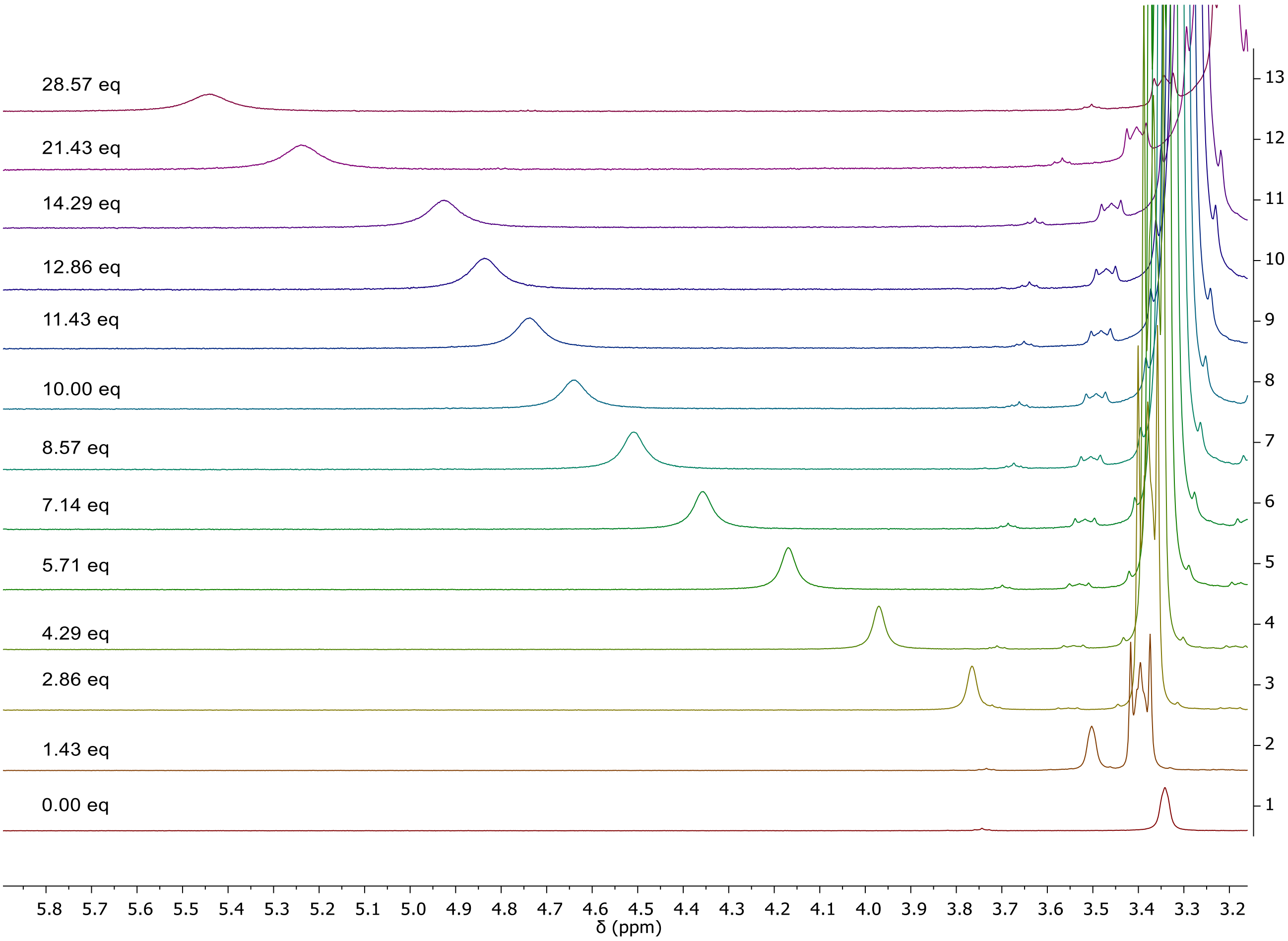

Figure S49: ^{1}H NMR titration (25 °C, $CDCl_3$, 400 MHz) of $\mathbf{4^m}$ with increasing amounts of TBACl.

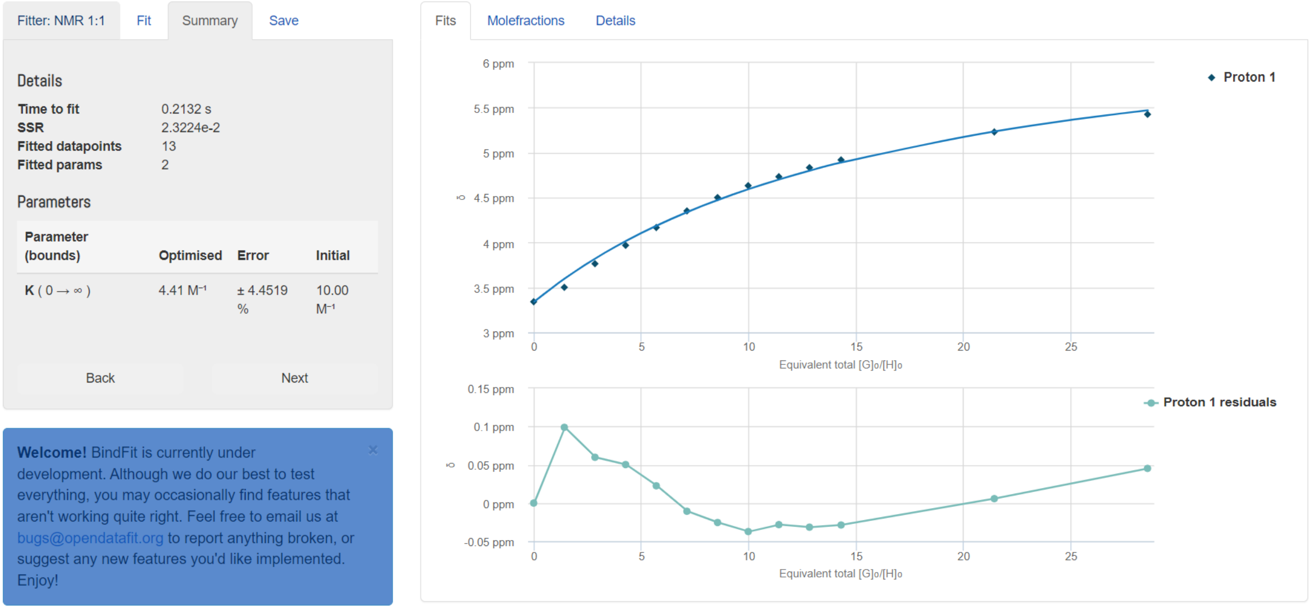

Figure S50: Binding isotherm (1:1 system) fitted to the ^{1}H NMR chemical shift of the NH protons in $\mathbf{4^m}$ with increasing amounts of TBACl. Graph obtained using the bindfit tool from www.supramolecular.org.[6]

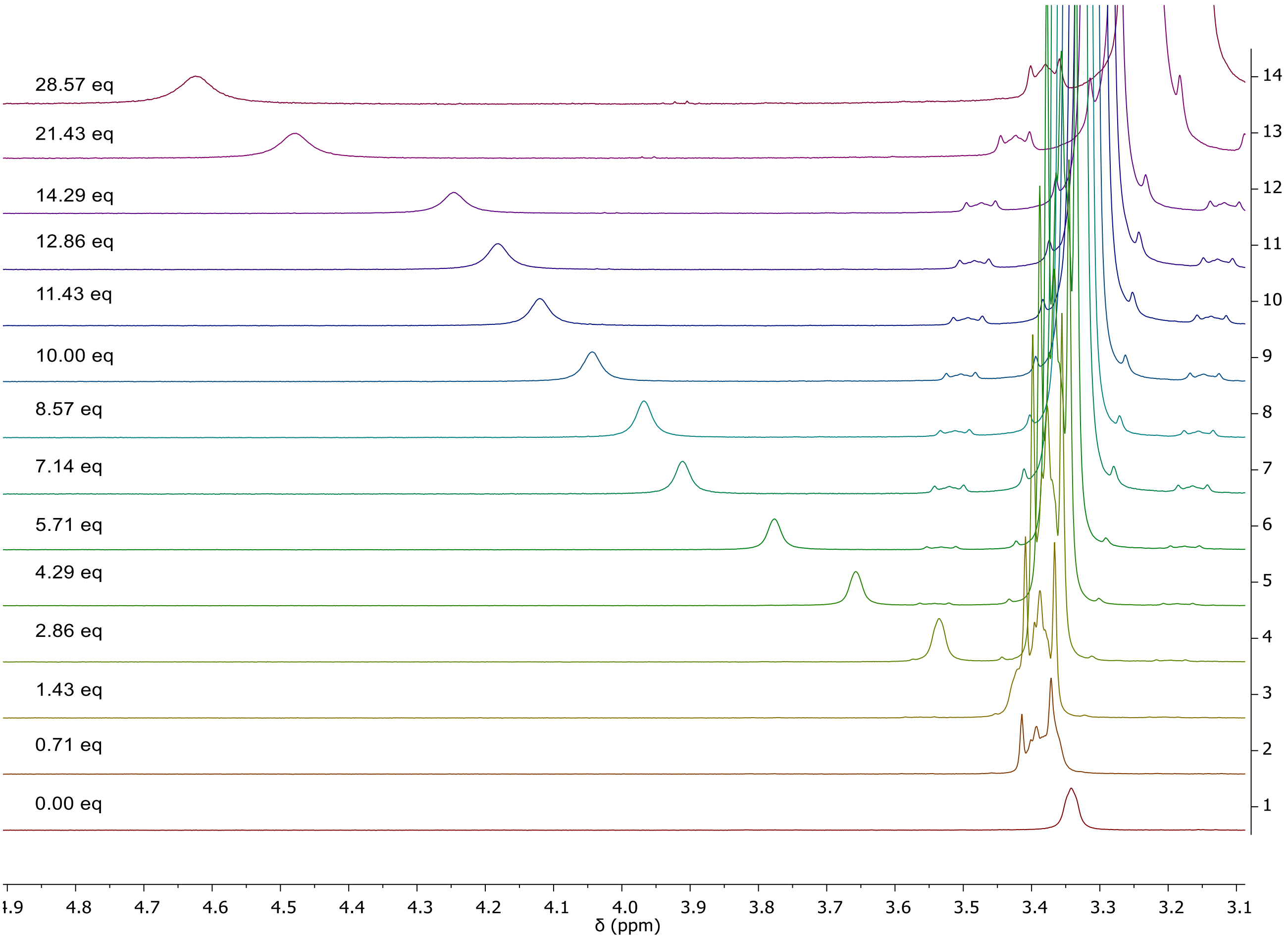

Figure S51: ^{1}H NMR titration (25 °C, $CDCl_3$, 400 MHz) of **4m** with increasing amounts of TBABr.

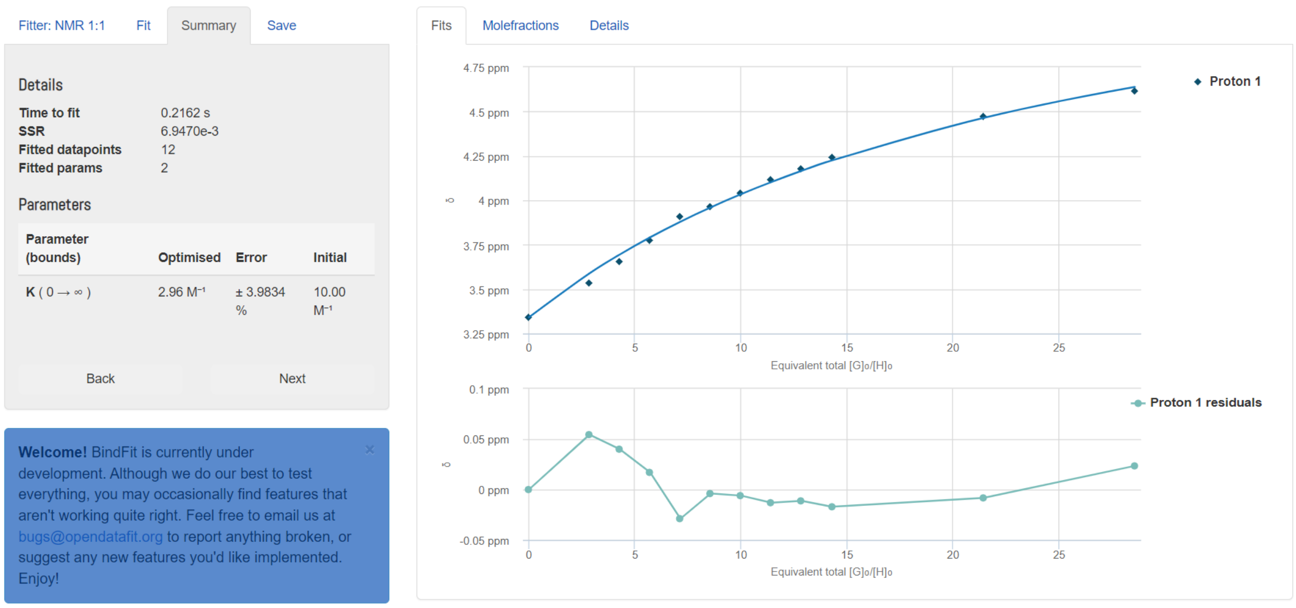

Figure S52: Binding isotherm (1:1 system) fitted to the ^{1}H NMR chemical shift of the NH protons in **4m** with increasing amounts of TBABr. Graph obtained using the bindfit tool from www.supramolecular.org.[6]

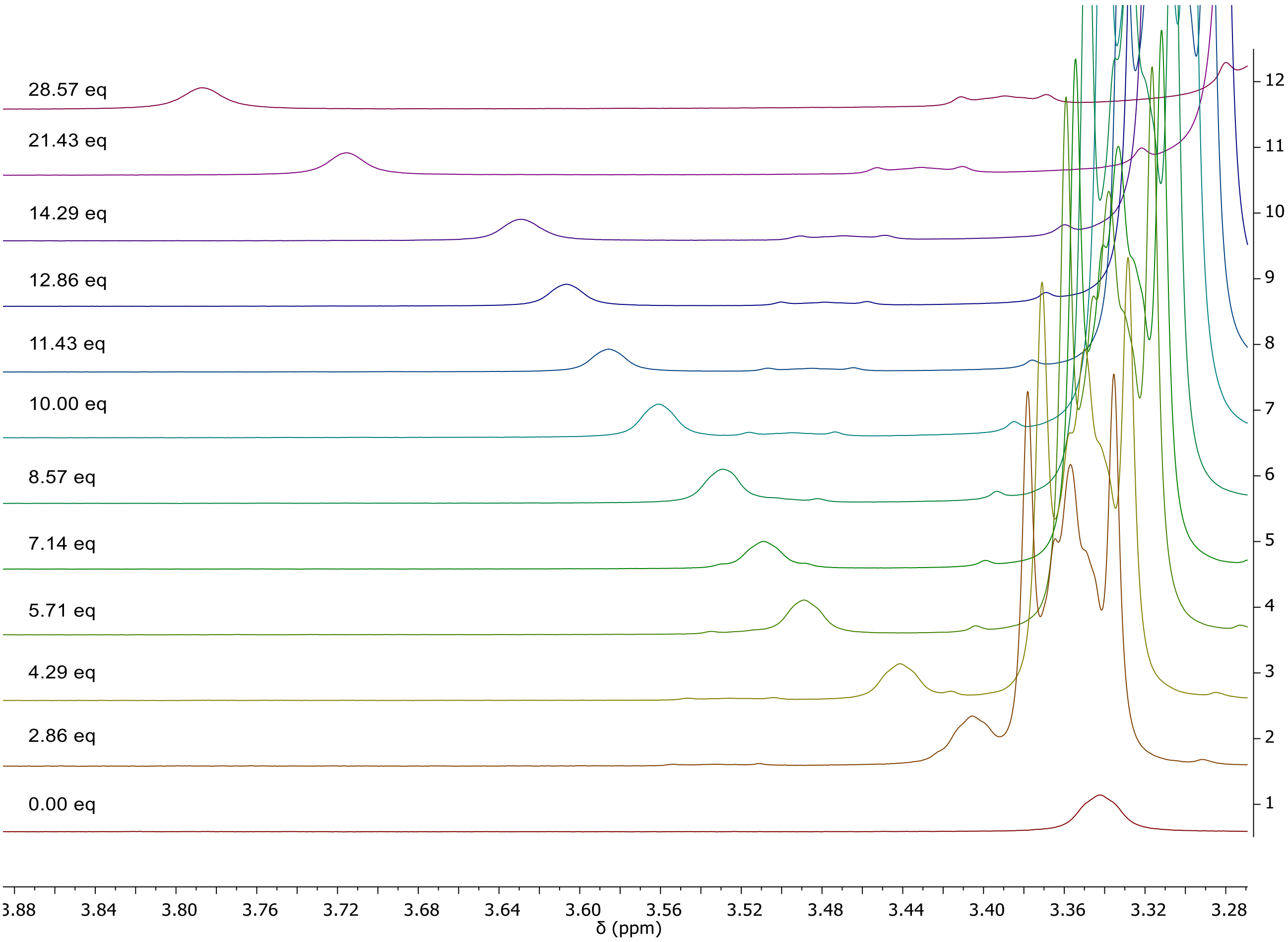

Figure S53: ^{1}H NMR titration (25 °C, $CDCl_3$, 400 MHz) of 4**m** with increasing amounts of TBAI.

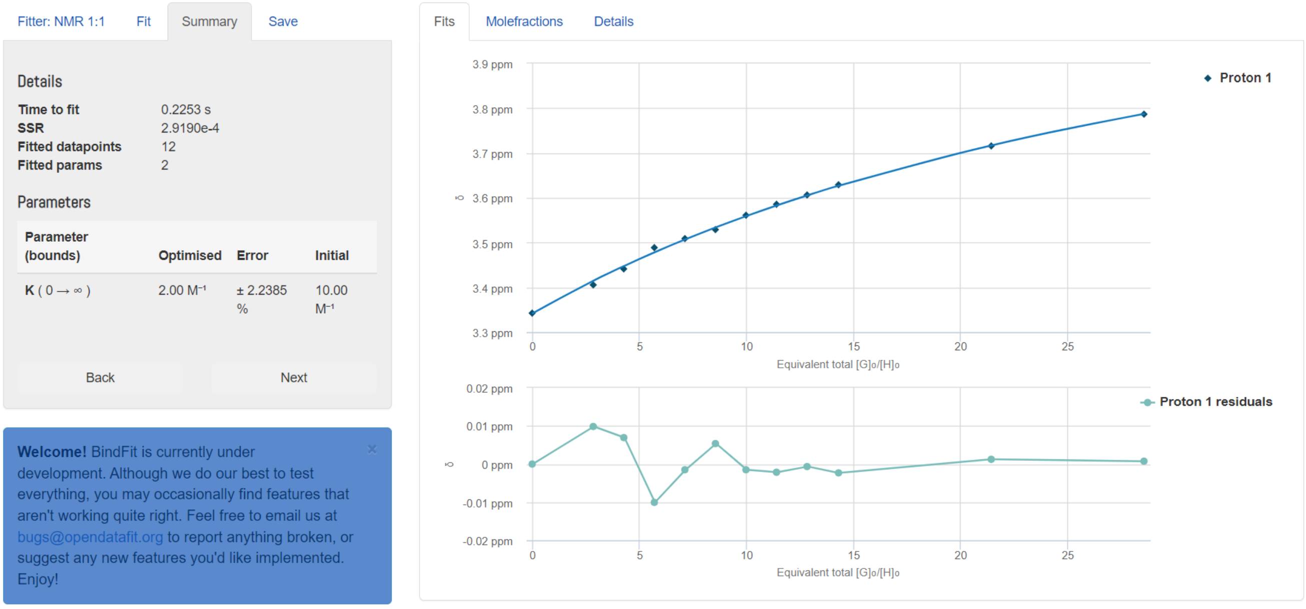

Figure S54: Binding isotherm (1:1 system) fitted to the ^{1}H NMR chemical shift of the NH protons in **4m** with increasing amounts of TBAI. Graph obtained using the bindfit tool from www.supramolecular.org.[6]

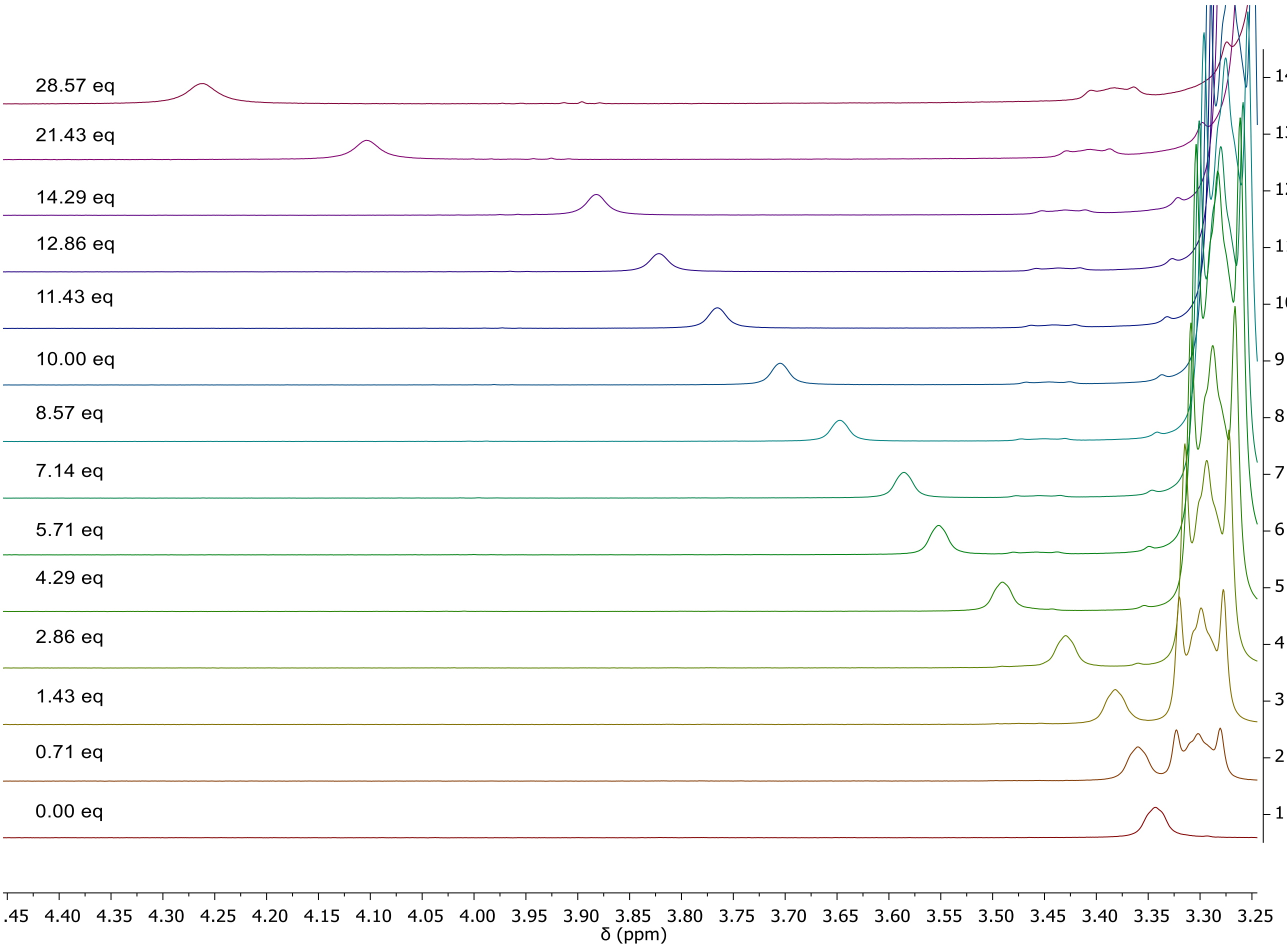

Figure S55: ^{1}H NMR titration (25 °C, $CDCl_3$, 400 MHz) of **4^{m}** with increasing amounts of $TBAHSO_4$.

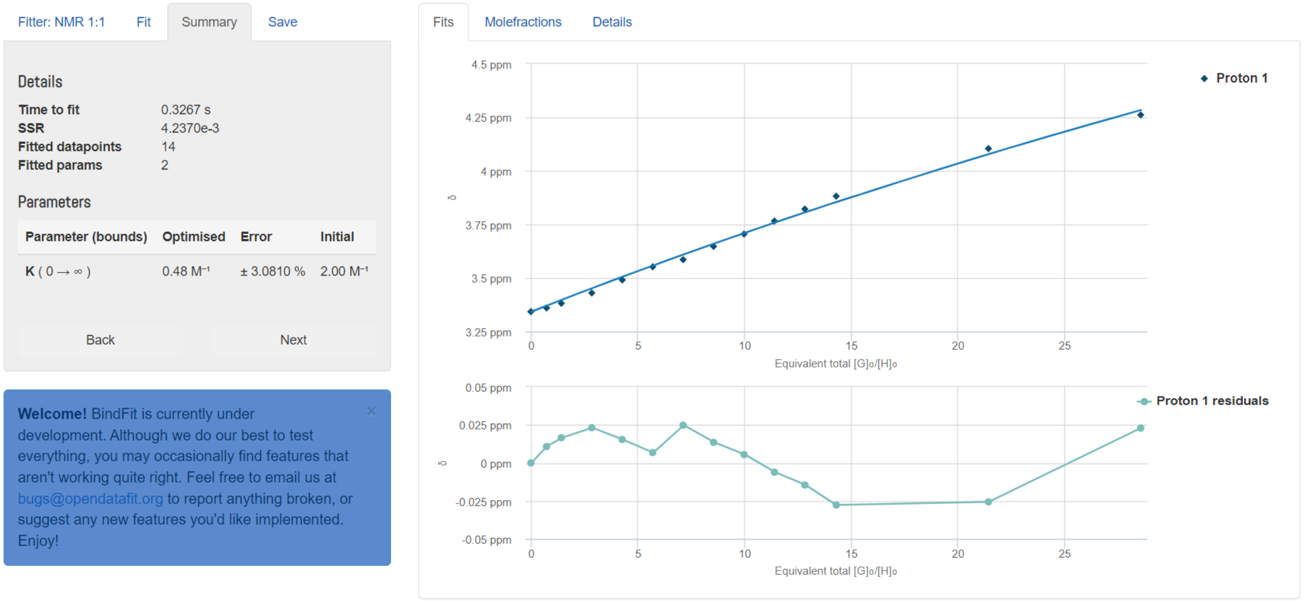

Figure S56: Binding isotherm (1:1 system) fitted to the ^{1}H NMR chemical shift of the NH protons in **4^{m}** with increasing amounts of $TBAHSO_4$. Graph obtained using the bindfit tool from www.supramolecular.org.[6]

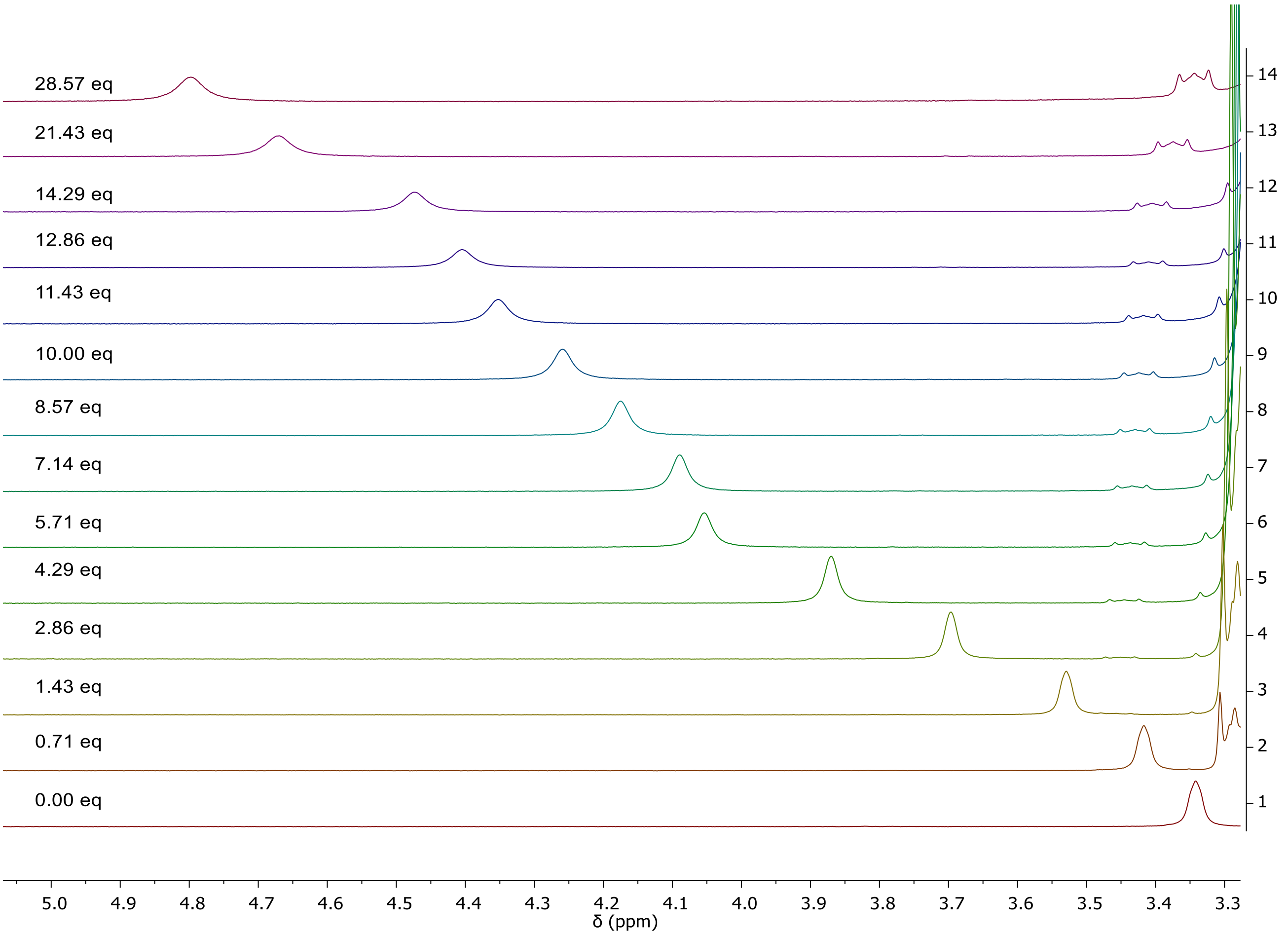

Figure S57: ^{1}H NMR titration (25 °C, $CDCl_3$, 400 MHz) of **4^m** with increasing amounts of $TBANO_3$.

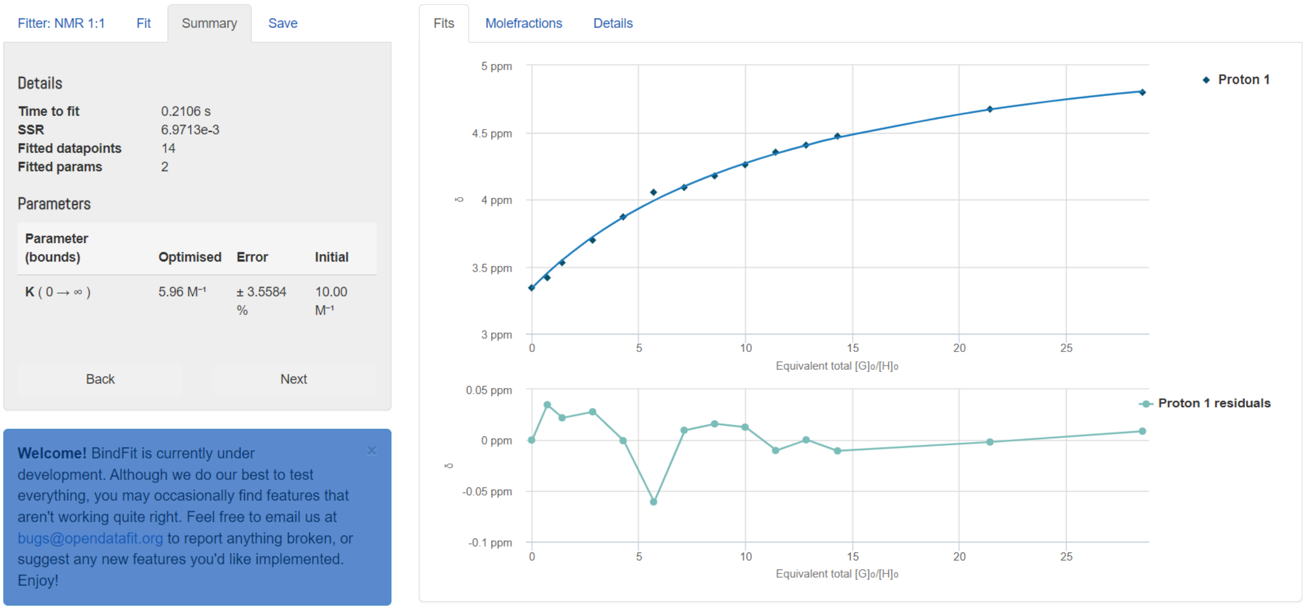

Figure S58: Binding isotherm (1:1 system) fitted to the ^{1}H NMR chemical shift of the NH protons in **4^m** with increasing amounts of $TBANO_3$. Graph obtained using the bindfit tool from www.supramolecular.org.[6]

5. X-ray analyses

Crystallographic Analyses. Diffraction-quality crystals were obtained via methods presented in the experimental section. Diffraction intensity data were measured at 100 K with a Bruker Kappa diffractometer equipped with a CCD detector, employing either Mo K_{α} (λ = 0.71073 Å) radiation, with the SMART suite of programs.[7] Data were processed with SAINT and SADABS.[8] Structural solution and refinement were carried out with the SHELXTL suite of programs.[9] In general, non-hydrogen atoms with occupancies greater than 0.5 were refined anisotropically. Carbon-bound hydrogen atoms were included in idealised positions and refined using a riding model. Disorder was modelled using standard crystallographic methods including constraints, restraints and rigid bodies where necessary. Structures **3**$\subset I^-$ **a**nd **3**$\subset NO_3^-$ displayed less than ideal diffraction properties with broad reflections and a significant amount of diffuse scatter. The structures are both 50 % disordered around a special position. Accordingly, both the cation and the tertiary butyl groups were only modelled isotropically and a number of bond length restraints were required. Despite these limitations the connectivity is unambiguous.

**Table S3:** X-ray data of compounds **2**, **2**^{Chloroform}, **3**⊂Acetonitrile, **3**⊂DMSO and **3**⊂Cl$^-$.					
	**2**	**2**^{Chloroform}	**3**⊂Acetonitrile	**3**⊂DMSO	**3**⊂Cl$^-$
**Chemical formula**	$C_{24}H_{57}N_7O_4P_4$	$C_{27}H_{60}Cl_9N_7O_4P_4$	$C_{26}H_{60}N_8O_0P_4S_4$	$C_{26}H_{63}N_7OP_4S_5$	$C_{41}H_{94}Cl_4N_8P_4S_4$
**Formula weight**	631.64	989.75	736.94	774.01	1093.16
**Crystal system**	monoclinic	triclinic	monoclinic	orthorhombic	monoclinic
**Space group**	P 1 21/n 1	P -1	P 1 21/c 1	P 21 21 21	P 1 21/c 1
**a/ Å**	12.9259(6)	13.4855(10)	15.5088(5)	12.6598(5)	17.5970(9)
**b /Å**	18.4871(8)	13.6520(9)	12.7754(5)	16.2991(7)	18.0517(9)
**c/ Å**	14.7919(7)	13.9559(10)	20.2139(8)	20.2513(8)	20.0538(10)
**α/º**	90	82.719	90	90	90
**β/º**	101.087(2)	76.766	99.3400(10)	90	113.114(2)
**γ/º**	90	69.305	90	90	90
**Volume/ Å^3**	3468.73(3)	2336.8(3)	3951.9(3)	4178.7(3)	5858.8(5)
**Z**	4	2	4	4	4
**Λ (Å)**	0.71073	0.71073	0.71073	0.71073	0.71073
**ρ (Calc)/ Mg.m^{-3}**	1.210	1.407	1.239	1.230	1.239
**Absorp. Coeff./ mm^{-1}**	0.256	0.715	0.431	0.460	0.489
**F(000)**	1368	1032	1584	1664	2352
**Crystal Size/ mm^3**	0.120 x 0.200 x 0.220	0.200 x 0.200 x 0.400	0.250 x 0.350 x 0.700	0.200 x 0.400 x 0.600	0.500 x 0.500 x 0.600
**Θ range/ º**	2.33 to 32.59	1.50 to 25.15	1.33 to 26.43	1.60 to 25.06	1.69 to 25.09
**Index range**	-17<=h<=16<br>-24<=k<=24<br>-19<=l<=18	-16<=h<=16<br>-15<=k<=16<br>-16<=l<=16	-16<=h<=19<br>-15<=k<=15<br>-25<=l<=25	-11<=h<=15<br>-19<=k<=19<br>-24<=l<=24	-20<=h<=20<br>-20<=k<=21<br>-23<=l<=23
**Refl. collected**	36709	37573	67752	60992	95289
**Indep. Refns. (R_{int})**	8595 (0.0842)	8334 (0.0414)	8107 (0.0423)	7402 (0.0638)	10371 (0.0446)
**Completeness to Θ =**	99.5%	99.5%	99.6%	99.8%	99.7%
**Absorp. Corr.**	Multi-Scan	Multi-Scan	Multi-Scan	Multi-Scan	Multi-Scan
**Max., min., transmission**	0.9700, 0.9460	0.8700, 0.7630	0.9000, 0.7520	0.9140, 0.7700	0.7920, 0.7580
**Refinement Method**	Full-matrix least-squares on F^2	Full-matrix least-squares on F^2	Full-matrix least-squares on F^2	Full-matrix least-squares on F^2	Full-matrix least-squares on F^2
**Data/ restraint/parameters**	8595 / 0 / 370	8334 / 0 / 478	8107 / 0 / 398	7402 / 0 / 408	10371 / 571 / 722
**Goodness-of-fit on F^2**	1.022	1.005	1.021	1.066	1.099
**Final R indices [I>2σ(I)]**	R1 = 0.0577<br>wR2 = 0.1291	R1 = 0.0432<br>wR2 = 0.1049	R1 = 0.0274<br>wR2 = 0.0646	R1 = 0.0335<br>wR2 = 0.0601	R1 = 0.0345<br>wR2 = 0.0703
**R indices (all data)**	R1 = 0.0923<br>wR2 = 0.1484	R1 = 0.0649<br>wR2 = 0.1212	R1 = 0.0367<br>wR2 = 0.0694	R1 = 0.0503,<br>wR2 = 0.0665	R1 = 0.0495<br>wR2 = 0.0807
**Largest diff. peak and hole/ e. Å^{-3}**	0.66, -0.88	1.108, -0.948	0.395, -0.361	0.432, -0.291	0.724, -0.465
**Temperature/ K**	100(2)	100(2)	100(2)	100(2)	100(2)

Table S4: X-ray data of compounds **3**⊂Br⁻, **3**⊂I⁻, **3**⊂NO_3^-, **4a** and **4ᵐ**⊂DMSO.

	**3**⊂Br^-	**3**⊂I^-	**3**⊂NO_3^-	**4a**	**4**^{m}⊂DMSO
**Chemical formula**	$C_{40}H_{93}BrN_8P_4S_4$	$C_{40}H_{93}IN_8P_4S_4$	$C_{40}H_{93}N_9O_3P_4S_4$	$C_{19}H_{38}N_4P_2Se_2$	$C_{18}H_{44}N_4OP_2S_3$
**Formula weight**	1018.25	1065.24	1000.35	542.39	490.69
**Crystal system**	orthorhombic	orthorhombic	orthorhombic	monoclinic	monoclinic
**Space group**	P m n 21	P m n 21	P m n 21	P 1 21/c 1	P 1 21/n 1
**a/ Å**	14.3496(13)	14.6030(10)	14.7504(16)	10.3651(3)	10.1335(5)
**b /Å**	10.2704(9)	10.0734(8)	10.1012(11)	11.9271(3)	16.2429(5)
**c/ Å**	18.6772(16)	18.8922(11)	18.6354(17)	21.2421(4)	16.3641(8)
**α/º**	90	90	90	90	90
**β/º**	90	90	90	99.0540(11)	91.095(2)
**γ/º**	90	90	90	90	90
**Volume/ Å^3**	2752.6(4)	2779.1(3)	2776.6(5)	2593.35(11)	2693.0(2)
**Z**	2	2	2	4	4
**Λ (Å)**	0.71073	0.71073	0.71073	0.71073	0.71073
**ρ (Calc)/ Mg.m^{-3}**	1.229	1.273	1.197	1.389	1.120
**Absorp. Coeff./ mm^{-1}**	1.049	0.876	0.328	2.986	0.410
**F(000)**	1096	1132	1088	1112	1064
**Crystal Size/ mm^3**	0.240 x 0.240 x 0.250	0.100 x 0.200 x 0.250	0.020 x 0.120 x 0.320	0.020 x 0.140 x 0.280	0.050 x 0.300 x 0.600
**Θ range/ º**	1.79 to 30.68	1.76 to 25.05	1.75 to 25.33	2.55 to 32.57	1.77 to 30.87
**Index range**	-20<=h<=20<br>-14<=k<=14<br>-26<=l<=25	-17<=h<=17<br>-12<=k<=11<br>-22<=l<=18	-16<=h<=17<br>-12<=k<=11<br>-22<=l<=22	-15<=h<=15<br>-18<=k<=14<br>-31<=l<=32	-14<=h<=14<br>-23<=k<=15<br>-23<=l<=23
**Refl. collected**	51974	21338	18375	39617	31593
**Indep. Refns. (R_{int})**	8690 (0.1536)	4802 (0.0660)	4886 (0.0973)	9418 (0.0865)	8410 (0.0923)
**Completeness to Θ =**	99.6%	99.5%	99.2%	99.9%	98.9%
**Absorp. Corr.**	Multi-Scan	Multi-Scan	Multi-Scan	Multi-Scan	Multi-Scan
**Max., min., transmission**	0.7870, 0.7790	0.92, 0.81	0.94, 0.45	0.9430, 0.4890	0.9800, 0.7910
**Refinement Method**	Full-matrix least-squares on F^2	Full-matrix least-squares on F^2	Full-matrix least-squares on F^2	Full-matrix least-squares on F^2	Full-matrix least-squares on F^2
**Data/ restraint/parameters**	8690 / 580 / 536	4802 / 176 / 330	4886 / 96 / 337	9418 / 267 / 295	8410 / 114 / 306
**Goodness-of-fit on F^2**	1.029	1.061	1.1079	1.013	1.117
**Final R indices [I>2σ(I)]**	R1 = 0.0813<br>wR2 = 0.1897	R1 = 0.0639<br>wR2 = 0.1451	R1 = 0.1105<br>wR2 = 0.2865	R1 = 0.0475<br>wR2 = 0.0921	R1 = 0.0629<br>wR2 = 0.1113
**R indices (all data)**	R1 = 0.2184<br>wR2 = 0.2698	R1 = 0.1039<br>wR2 = 0.1768	R1 = 0.1286<br>wR2 = 0.3151	R1 = 0.0933,<br>wR2 = 0.1104	R1 = 0.1252<br>wR2 = 0.1294
**Largest diff. peak and hole/ e. Å^{-3}**	1.244, -0.548	0.61, -0.58	0.90, -0.59	0.834, -0.890	0.932, -0.506
**Temperature/ K**	100(2)	100(2)	100(2)	100(2)	100(2)

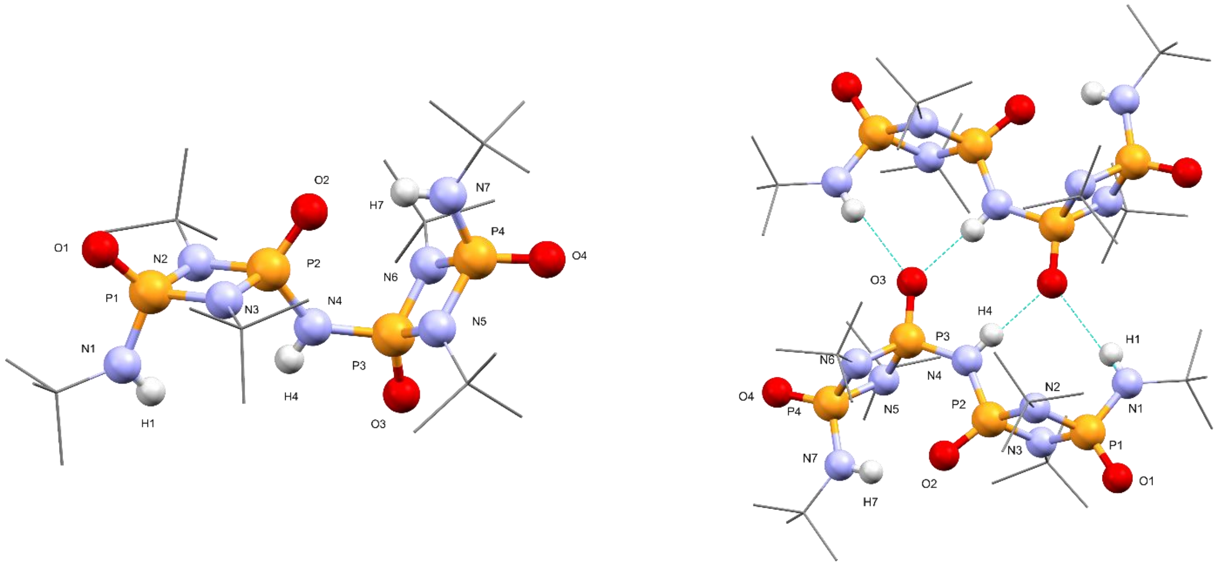

Figure S59. Solid-state structures of **2**. The tert-butyl units are drawn as wireframes in all the graphical representations. H atoms are omitted for clarity. Thermal ellipsoids are set at the 50% probability level. Selected Bond Lengths [Å] and Angles [deg] for **2**: N1-P1 1.633(2), N2-P2 1.671(2), N2-P1 1.685(2), N3-P2 1.659(2), N3-P1 1.686(2), N4-P2 1.670(2), O1-P1 1.468(2), O2-P2 1.464(2), O1-P1-N1 112.13(12), O1-P1-N2 118.83(12), N1-P1-N2 110.44(12), O1-P1-N3 119.81(12), N1-P1-N3, 109.01(12), N2-P1-N3 83.41(11), O2-P2-N3 118.50(12), O2-P2-N4 109.63(11), N3-P2-N4 112.54(12), O2-P2-N2 120.88(11), N3-P2-N2 84.68(11), N4-P2-N2 108.51(11).

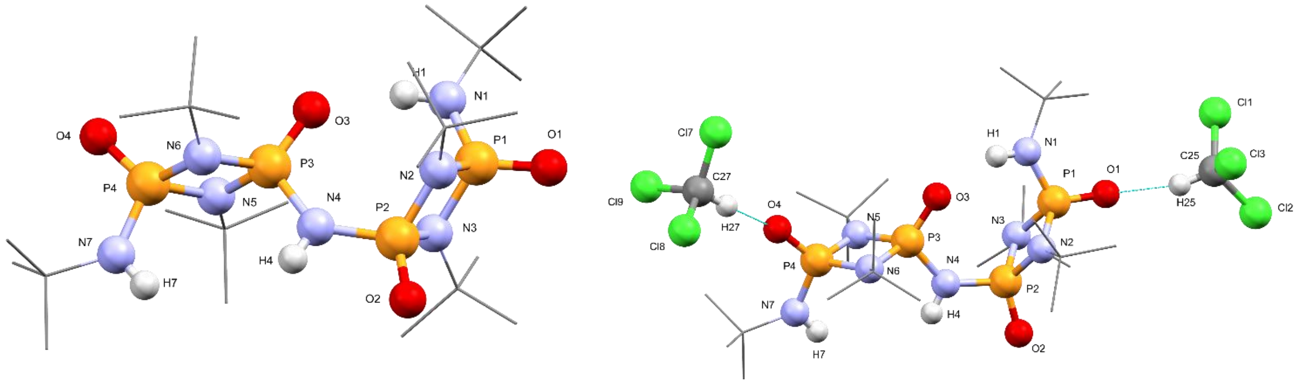

Figure S60. Solid-state structures of **2**$^{\mathbf{Chloroform}}$. The tert-butyl units are drawn as wireframes in all the graphical representations. H atoms are omitted for clarity. Thermal ellipsoids are set at the 50% probability level. Selected Bond Lengths [Å] and Angles [deg] for **2**$^{\mathbf{Chloroform}}$: P1-O1 1.469(2), P1-N1 1.611(2), P1-N2 1.690(2), P1-N3 1.700(2), P2-O2 1.4813(19), P2-N2 1.661(2), P2-N3 1.672(2), P2-N4 1.674(2), O1-P1-N1 113.37(12), O1-P1-N2 119.21(12), N1-P1-N2 108.76(12), O1-P1-N3 116.57(11), N1-P1-N3 111.81(12), N2-P1-N3 83.73(11), O2-P2-N2 120.47(12), O2-P2-N3 118.05(11), N2-P2-N3 85.48(11), O2-P2-N4 106.27(11), N2-P2-N4 112.64(11), N3-P2-N4 113.16(12).

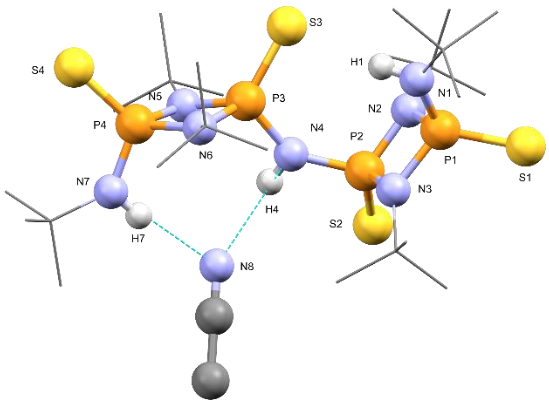

Figure S61. Solid-state structures of **3**⊂Acetonitrile. The tert-butyl units are drawn as wireframes in all the graphical representations. H atoms are omitted for clarity. Thermal ellipsoids are set at the 50% probability level. Selected Bond Lengths [Å] and Angles [deg] for **3**⊂ Acetonitrile: S3-P3 1.9290(5), S4-P4 1.9280(6), P3-N4 1.6793(13), P3-N5 1.6824(13), P3-N6 1.6876(13), P3-P4 2.5190(5), P4-N7 1.6277(13), P4-N5 1.6996(12), P4-N6 1.7089(13), N2-P2-N4 112.17(6), N3-P2-N4 110.20(6), N2-P2-S2 120.63(5), N3-P2-S2 120.23(5), N4-P2-S2 107.32(5), N4-P3-N5 103.36(6), N4-P3-N6 112.42(6), N5-P3-N6 84.17(6), N4-P3-S3 112.77(5), N5-P3-S3 122.44(5), N6-P3-S3 118.16(5), N7-P4-N5 107.50(7), N7-P4-N6 109.67(7), N5-P4-N6 83.01(6), N7-P4-S4 114.52(5), N5-P4-S4 119.31(5), N6-P4-S4 118.62(5).

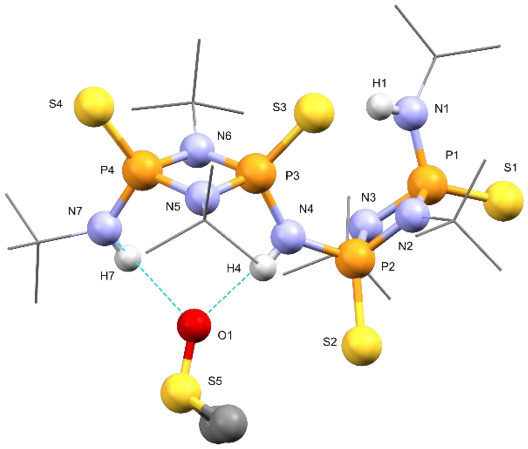

Figure S62. Solid-state structures of **3**⊂DMSO. The tert-butyl units are drawn as wireframes in all the graphical representations. H atoms are omitted for clarity. Thermal ellipsoids are set at the 50% probability level. Selected Bond Lengths [Å] and Angles [deg] for **3**⊂DMSO: S1-P1 1.9283(14), S2-P2 1.9352(14), P1-N2 1.703(3), P1-N3 1.711(3), P1-P2 2.5056(14), P2-N2 1.668(3), P2-N3 1.685(3), P2-N4 1.686(3), N1-P1-N2 105.09(16), N1-P1-N3 111.07(15), N2-P1-N3 83.38(15), N1-P1-S1 114.63(12), N2-P1-S1 120.44(11), N3-P1-S1 118.00(12), N2-P2-N3 85.28(15), N2-P2-N4 112.57(16), N3-P2-N4 110.11(14), N2-P2-S2 118.97(11), N3-P2-S2 120.23(12), N4-P2-S2 108.18(12).

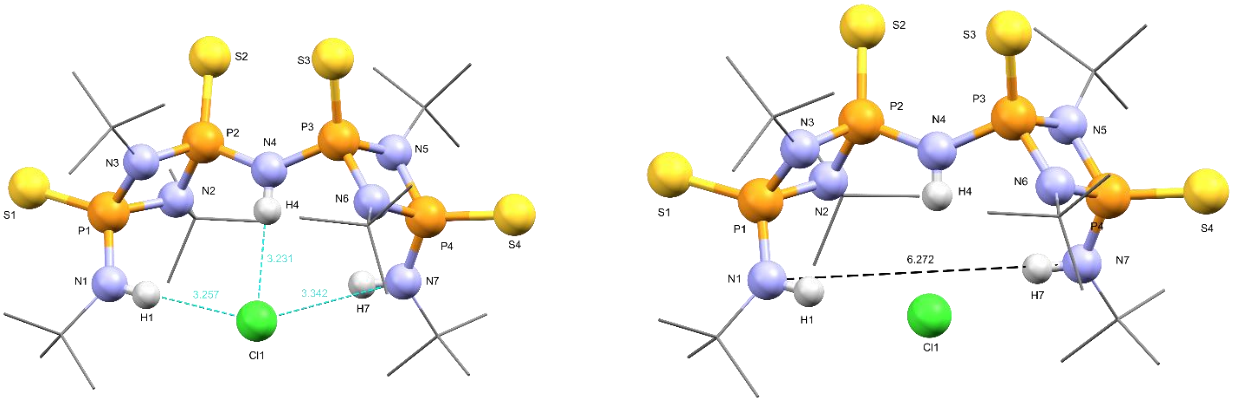

Figure S63. Solid-state structures of **3**⊂Cl$^-$. The tert-butyl units are drawn as wireframes in all the graphical representations. H atoms and solvent molecules are omitted for clarity. Thermal ellipsoids are set at the 50% probability level. Selected Bond Lengths [Å] and Angles [deg] for **3**⊂Cl$^-$: S1-P1 1.9412(8), S2-P2 1.9275(7), S3-P3 1.9237(8), S4-P4 1.9386(8), P1-N1 1.6275(18), P1-N2 1.6912(18), P1-N3 1.6982(18), P2-N4 1.6655(17), P2-N2 1.6811(18), N1-P1-N2 109.02(9), N1-P1-N3 110.38(9), N2-P1-N3 83.12(8), N1-P1-S1 114.32(7), N2-P1-S1 118.88(7), N3-P1-S1 117.19(7), N4-P2-N2 106.09(9), N4-P2-N3 110.15(9), N2-P2-N3 83.80(9), N4-P2-S2 113.59(7), N2-P2-S2 120.45(7), N3-P2-S2 118.93(7).

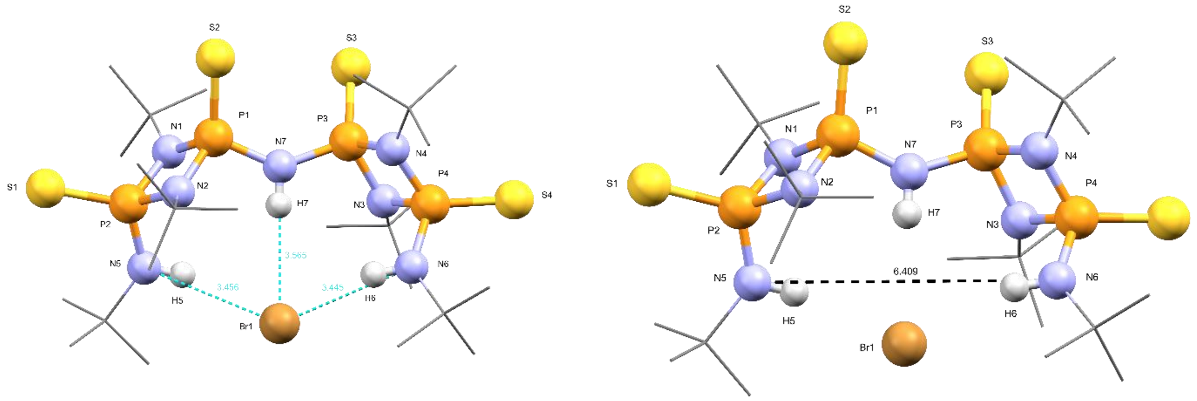

Figure S64. Solid-state structures of **3**⊂Br$^-$. The tert-butyl units are drawn as wireframes in all the graphical representations. H atoms and disorder are omitted for clarity. Thermal ellipsoids are set at the 50% probability level. Selected Bond Lengths [Å] and Angles [deg] for **3**⊂Br$^-$: P1-N7 1.658(16), P1-N2 1.671(15), P1-N1 1.681(19), P1-S2 1.928(7), P1-P2 2.492(14), P2-N5 1.60(3), P2-N2 1.685(14), P2-N1 1.71(2), P2-S1 1.939(7), N7-P1-N2 101.9(7), N7-P1-N1 111.0(9), N2-P1-N1 85.2(8), N7-P1-S2 113.8(5), N2-P1-S2 122.7(6), N1-P1-S2 118.3(8), N5-P2-N2 104.8(11), N5-P2-N1 109.3(9), N2-P2-N1 83.9(10), N5-P2-S1 115.5(10), N2-P2-S1 120.8(6), N1-P2-S1 118.0(12), N5-P2-P1 116.3(6)

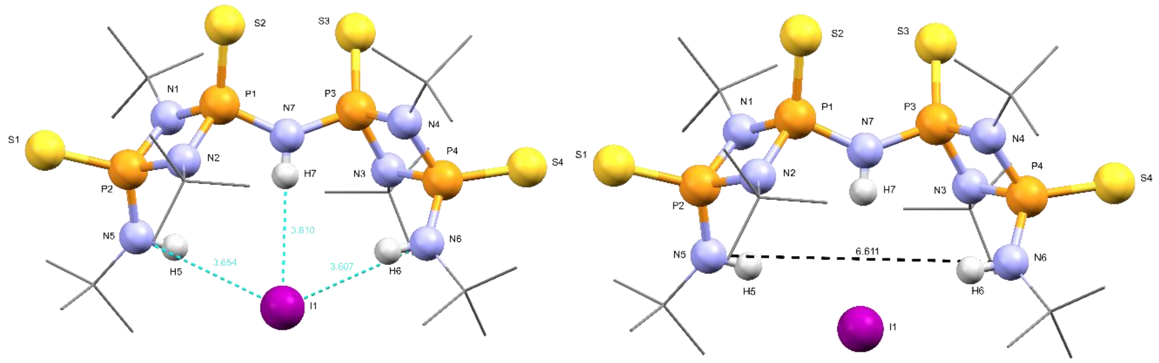

Figure S65. Solid-state structures of **3**$\subset I^-$. The tert-butyl units are drawn as wireframes in all the graphical representations. H atoms and disorder are omitted for clarity. Thermal ellipsoids are set at the 50% probability level. Selected Bond Lengths [Å] and Angles [deg] for **3**$\subset I^-$: P1-N2 1.679(10), P1-N7 1.684(18), P1-N1 1.690(12), P1-S2 1.914(6), P2-N5 1.630(2), P2-N1 1.674(12), P2-N2 1.700(10), P2-S1 1.942(7), N2-P1-N7 103.0(6), N2-P1-N1 83.6(6), N7-P1-N1 111.8(8), N2-P1-S2 122.1(5), N7-P1-S2 114.1(5), N5-P2-N1 111.8(9), N5-P2-N2 106.8(9), N1-P2-N2 83.4(7), N5-P2-S1 113.8(8), N1-P2-S1 117.7(9), N2-P2-S1 119.6(6).

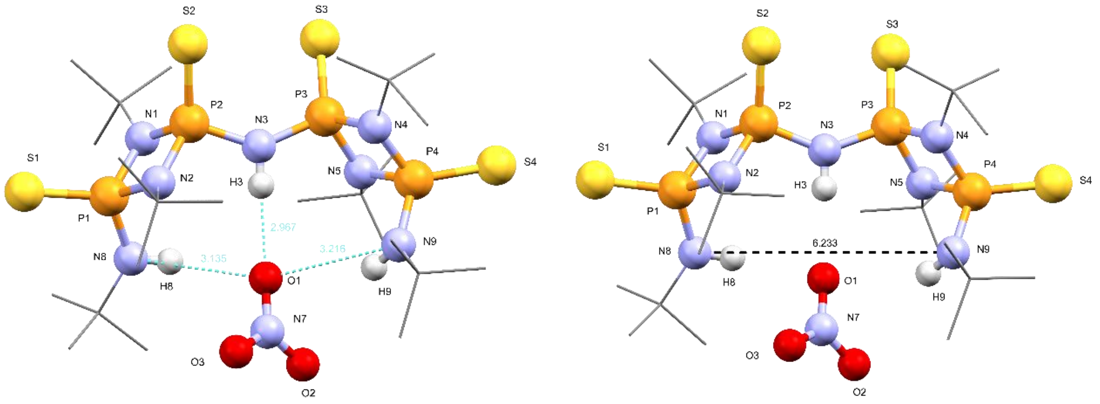

Figure S66. Solid-state structures of **3**$\subset NO_3^-$. The tert-butyl units are drawn as wireframes in all the graphical representations. H atoms and disorder are omitted for clarity. Thermal ellipsoids are set at the 50% probability level. Selected Bond Lengths [Å] and Angles [deg] for **3**$\subset NO_3^-$: P1-S1 1.934(6), P2-S2 1.916(5), P3-S3 1.928(6), P4-S4 1.926(6), N1-P1 1.670(3), N1-P2 1.668(15), N2-P2 1.689(11), N2-P1 1.694(11), N3-P2 1.676(14), N8-P1 1.64(3), O1-N7 1.229(14), N7-O3 1.222(14), N7-O2 1.239(14), P1-N1-P2 96.4(8), P2-N2-P1 94.6(10), N8-P1-N2 106.2(13), N1-P1-N2 84.4(11), N8-P1-S1 114.7(14), N1-P1-S1 118.6(16), N2-P1-S1 119.6(6), O3-N7-O1 122.0(10), O3-N7-O2 119.7(10), O1-N7-O2 118.4(10).

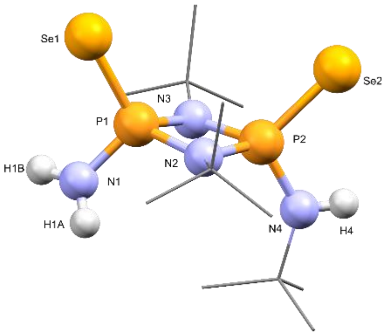

Figure S67. Solid-state structures of **4a**. The tert-butyl units are drawn as wireframes in all the graphical representations. H atoms and solvent molecules are omitted for clarity. Thermal ellipsoids are set at the 50% probability level. Selected Bond Lengths [Å] and Angles [deg] for **4a**: N2-P2 1.686(2), N2-P1 1.695(2), N3-P2 1.691(2), N3-P1 1.696(2), N4-P2 1.623(2), P1-Se1 2.0944(7), P2-Se2 2.1076(7), N1-P1-N2 111.63(12), N1-P1-N3 111.51(12), N2-P1-N3 83.17(11), N1-P1-Se1 108.28(8), N2-P1-Se1 120.26(8), N3-P1-Se1 120.16(8), N4-P2-N2 114.44(12), N4-P2-N3 113.95(12), N2-P2-N3 83.59(11), N4-P2-Se2 106.83(8), N2-P2-Se2 118.52(8), N3-P2-Se2 118.53(8).

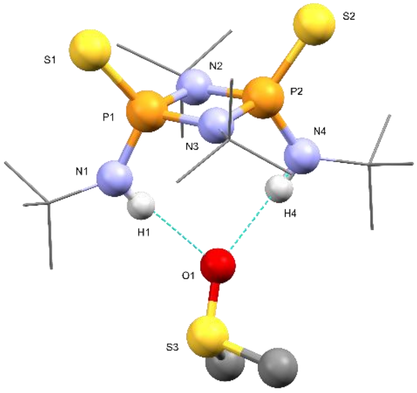

Figure S68. Solid-state structures of **4**^{m}⊂DMSO. The tert-butyl units are drawn as wireframes in all the graphical representations. H atoms and solvent molecules are omitted for clarity. Thermal ellipsoids are set at the 50% probability level. Selected Bond Lengths [Å] and Angles [deg] for **4**^{m}⊂DMSO: S1-P1 1.9347(9), S2-P2 1.9316(10), P1-N1 1.628(2), P1-N2 1.689(2), P1-N3 1.690(2), P1-P2 2.5256(9), P2-N4 1.631(2), P2-N3 1.686(2), P2-N2 1.689(2), N1-P1-N2 110.24(12), N1-P1-N3 108.49(11), N2-P1-N3 83.06(10), N1-P1-S1 114.45(9), N2-P1-S1 117.97(8), N3-P1-S1 118.58(9).

6. Theoretical Studies

Calculations were performed with Gaussian 16 package (DFT),[10] using the Becke Three-Parameter functional with the non-local correlation by Perdew and Wang (B3PW91)[11] and the D3 version of Grimme's dispersion with Becke-Johnson damping (GD3BJ).[12] H, C, P, N, O, S atoms were represented with the 6-311G(d,p)[13] basis set as implemented in Gaussian 16, while Cl, I, and Br atoms were represented with sdd pseudopotencial. Frequency calculations were performed at the same level of theory to characterize the stationary points as minima (no imaginary frequencies) or transition states (one imaginary frequency), as well as to calculate free energy (G) corrections. The two minima connected by a given transition state were confirmed by manually freezing the coordinates at both sides of the imaginary frequency and further optimization at the theory level stated above. The matrix coordinates of all reactants, products and TSs computed are given as xyz files in a compressed folder.

Table S5: Energies free anion, host and adducts of P(V) species.

	E (a.u.)	zpe	H (a.u.)	S (cal/kmol)	G (a.u.)
**2-S**	-2998.7458	0.834495	-2997.8607	271.475	-2997.9897
**2-C**	-2998.7295	0.832668	-2997.8454	281.137	-2997.9789
**3-S**	-4290.5374	0.826108	-4289.6590	276.485	-4289.7904
**3-C**	-4290.5256	0.824893	-4289.6478	284.172	-4289.7829
**NO_3^-**	-280.32020	0.014484	-280.30164	62.197	-280.33119
**H_2SO_4**	-699.65696	0.026728	-699.62423	73.878	-699.65933
**Cl^-**	-460.18045	0.00000	-460.17809	36.586	-460.19547
**Br^-**	-13.490521	0.00000	-13.488161	39.012	-13.506697
**I^-**	-11.541591	0.00000	-11.539231	40.428	-11.558439
**DMSO**	-553.16247	0.079172	-553.07670	73.506	-553.11163
**2-C + Cl^-**	-3459.0319	0.834541	-3458.1451	281.323	-3458.2788
**2-C + Br^-**	-3012.3029	0.833905	-3011.4164	284.769	-3011.552
**2-C + I^-**	-3010.3465	0.833728	-3009.460	286.208	-3009.5960
**2-C + HSO_4^-**	-3698.4823	0.862974	-3697.5625	304.307	-3697.7071
**2-C + NO_3^-**	-3279.1511	0.850393	-3278.2459	295.045	-3278.3860
**2-C + DMSO**	-3551.9314	0.915677	-3550.9582	304.569	-3551.1029
**2-S + DMSO**	-3551.9544	0.916055	-3550.9810	304.423	-3551.1256
**3-C + Cl^-**	-4750.8343	0.826874	-4749.9537	283.298	-4750.0883
**3-S + Cl^-**	-4750.8203	0.826412	-4749.9404	280.235	-4750.0736
**3-C + Br^-**	-4304.1033	0.826002	-4303.2232	286.807	-4303.3595
**3-S + Br^-**	-4304.0936	0.826050	-4303.2138	283.614	-4303.3486
**3-C + I^-**	-4302.1452	0.825719	-4301.2652	288.087	-4301.4021
**3-S + I^-**	-4302.1374	0.826141	-4301.2570	284.728	-4301.3927
**3-C + HSO_4^-**	-4990.2818	0.855475	-4989.3683	303.832	-4989.5127
**3-C + NO_3^-**	-4570.9530	0.842961	-4570.0539	296.006	-4570.1945
**3-C + DMSO**	-4843.7294	0.907199	-4842.7630	306.7870	-4842.9087
**3-S + DMSO**	-4843.7439	0.908049	-4842.7770	306.443	-4842.9226

Table S6: Energy differences of the different conformations of **2** and **3** as well as their adducts.

	ΔG(kcal/mol)	
	**2**	**3**
**S *versus* C**	6.72	4.72
**C + Cl^-**	-65.47	-69.04
**S + Cl^-**	-	-55.02
**C + Br^-**	-41.41	-43.91
**S + Br^-**	-	-32.30
**C + I^-**	-36.78	-38.19
**S + I^-**	-	-27.56
**C + HSO_4^-**	-43.19	-44.26
**C + NO_3^-**	-47.61	-50.51
**C + DMSO**	-7.71	-8.97
**S + DMSO**	-15.27	-12.90

Table S7: Relative energies differences of the computed **ON/OFF** switching for **3**.

	ΔG(kcal/mol)
**C**	4.72
**S**	0.00
**TS**	12.57

Table S8: Energy differences of different ureas and their anion/DMSO adducts.

	ΔG(kcal/mol)		
	**Urea** 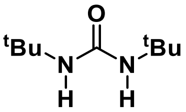	**Thiourea** 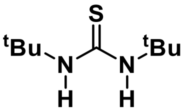	**Selenourea** 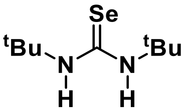
**Cl^-**	-34.29	-39.05	-39.95
**Br^-**	-17.62	-22.95	-23.07
**I^-**	-15.36	-19.62	-19.60
**HSO_4^-**	-17.13	-20.71	-21.07
**NO_3^-**	-22.27	-26.48	-26.85
**DMSO**	-5.33	-6.61	-6.04

Table S9: Energy differences of monomeric $P^{V}_{2}N_{2}$ species and their anion/DMSO adducts.

	ΔG(kcal/mol)		
	**2^m** 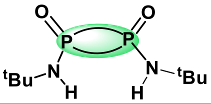	**3^m** 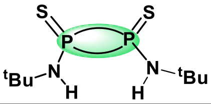	**4^m** 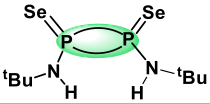
**Cl^-**	-47.11	-49.48	-50.42
**Br^-**	-25.32	-28.47	-29.04
**I^-**	-23.44	-24.31	-25.20
**HSO_4^-**	-27.34	-30.27	-30.92
**NO_3^-**	-32.73	-36.62	-37.37
**DMSO**	-8.40	-6.35	-6.40

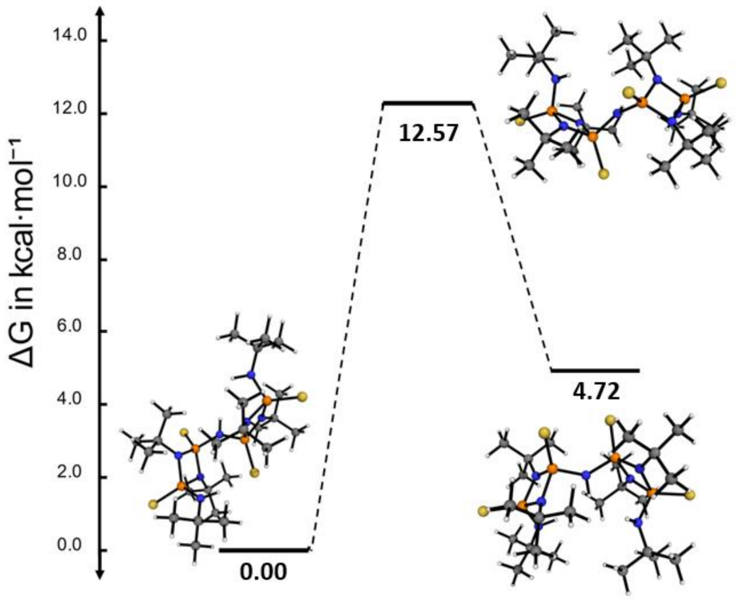

Figure S69. Energy profile of the topological conformational change $3^{OFF}/3^{ON}$

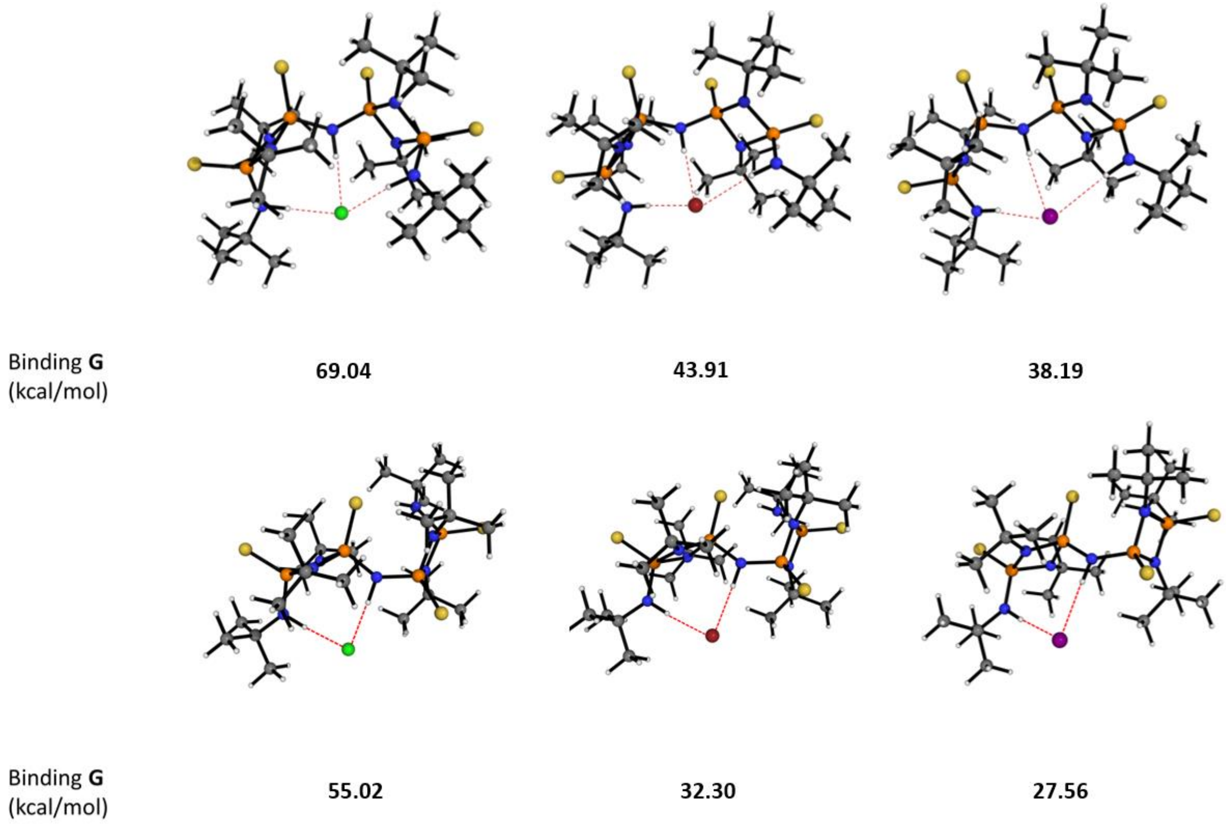

Figure S70. Calculated energies of 3^{ON} and 3^{OFF} with various halide anions.

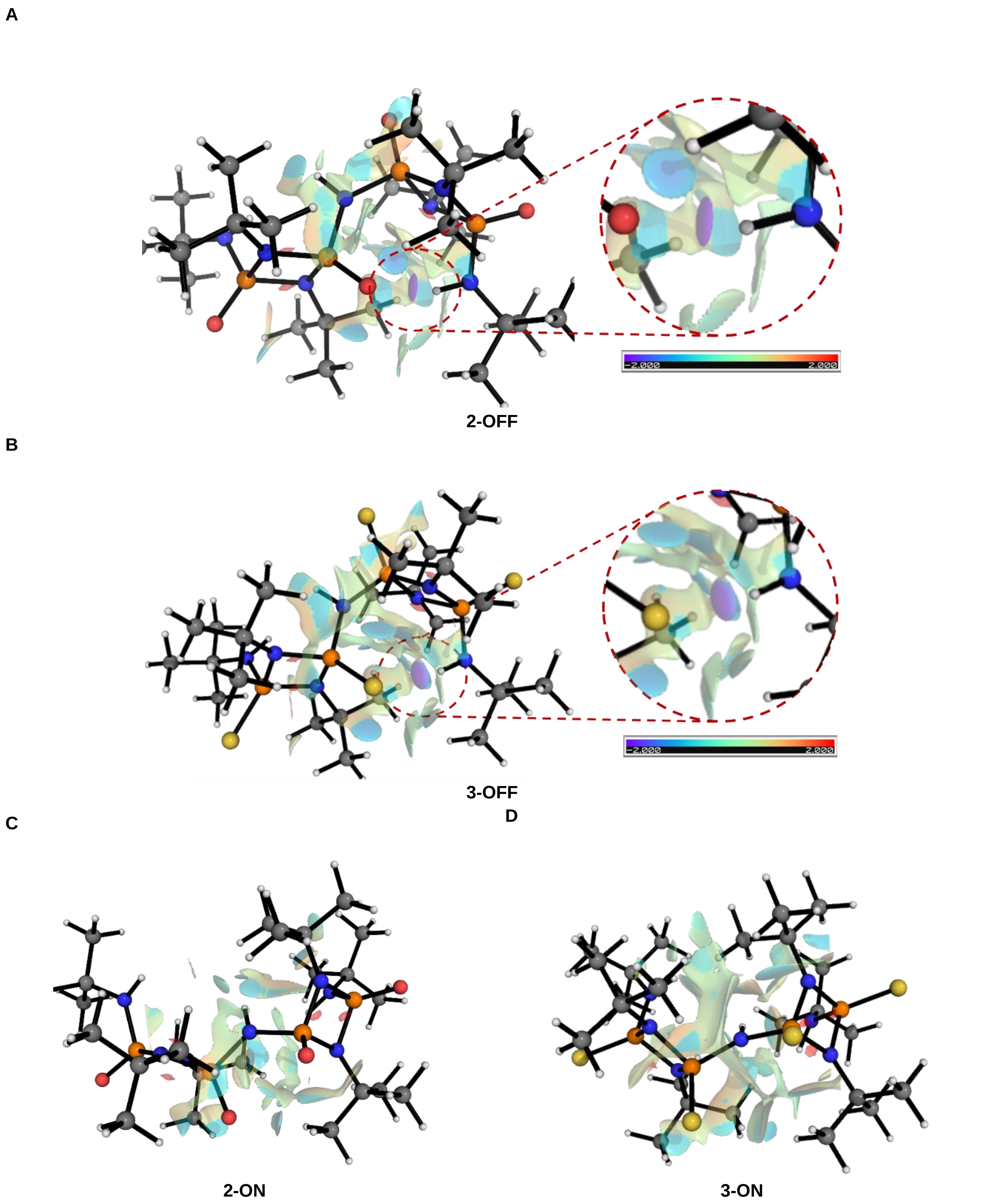

Figure S71. Non-Covalent Interaction (NCI) computed at B3PW91/6-311G(d,p). The weak interactions revealed by the NCI analysis are color coded from blue, for the strongest attractive weak interactions (like hydrogen bonds), to red for repulsive ones, for weak van der Waals interactions NCI interaction appears in green. NCI surfaces correspond to $\sigma = 0.5$ au and a colour scale of $-2 < \rho < 2$ au.

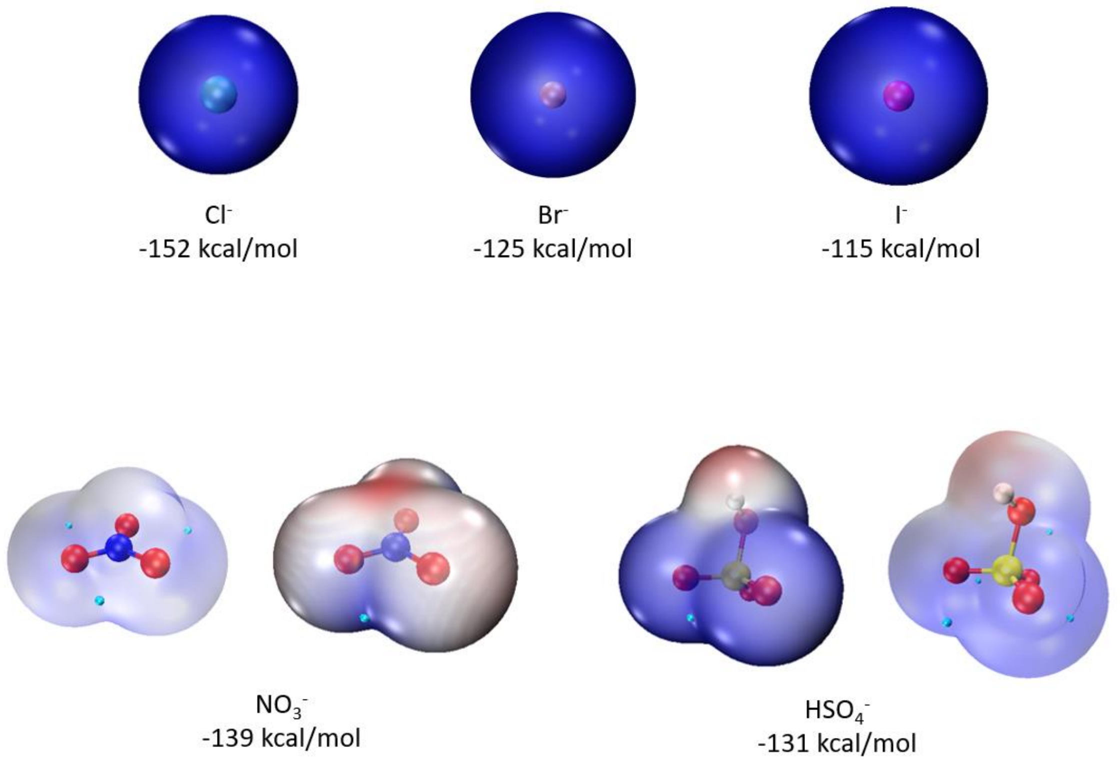

Figure S72. ESP surfaces of various mono and polyatomic anions plotted on the electronic density with isovalue = 0.01.

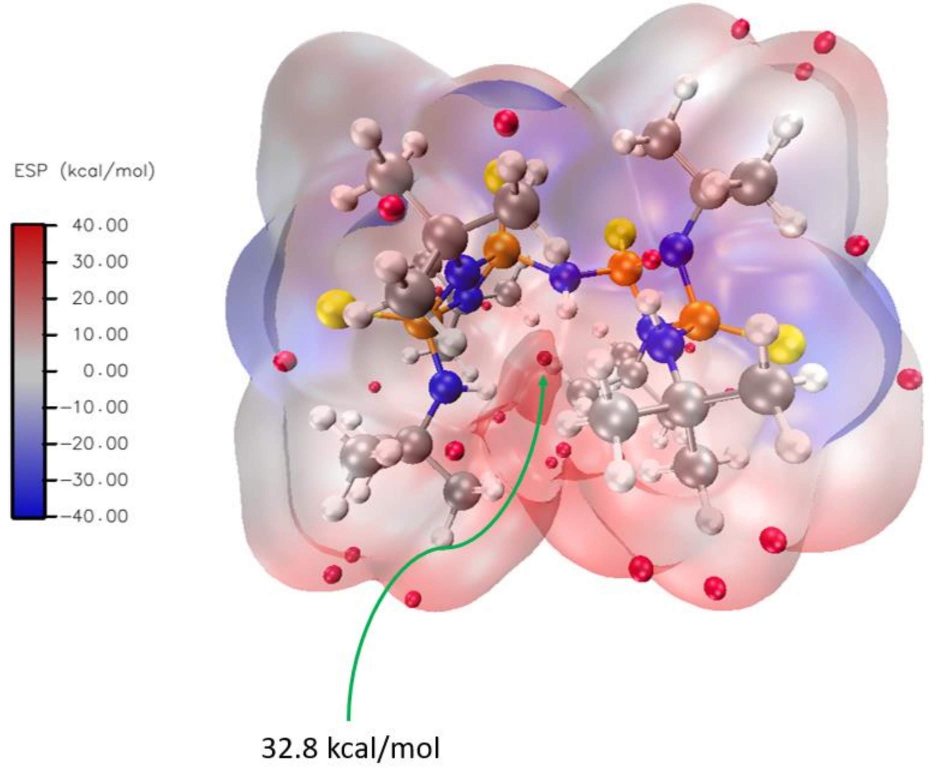

Figure S73. ESP surfaces of **3** plotted on the electronic density with isovalue = 0.01.

7. References

[1] G. R. Lief, D. F. Moser, L. Stahl, R. J. Staples, *J. Organomet. Chem.* **2004**, *689*, 1110-1121.
[2] T. G. Hill, R. C. Haltiwanger, M. L. Thompson, S. A. Katz, A. D. Norman, *Inorg. Chem.* **1994**, *33*, 1770-1777.
[3] D. Tan, Z. X. Ng, Y. Sim, R. Ganguly, F. García, *CrystEngComm* **2018**, *20*, 5998-6004.
[4] a) H. Günther, *NMR spectroscopy: basic principles, concepts and applications in chemistry*, John Wiley & Sons, **2013**; b) M. T. Huggins, T. Kesharwani, J. Buttrick, C. Nicholson, *J. Chem. Educ.* **2020**, *97*, 1425-1429.
[5] A. J. Plajer, J. Zhu, P. Proehm, A. D. Bond, U. F. Keyser, D. S. Wright, *J. Am. Chem. Soc.* **2019**, *141*, 8807-8815.
[6] Binding constants determined using Bindfit program available at http://supramolecular.org, for more information about association constants in supramolecular chemistry refer to (a) P. Thordarson, *Chem. Soc. Rev.*, **2011**, *40*, 1305-1323; (b) D. B. Hibbert, P. Thordarson, *Chem. Commun.*, **2016**, *52*, 12792-12805.
[7] SMART version 5.628; Bruker AXS Inc., Madison, WI, USA, 2001.
[8] Sheldrick, G. M. SADABS V2014/4 (Bruker AXS Inc.) University of Göttingen, Göttingen, Germany, 2014.
[9] SHELXL-2014/6 (Sheldrick, 2014) ; Bruker AXS Inc., Madison, WI, USA, 2014.
[10] Gaussian 16, Revision A.03, Frisch, M. J.; Trucks, G. W.; Schlegel, H. B.; Scuseria, G. E.; Robb, M. A.; Cheeseman, J. R.; Scalmani, G.; Barone, V.; Peters son, G. A.; Nakatsuji, H.; Li, X.; Caricato, M.; Marenich, A. V.; Bloino, J.; Janesko, B. G.; Gomperts, R.; Mennucci, B.; Hratchian, H. P.; Ortiz, J. V.; Izmaylov, A. F.; Sonnenberg, J. L.; Williams-Young, D.; Ding, F.; Lipparini, F.; Egidi, F.; Goings, J.; Peng, B.; Petrone, A.; Henderson, T.; Ranasinghe, D.; Zakrzewski, V. G.; Gao, J.; Rega, N.; Zheng, G.; Liang, W.; Hada, M.; Ehara, M.; Toyota, K.; Fukuda, R.; Hasegawa, J.; Ishida, M.; Nakajima, T.; Honda, Y.; Kitao, O.; Nakai, H.; Vreven, T.; Throssell,K.; Montgomery, Jr., J. A.; Peralta, J. E.; Ogliaro, F.; Bearpark, M. J.; Heyd, J. J.; Brothers, E. N.; Kudin, K. N.; Staroverov, V. N.; Keith, T. A.; Kobayashi, R.; Normand, J.; Raghavachari, K.; Rendell, A. P.; Burant, J. C.; Iyengar, S. S.; Tomasi, J.;Cossi, M.; Millam, J. M.; Klene, M.; Adamo, C.; Cammi, R.; Ochterski, J. W.; Martin, R. L.; Morokuma, K.; Farkas, O.; Foresman, J. B.; Fox, D. J. Gaussian, Inc., Wallingford CT, 2016.
[11] J. P. Perdew, in Electronic Structure of Solids '91, Ed. P. Ziesche and H. Eschrig (Akademie Verlag, Berlin, 1991) 11.
[12] S. Grimme, S. Ehrlich and L. Goerigk, "Effect of the damping function in dispersion corrected density functional theory," J. Comp. Chem. 32 (2011) 1456-65.
[13] a) J. Contreras-Garcia, E. Johnson, S. Keinan, R. Chaudret, J-P Piquemal, D. Beratan, W. Yang, *J. Chem. Theor. Comp.* **2011**, *7*, 625-632; b) E R. Johnson, S. Keinan, P. Mori-Sanchez, J. Contreras-Garcia, A J. Cohen, and W. Yang, *J. Am. Chem. Soc.* **2010**, *132*, 6498-6506.
